# Comparison of the Cox Proportional Hazards Model and Random Survival Forest Algorithm for Predicting Patient‐Specific Survival Probabilities in Clinical Trial Data

**DOI:** 10.1002/bimj.70171

**Published:** 2026-09-25

**Authors:** Ricarda Graf, Susan Todd, M. Fazil Baksh

**Affiliations:** ^1^ Institute of Mathematics University of Augsburg Augsburg Germany; ^2^ Department of Mathematics and Statistics University of Reading Reading UK

**Keywords:** Cox regression, Random survival forest, Randomized controlled trials, Simulation study

## Abstract

The Cox proportional hazards model is often used to analyze data from randomized controlled trials (RCTs) with time‐to‐event outcomes. Random survival forest (RSF) is a machine‐learning algorithm known for its high predictive performance. We conduct a comprehensive neutral comparison study to compare the performance of Cox regression and RSF in various simulation scenarios based on two reference datasets from RCTs. The motivation is to identify settings in which one method is preferable over the other when comparing different aspects of performance using measures according to the TRIPOD (Transparent Reporting of a multivariable prediction model for Individual Prognosis or Diagnosis) recommendations. Our results show that conclusions solely based on the C index, a performance measure that has been predominantly used in previous studies comparing predictive accuracy of the Cox‐PH and RSF model based on real‐world observational time‐to‐event data and that has been criticized by methodologists, may not be generalizable to other aspects of predictive performance. We found that measures of overall performance may generally give more reasonable results, and that the standard log‐rank splitting rule used for the RSF may be outperformed by alternative splitting rules, in particular in nonproportional hazards settings. In our simulations, the performance of the RSF suffers less in data with treatment–covariate interactions compared to data where these are absent. The performance of the Cox‐PH model is affected by the violation of the proportional hazards assumption.

## 1 Introduction

Prognostic prediction models are used to estimate an individual's probability, based on multiple risk factors, that a disease or outcome will occur in a specific period of time. They are most often used at the time of diagnosis or start of treatment to support physicians in early detection, diagnosis, treatment decisions, and prognosis, and to inform patients about their risks (Moons et al. 2012). They are applied in the medical field in general, and in particular in the field of cancer treatment and research, the field of diabetes, and the cardiovascular field (Goldstein et al. 2017; Moons et al. 2012). Clinical decision tools such as “ClinicalPath” (Elsevier 2022) for cancer treatment or the Framingham Risk Score (Wilson et al. 1998) for coronary heart disease are examples of prognostic prediction models.

Cox regression (Cox 1972) is most widely used for developing prognostic models in medical time‐to‐event data (Collins et al. 2014, 2011; Goldstein et al. 2017; Hueting et al. 2022; Mahar et al. 2017; Mallett et al. 2010; Phung et al. 2019; Steyerberg et al. 2013; Wynants et al. 2019). It provides estimates of the hazard ratios for each explanatory variable. In the context of clinical trials, the treatment effect hazard ratio is of particular interest. As a semi‐parametric model, it is assumed that at least 10 events have to be observed per predictor variable included in the Cox model to obtain reasonable results (Peduzzi et al. 1995; Vittinghoff and McCulloch 2006), so it cannot be used in high‐dimensional settings with a large number of potential predictor variables compared to the number of individuals. During model development, researchers often have to decide on a fraction of available predictors to be included in the final model (Moons et al. 2012). Cox regression requires choice of terms to include in a model, including possible (higher order) interaction terms and variable transformations in case of nonlinear relationships of continuous covariates with the survival outcome. Moreover, it makes the assumption of proportional hazards, which means that it assumes the hazard ratio of any two patients to be constant over the period of follow‐up. In cases where the new treatment only shows an advantage at an early or later stage, respectively, interpretation of its results may not be meaningful. Especially in long‐term studies, this assumption may be violated (Hilsenbeck et al. 1998). On the other hand, the Cox model provides corresponding measures of uncertainty (confidence intervals for the hazard ratios), which generally form the basis for clinical decision‐making, is easy to use and has short computational times. When using the Cox model for predictions, the specification of a baseline survival distribution is required (Therneau and Grambsch 2000).

In comparison, the Random survival forest (RSF) algorithm (Ishwaran et al. 2008) is a nonparametric machine‐learning approach. It is suitable for the same variable types as the Cox model, that is, continuous right‐censored survival time outcomes and continuous as well as categorical predictor variables. In contrast, it does not require an explicit specification of a model but is able to detect and incorporate even complex interactions between the covariates and the survival outcome as well as nonlinear relationships. It is also suitable for a large number of covariates, although it is advisable not to include variables that are already suspected not to be meaningful in order to not unnecessarily increasing computational complexity. It also seems suitable for dependent censoring (Zhou and Mcardle 2015). Moreover, it does not require the proportional hazards assumption. However, since the RSF does not make parametric assumptions regarding the data, it also does not provide uncertainty measures such as confidence intervals for its estimates. Machine‐learning methods such as random forests have proven to increase predictive accuracy in prognostic studies (Murmu and Győrffy 2024), especially in high‐dimensional data such as genetic, protein, or imaging biomarkers (Cohen et al. 2018; Kawakami et al. 2019; Lin et al. 2022; Ruyssinck et al. 2016; Zhang et al. 2020). For example, a prognostic model for glioblastoma, widely used for more than two decades and most recently adapted to incorporate further relevant covariates (Bell et al. 2017), is based on a survival tree method. The RSF may help in predicting patient outcomes and survival rates more accurately. Therefore, the current work aims to explore the potential application of the RSF to data from randomized controlled trials (RCTs) by comparing its predictive performance to the Cox proportional hazards (Cox‐PH) model.

Previous studies compared the performance of Cox regression and RSF in observational clinical data, more specifically real‐world datasets (e.g., Chowdhury et al. 2023; Datema et al. 2012; Du et al. 2020; Farhadian et al. 2021; Guo et al. 2023; Miao et al. 2015; Moncada‐Torres et al. 2021; Kim et al. 2019; Omurlu et al. 2009; Qiu et al. 2020; Sarica et al. 2023; Spooner et al. 2020). The predominantly used performance measure in these studies is Harrell's C index (Harrell et al. 1982, 1996), a rank‐based measure of discrimination. Very rarely, calibration, or overall performance, is assessed. Only the study by Du et al. (2020) considered all three recommended types of performance measures. In their systematic review and meta‐analysis of 52 studies predicting hypertension, Chowdhury et al. (2022) compared the performance of regression approaches (including Cox regression) and various machine‐learning methods (RSF was not applied in any of the studies). These authors also found that performance comparison based on the C index was common, in contrast to comparisons based on calibration. Most of the above‐mentioned studies aiming to compare the performance of the Cox and RSF models stated at least slightly better performance of the RSF model with respect to the C index.

According to our literature search, only one study previously compared the two approaches based on data simulations (Baralou et al. 2022), for which reference data are taken from an observational study. Moreover, their comparison is not only based on the default log‐rank splitting rule for the RSF, but includes two further splitting rules. In addition to a measure of discrimination (time‐dependent area under the curve, AUC), they also use a measure of overall performance (Integrated Brier score, IBS; Graf et al. 1999). Most notably, they found that the RSF outperformed the Cox‐PH model in scenarios with lower censoring rates in the presence of covariate interactions. However, they do not examine the performance for data from RCTs (including factors specific to RCTs such as different sizes of treatment effect, the absence/presence of treatment–covariate interactions, and smaller sample sizes less than 500), the influence of violation of the proportional hazards assumption, other splitting rules available for the RSF, and measures of calibration.

The TRIPOD (Transparent Reporting of a multivariable prediction model for Individual Prognosis or Diagnosis) recommendations (Moons et al. 2015) state that prognostic models should be compared with respect to discrimination (e.g., Harrell's C index, time‐dependent AUC for time‐to‐event data), calibration, and overall performance (e.g., IBS). These three aspects of model performance are also described in Steyerberg et al. (2010) and McLernon et al. (2023), for example. Here, Harrell's C index, calibration curves, and the IBS will be used as performance measures, which are described in Section 2.3.

Responsible integration of machine learning algorithms in any step of a clinical trial may help overcome some of the challenges in its design, conduct, and analysis, for example, with respect to patient recruitment or the planning of treatment interventions (Miller et al. 2023; Weissler et al. 2021). Evidence is needed on where machine learning algorithms can be applied in order to gain an advantage, such as more precise predictions free from parametric model assumptions.

Our simulation study may be the first one comparing the performance of the Cox‐PH and RSF models for clinical trial settings. The aim is to evaluate the predictive accuracy of both methods, the Cox regression model and the RSF algorithm, in predicting patient‐specific survival probabilities in right‐censored clinical trial data. Two possible scenarios are considered, where treatment–covariate interactions in the data are either absent or present. For this purpose, two publicly available clinical trial datasets (Therneau and Lumley 2024; Byar and Green 1980) without and with known treatment–covariate interactions serve as a reference for data simulations. In contrast to previous studies, the performance of all six RSF splitting rules (currently available in the most commonly used R packages randomForestSRC (Ishwaran and Kogalur 2023) and ranger (Wright et al. 2023) is compared, and evaluation is based on measures of discrimination, calibration, and overall performance for a more detailed comparison. Values for censoring rate, sample sizes, size of treatment effect, and shape of the hazard function are varied.

## 2 Materials and Methods

### 2.1 Reference Datasets

Two clinical trial datasets serve as a reference for data simulations. The first dataset does not have any known treatment–covariate interactions, and the second one comprises multiple treatment–covariate interactions. Despite our simulations being based on parameters estimated from two real‐world datasets, the main goal of our investigation is the comparison of two statistical prediction approaches for time‐to‐event data, rather than developing or validating models for the data themselves. The investigation seeks to assist applied researchers in assessing scenarios where fitting an RSF model may be useful, either in addition to the Cox model or possibly as an alternative in scenarios where the Cox model might perform particularly badly. Therefore, we try to mimic realistic data instead of using purely synthetic data, which would require a somewhat arbitrary choice of reasonable parameter combinations. In order to not limit our simulation study to two specific settings, we vary values of different parameters as they may occur in other real‐world datasets; more specifically, we consider different shapes of the hazard function, sample sizes, sizes of the treatment effect, and censoring rate.

We chose two inherently different examples. The first dataset (Section 2.1.1) is an RCT in primary biliary cirrhosis (PBC) patients, which gave rise to the Mayo Risk Score (Mayo Clinic 2026), and thus is an example of an adequately sized trial that has been used to develop a prediction model. The second dataset (Section 2.1.2) is a well‐known RCT in prostate cancer patients (Byar 1980; Byar and Corle 1988). Although not specifically used to create a prediction model, these data have treatment–covariate interactions, which we were particularly keen to explore, and the data have been studied over a number of years (Lester and Mason 2015; Oh et al. 2003; Woodhouse 2021).

#### 2.1.1 Data Without Treatment–Covariate Interactions: RCT in PBC

An RCT conducted by the Mayo Clinic between 1974 and 1984 (Therneau and Lumley 2024) investigates the effect of D‐penicillamine on survival times in 312 patients with PBC, with time to the occurrence of death, or liver transplantation, respectively, as the event of interest. A total of 16 prognostic factors were recorded, of which 10 were continuous and six were categorical variables. The median follow‐up time is about 5 years. Table 1A shows more detailed summary statistics. We replaced missing values in three of the continuous covariates by their column means; that is, incomplete data are included for estimating the correlation structure and fitting univariate parametric distributions to the data.

**TABLE 1A:** RCT in PBC: summary statistics of baseline measurements in 312 patients in the study conducted by the Mayo Clinic. Abbreviations: DPA, d‐penicillamine; SGOT, serum glutamic‐oxaloacetic transaminase. 0 = no edema and no diuretic therapy for edema; 0.5 = edema present for which no diuretic therapy was given or edema resolved with diuretic therapy; 1 = edema despite diuretic therapy.

	Median	Mean (SE)	Minimum	Maximum	# missing values
Survival time					
Time of follow‐up (days)	1839.5	2006.4 (1123.3)	41	4556	—
Continuous prognostic factor					
Age (years)	49.8	50 (10.6)	26.3	78.4	—
Serum bilirubin (mg/dL)	1.4	3.3 (4.5)	0.3	28	—
Serum cholesterol (mg/dL)	322	369.6 (221.3)	120	1775	—
Albumin (gm/dL)	3.5	3.5 (0.4)	2	4.6	—
Urine copper (mg/day)	73	97.6 (85.6)	4	588	2
Alkaline phosphatase (U/L)	1259	1982.7 (2140.4)	289	13862.4	—
Aspartate aminotransferase ‐ SGOT (U/mL)	114.7	122.6 (56.7)	26.4	457.2	—
Triglycerides (mg/dL)	108	124.7 (65.1)	33	598	30
Platelet count (# platelets per $m^{3}$/1000)	257	261.9 (95.6)	62	563	4
Prothrombin time (s)	10.6	10.7 (1)	9	17.1	—
	Levels				# missing values
Event indicator, treatment code					
Event indicator (0: censored, 1: death)	0: 59.9%	1: 40.1%			—
Treatment code (1: DPA, 2: placebo)	1: 50.6%	2: 49.4%			—
Categorical prognostic factors					
Sex (0: male, 1: female)	0: 11.5%	1: 88.5%			—
Presence of ascites (0: no, 1: yes)	0: 92.3%	1: 0.07%			—
Presence of hepatomegaly (0: no, 1: yes)	0: 48.7%	1: 51.3%			—
Presence of spiders (0: no, 1: yes)	0: 71.2%	1: 28.8%			—
Presence of ${\text{edema}}^{a}$	0: 84.3%	0.5: 9.3%	1: 6.4%		—
Histologic state of disease (grade)	1: 5.1%	2: 21.5%	3: 38.5%	4: 34.9%	—

Performance comparison of the Cox model to a non‐parametric alternative such as the RSF is motivated by the violation of the proportional hazards assumption in some datasets on which Cox regression is based. For instance, in this RCT dataset, the overall assumption of proportional hazards would be violated ($\chi^{2}=20.86$, df = 8, p=0.0075; test by Grambsch and Therneau 1994; implemented in the function cox.zph from the R package survival by Therneau and Lumley 2024) after variable selection based on findings in the literature and the statistical measures Akaike information criterion and Bayesian information criterion, an approach a researcher examining these data would typically follow.

#### 2.1.2 Data With Treatment–Covariate Interactions: RCT in Prostate Cancer Patients

These data are one of four RCTs conducted by the VACURG (Veterans Administration Cooperative Urological Research Group) between 1960 and 1975, and comprise information on 474 patients with advanced prostate cancer for whom complete data are available in the RCT examining the effect of the synthetic estrogen drug diethyl stilboestrol on survival time. The placebo group comprises patients receiving either placebo or the lowest dose level; the treatment group comprises patients receiving one of two higher dose levels (Byar and Green 1980). Table 1B gives an overview of the data structure. For data simulations, we removed the binary variable cancer stage due to multicollinearity. Based on findings in the literature (Byar and Green 1980;  Royston, Patrick and Sauerbrei, Willi 2004), we included relevant interaction terms between treatment and the variables age, presence of bone metastases, and serum acid phosphatase, respectively. Again, in a model comprising all main effects and these three interaction terms, for example, the proportional hazards assumption would not be fulfilled ($\chi^{2}=22.2$, df = 12, p=0.0355; test by Grambsch and Therneau 1994).

**TABLE 1B:** RCT in prostate cancer patients: summary statistics of baseline measurements in 474 patients in the prostate cancer dataset.

	Median	Mean (SE)	Minimum	Maximum	# missing values
Survtime					
Time of follow‐up	33.5	36.3 (23.2)	0.5	76.5	—
Continuous prognostic factors					
Age (Years)	73	71.6 (6.9)	48	89	—
Standardized weight	98	99 (13.3)	69	152	—
Systolic blood pressure	14	14.4 (2.4)	8	30	—
Diastolic blood pressure	8	8.2 (1.5)	4	18	—
Size of primary tumor (${\text{cm}}^{2}$)	10	14.3 (12.2)	0	69	—
Serum (prostatic) acid phosphatase King Armstrong units)	7	125.7 (638.5)	1	9999	—
Hemoglobin (g/100 mL)	137	134.2 (19.4)	59	182	—
Gleason stage‐grade category (mg/dL)	10	10.3 (2)	5	15	—
	Levels				# missing values
Event indicator, treatment code					
Event indicator (0: censored, 1: death)	0: 28.8%	1: 71.2%			—
Treatment code (0: lowest dose of diethylstilboestrol (placebo), 1: higher doses)	0: 49.9%	1: 50.1%			—
Binary prognostic factors					
Performance status	0: 90.1%	1: 9.9%			—
History of cardiovascular disease (0: no, 1: yes)	0: 56.6%	1: 43.4%			—
Presence of bone metastases (0: no, 1: yes)	0: 83.8%	1: 16.2%			—
Abnormal electrocardiogram (0: normal, 1: abnormal)	0: 34.1%	1: 65.9%			—

### 2.2 Methods for Performance Comparison

The approaches were first compared using the bootstrap technique by Wahl et al. (2016), which is based on the work by Jiang et al. (2008). This is an internal validation technique based on the real data for estimating point estimates of the performance measures and corresponding confidence intervals (CIs). It is described in Section 2.2.1. Moreover, we used data simulations, which facilitate manipulations of data properties but at the same time require specification of data‐generating mechanisms. The approach is described in Section 2.2.2. Model building for finding the most suitable model for each dataset was done in the same way for both the bootstrap and simulated data. Details are given in Supporting Information Section A.

#### 2.2.1 Nonparametric Bootstrap Approach

The nonparametric bootstrap approach for point estimates by Wahl et al. (2016) is an extension of the algorithm by Jiang et al. (2008) and is based on the 0.632+ bootstrap method (Efron and Tibshirani 1997).

The 0.632+ bootstrap estimate (${\hat{\theta }}^{0.632+}$) of the performance measure of interest is computed as a weighted average of the apparent performance ${\hat{\theta }}^{orig,orig}$ (training and test data given by the original dataset) and the average “out‐of‐bag” (OOB) performance ${\hat{\theta }}^{bootstrap,OOB}=\sum_{b=1}^{B}{\hat{\theta }}_{b}^{bootstrap,OOB}$ computed from B bootstrap datasets (training data given by the bootstrap dataset, and test data given by the samples not present in the bootstrap dataset). The formula is

$${\hat{\theta }}^{0.632+}=\left(1-w\right)\cdot {\hat{\theta }}^{orig,orig}+w\cdot {\hat{\theta }}^{bootstrap,OOB},$$

where $w=\frac{0.632}{1-0.368\cdot R}$ and *R* = $\frac{{\hat{\theta }}^{bootstrap,OOB}-{\hat{\theta }}^{orig,orig}}{\theta^{noinfo}-{\hat{\theta }}^{orig,orig}}$. In the case of the C index, $\theta^{noinfo}=0.5$. For the IBS, $\theta^{noinfo}=0.25$. Then, each bootstrap dataset is assigned a weight $w_{b}={\hat{\theta }}_{b}^{bootstrap,bootstrap}-{\hat{\theta }}^{orig,orig}$, where ${\hat{\theta }}_{b}^{bootstrap,bootstrap}$ is the value of the performance measure when the bootstrap dataset b∈{1,…,B} is used as training as well as test dataset. The $\frac{\alpha }{2}$ and $1-\frac{\alpha }{2}$ percentiles of the empirical distribution of these weights, $\xi_{\frac{\alpha }{2}}$ and $\xi_{1-\frac{\alpha }{2}}$, give the CI of ${\hat{\theta }}^{0.632+}$

$$[{\hat{\theta }}^{0.632+}-\xi_{1-\frac{\alpha }{2}},{\hat{\theta }}^{0.632+}+\xi_{\frac{\alpha }{2}}].$$

#### 2.2.2 Data Simulation

For data simulations, covariate data similar to the reference data are generated by using a copula model. The specific distributions and corresponding parameters can be found in the Supporting Information Section B's Tables B.1a and B.1b show the correlation matrices estimated from the PBC and the prostate cancer dataset, respectively. Tables B.2a and B.2b of Supporting Information Section B show the assumed parametric distributions of each variable in both reference datasets. In Figures B.1 and B.2 of Supporting Information Section B, the empirical variable distributions and the best fitting theoretical distributions based on maximum likelihood estimation are shown for the PBC and the prostate cancer dataset, respectively. Covariate‐dependent survival times were generated from a Cox proportional hazards model assuming Weibull(λ,γ) distributed survival times according to the cumulative hazard inversion method by Bender et al. (2005) implemented in the R package simsurv (Brilleman and Gasparini 2022). For this, regression parameters β were estimated based on the reference datasets. For the PBC dataset

$$\begin{array}{cc} \beta^{\text{PBC}}=\; & \left(\beta_{1},\cdots ,\beta_{17}\right)\\ =\; & (\beta_{Z1},\beta_{Z2},\beta_{Z3},\beta_{Z4},\beta_{Z5},\beta_{Z6},\beta_{Z7},\beta_{Z8},\\ & \beta_{Z9},\beta_{Z10},\beta_{Z11},\beta_{Z12},\beta_{Z13},\beta_{Z14},\beta_{Z15},\beta_{Z16},\beta_{Z17})\\ \approx \; & (\beta_{Z1},\;0.026,\;-0.218,\;0.338,\;0.227,\;0.071,\;0.481,\;0.086,\\ & 0.0004,\;-0.799,\;0.003,\;-0.00002,\;0.004,\;\\ & -0.002,\;0.0002,\;0.276,\;0.365). \end{array}$$

For the prostate cancer dataset

$$\begin{array}{cc} \beta^{\text{PC}}=\; & \left(\beta_{1},\cdots ,\beta_{16}\right)\\ =\; & (\beta_{RX},\beta_{AGE},\beta_{WT},\beta_{SBP},\beta_{DBP},\beta_{SZ},\beta_{AP},\beta_{HG},\\ & \beta_{SG},\beta_{PF},\beta_{HX},\beta_{BM},\beta_{ECG},\beta_{RX:AGE},\beta_{RX:BM},\beta_{RX:AP})\\ \approx \; & (\beta_{RX},\;-0.006,\;-0.01,\;-0.016,\;0.02,\;0.014,\;0.0001,\;-0.006,\\ & 0.074,\;0.333,\;0.467,\;0.63,\;0.316,\;0.059,\;-0.612,\;-0.0003). \end{array}$$

Scale parameters λ were fixed at the value estimated from the respective reference dataset (λ = 2241.74 for the PBC dataset, λ = 39.2 for the prostate cancer dataset), shape parameters γ were varied in order to create scenarios with decreasing (γ=0.8), constant (γ=1), increasing (γ=2), and nonproportional hazards, that is, different values per treatment group ($\gamma_{0}=2,\gamma_{1}=5$). Random censoring times were generated from a uniform distribution $U_{\left[0,b\right]}$ such that censoring percentages of 30% and 60%, respectively, corresponding to the actual censoring rates in the two reference datasets, were obtained. For this, the approach by Ramos et al. (2024) was used, but in some cases the values of the distribution parameter b had to be manually adjusted. Total sample sizes N∈{100,200,400} were considered for the $n_{\text{sim}}=500$ training datasets. Our aim in conducting this simulation study is to explore settings in which machine‐learning approaches may help when assumptions of Cox regression are violated, that is, small sample sizes, violation of the proportional hazards assumption, existence of unknown or higher‐order interactions. The RSF is known to perform well in high‐dimensional settings. We confirmed our choice of sample sizes based on sample size calculation for multivariable prediction models with time‐to‐event outcomes (Ensor 2025; Riley et al. 2019), according to which, minimum sample sizes for the PBC and prostate cancer study are N=337 and N=713, respectively. Except for the case of N=400 for the PBC data, all sample sizes are small, which are our scenarios of interest.

For the $n_{\text{sim}}=500$ independent test datasets, the total sample size is N=500. In contrast to analyzing real‐world data, where the available observations are split into a training and test dataset, possibly several times in order to perform cross‐validation depending on the size of the dataset, simulations do not rely on actual data. Thus, independent test datasets can be generated (Graf et al. 2025). In simulations based on time‐to‐event data, this additionally provides the advantage of maintaining a certain censoring rate in both the training and test data. Moreover, different values of the treatment effect ($\beta_{\text{treatment}}\in \left\{0,0.8,-0.4\right\}$) were considered when generating the data corresponding to different hazard ratios of the treatment effect. For the RSF, all available splitting rules are included in the method comparison (overview in Table A.1 of Supporting Information Section A). Computational times per algorithm were measured including variable selection (for the Cox model) and hyperparameter tuning (for the RSF model), respectively.

### 2.3 Performance Measures

According to recommendations, performance metrics measuring discrimination, calibration, and overall performance shall be reported when comparing prediction models. In the context of survival analysis, discrimination refers to the model's ability to distinguish between patients with higher and lower risk of the outcome. Calibration compares predicted survival probabilities to the observed event frequencies in a given time interval. Overall performance encompasses both discrimination as well as calibration of the model. Some performance measures have been extended for use with survival outcomes.

#### 2.3.1 Measure of Discrimination: Harrell's C Index

The C index was originally developed for binary outcomes (Greenberg and Sen 1985), and has been subject to criticism (Hartman et al. 2023). It compares, for each pair of patients, whether the one with the shorter event time also has the higher predicted risk of suffering the event. These rank‐based comparisons may favor the model with the more inaccurate predictions (Vickers and Cronin 2010) and may not adequately reflect the influence different sets of covariates have on the outcome (Cook 2007), such that its interpretation may be misleading and not clinically meaningful for survival outcomes.

Alternative measures to the C index are time‐dependent receiver operating characteristic (ROC) curves or the time‐dependent AUC (Heagerty et al. 2004). Unlike the C index and the IBS (described below), they do not provide an overall summary of model performance over the entire time range, but require the selection of a specific time point, for example, the median follow‐up time. Depending on the choice of this time point, there may be larger differences in the evaluation of a model's performance. Despite its drawbacks, we will use the C index due to better comparability with the IBS since they are both time‐range performance measures.

The C index can be obtained from the Cox regression and RSF models as follows: For a Cox proportional hazards model,

$$h\left(t\right)=h_{0}\left(t\right)\exp\left(\beta_{1}x_{1}+\cdots +\beta_{d}x_{d}\right)$$

with baseline hazard function $h_{0}\left(t\right)$ and regression coefficients $\beta \in R^{d}$, and unique ordered survival times $t_{1},\cdots ,t_{m}$, at each uncensored survival time, the rank of the predicted outcome for the considered subject $j_{1}$ who experienced the event, that is, ${\hat{h}}_{j_{1}}\left(t\right)$, is compared to all ${\hat{h}}_{j_{2}}\left(t\right),j_{1}\neq j_{2}$ where individuals $j_{2}$ had a longer survival time. The C index can thus be written as

$$\text{Pr}\left({\hat{h}}_{j_{1}}>{\hat{h}}_{j_{2}}|T_{j_{1}}<T_{j_{2}}\right)=\frac{\sum_{j_{1}}\left(R_{j_{1}}-1\right)}{\sum_{j_{1}}\left(N_{j_{1}}-1\right)},\;j_{1},j_{2}\in \left\{1,\cdots m\right\},j_{1}\neq j_{2},$$

where $R_{j_{1}}$ is the rank of individual $j_{1}$ with survival time $T_{j_{1}}$, $N_{j_{1}}$ the number at risk at time $T_{j_{1}}$, and thus $N_{j_{1}}-1$ the number of individuals who can be compared with $j_{1}$ (Kremers and von Liebig 2007).

For the RSF model, the C index is computed based on the patient‐specific predictions of the ensemble mortality in the terminal nodes of each tree. For this, the OOB ensemble estimator of the cumulative hazard function (CHF) at time t for patient j, $H_{j}^{oob}\left(t\right)$, is considered. It is given by the average prediction of the $n_{{\text{tree}}_{j}}$ trees for which the sample was not part of the bootstrap sample for building the tree (Ishwaran et al. 2021):

$$H_{j}^{oob}\left(t\right)=\frac{1}{n_{{\text{tree}}_{j}}}\sum_{b\in n_{{\text{tree}}_{j}}}H_{b}\left(t|X\right),$$

where $H_{b}\left(t|X\right)$ is the CHF predicted in the terminal node of the bth tree for the covariate vector $X\in R^{d}$ of patient j at time t. The OOB ensemble mortality for each patient j=1,⋯,N is then estimated as the sum of the OOB CHF estimates over all unique event times $t_{1},\cdots ,t_{m}$ in the training data (Ishwaran et al. 2021):

$$M_{j}^{oob}=\sum_{k=1}^{m}H_{j}^{oob}\left(t_{k}\right).$$

The C index is the proportion of concordant pairs among all pairs for which the decision can be made. If $M_{j_{1}}^{oob}>M_{j_{2}}^{oob}$ and patient $j_{1}$ has the shorter event time compared to patient $j_{2}$, or vice versa, the pair is concordant. The closer C index estimates are to 1, the better.

#### 2.3.2 Measure of Calibration: Calibration Curves

A calibration plot of observed versus predicted probabilities of mortality indicates deviation from perfect prediction, the more the slope deviates from the ideal line with slope 1 (Van Calster et al. 2019). It quantifies the agreement between the actual and predicted outcome within a specified duration of time. Austin et al. (2020) describe and implement an approach for estimating calibration curves for survival outcomes. The calibration curve used here is estimated based on Cox regression using restricted cubic splines.

#### 2.3.3 Measure of Overall Performance: IBS

The IBS (Graf et al. 1999) summarizes the Brier scores over time; that is, it is a time‐range performance measure. The Brier score calculates the difference between predicted and actual survival at a given time point, and thus values indicate better overall performance the closer they are to zero. It is implemented in the function integrated_brier_score in the R package survex (Spytek et al. 2024).

#### 2.3.4 Interpretation of Performance Measures

Consideration of the clinical relevance of predictive performance measures is crucial to determine if a predictive model's statistical accuracy translates to real‐world benefit. The metrics considered in this paper often have guideline values of “acceptable” accuracy; for example, a C‐index of 0.7 is considered useful (Gangaram Panday et al. 2026) and an IBS of someway below 0.25 is necessary to be valuable, since 0.25 corresponds to essentially an uninformative model (Steyerberg et al. 2010). However, what is good or meaningful from a clinical perspective depends heavily on the clinical context and rarity of the event under study. For example, an IBS of 0.15 might be excellent for a rare disease, but poor for a common disease. Such metrics are often argued to be most useful in the context they have been implemented in this research—as tools for comparing models and model approaches in the same population rather than interpreting a single score in isolation. However, magnitudes of improvement in model performance between different candidate models or approaches is difficult to interpret in terms of clinical decision‐making. It must be acknowledged that further validation beyond the scenarios considered in this paper would allow a deeper understanding of the appropriateness and generalizability of RSF above traditional Cox models across different clinical settings.

## 3 Results

### 3.1 Results of the Bootstrap Approach

**FIGURE 1:**
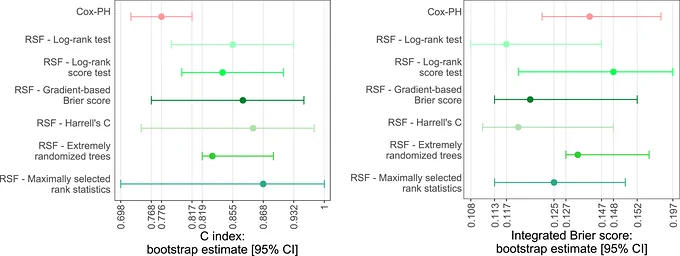
Bootstrap estimate ${\hat{\theta }}^{0.632+}$ (95% confidence interval) of the C index (right) and IBS (left) for the RCT in PBC patients. Abbreviations: Cox‐PH, Cox proportional hazards model; RSF, random survival forest.

**FIGURE 2:**
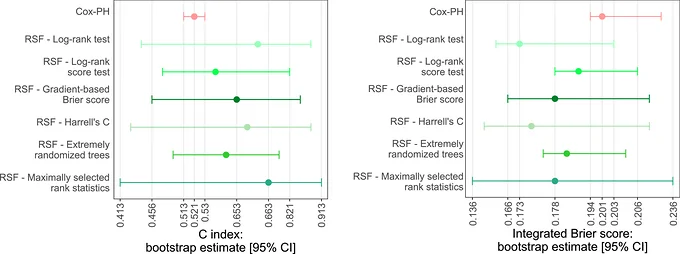
Bootstrap estimate ${\hat{\theta }}^{0.632+}$ (95% confidence interval) of the C index (left) and IBS (right) for the RCT in prostate cancer patients. Abbreviations: Cox‐PH, Cox proportional hazards model; RSF, random survival forest.

Tables 2 and 3 show the bootstrap estimates of the C index and IBS, respectively, when applying the bootstrap approach for point estimates (Jiang et al. 2008; Wahl et al. 2016) to both reference datasets. The same results are shown in Figure 1 (PBC dataset) and Figure 2 (prostate cancer dataset). The first impression is that the point estimates ${\hat{\theta }}^{0.632+}$ alone indicate a potentially better performance of most RSF models compared to the Cox‐PH model, but their confidence intervals are often much wider and mostly include the ${\hat{\theta }}^{0.632+}$ estimate of the Cox‐PH model. This is the case for both reference datasets.

**TABLE 2:** Bootstrap estimates ${\hat{\theta }}^{0.632+}$ (95% confidence interval) of the C index and IBS in the RCT data without treatment–covariate interactions (PBC dataset). Predictions are based on $n_{\text{sim}}$ = 1000 bootstrap datasets.

	Cox‐PH	Random survival forest					
		Log‐rank test	Log‐rank score	Gradient‐based Brier score	Harrell's C	Extremely randomized trees	Maximally selected rank statistics
C index	0.776 (0.735,0.817)	0.855 (0.778,0.932)	0.847 (0.815,0.88)	0.856 (0.768,0.944)	0.858 (0.764,0.953)	0.844 (0.819,0.869)	0.868 (0.698,1)
IBS	0.131 (0.124,0.161)	0.117 (0.108,0.147)	0.148 (0.118,0.197)	0.122 (0.113,0.152)	0.118 (0.11,0.148)	0.129 (0.127,0.153)	0.125 (0.113,0.151)

**TABLE 3:** Bootstrap estimates ${\hat{\theta }}^{0.632+}$ (95% confidence interval) of the C index and IBS in the RCT data with three treatment–covariate interactions (prostate cancer dataset). Predictions are based on $n_{\text{sim}}$ = 1000 bootstrap datasets.

	Cox‐PH	Random survival forest					
		Log‐rank test	Log‐rank score	Gradient‐based Brier score	Harrell's C	Extremely randomized trees	Maximally selected rank statistics
C index	0.521 (0.513,0.53)	0.66 (0.438,0.881)	0.642 (0.462,0.821)	0.653 (0.456,0.85)	0.657 (0.432,0.881)	0.645 (0.498,0.792)	0.663 (0.413,0.913)
IBS	0.201 (0.194,0.211)	0.173 (0.161,0.203)	0.184 (0.178,0.206)	0.178 (0.166,0.208)	0.174 (0.157,0.208)	0.18 (0.175,0.204)	0.178 (0.136,0.236)

The RSF (“log‐rank test”) may have an advantage over the Cox model when comparing overall performance (using the IBS) in both datasets, because its point estimates ${\hat{\theta }}^{0.632+}$ are the lowest and the corresponding confidence intervals have the smallest overlap with the confidence interval belonging to ${\hat{\theta }}^{0.632+}$ of the Cox‐PH model. With respect to the C index, the RSF (“extremely randomized trees”) performs better in the data without treatment–covariate interactions (PBC dataset). Confidence intervals are nonoverlapping for this approach and the Cox‐PH model. For the prostate cancer dataset, RSF may have better performance concluded from the point estimates alone, but the confidence intervals of the Cox and RSF models completely overlap such that no clear conclusion can be made.

### 3.2 Simulation Study Results

In this section, the simulation study results for one of the treatment effects considered in the simulation study ($\beta_{\text{treatment}}=-0.4$) are presented and discussed. The results for other values of the treatment effect ($\beta_{\text{treatment}}\in \left\{0,0.8\right\}$) are similar and can be found in the Supporting Information Section C. Moreover, only the results for the algorithms that are of most interest are shown, which are the Cox model and the RSF using the standard log‐rank test splitting rule. Additionally, the results of the RSF based on other splitting rules are shown if they outperform these two methods with respect to the median result. Only the best performing one among them is shown in case there are multiple better performing alternatives. Results for the remaining RSF splitting rules are shown in the Supporting Information Section C.

**FIGURE 3:**
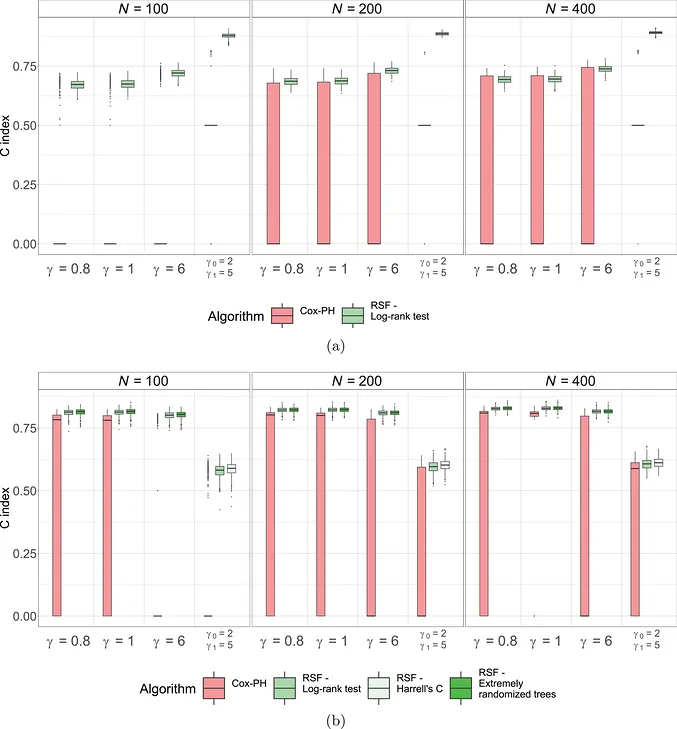
C index estimates in the simulation scenario assuming 30% censoring and a treatment effect of $\beta_{\text{treatment}}=-0.4$ for the RCT in PBC (a) and in prostate cancer patients (b). Survival times are generated from a Weibull distribution with scale parameters estimated from the respective reference dataset, shape parameters (γ) vary in order to examine the impact of differing hazards (γ = 0.8: decreasing hazard, γ = 1: constant hazard, γ = 6: increasing hazard, {$\gamma_{0}$ = 2, $\gamma_{1}$ = 5}: nonproportional hazards), and the violation of the proportional hazards assumption. Results are shown for different total sample sizes N. Abbreviations: Cox‐PH, Cox proportional hazards model; RSF, random survival forest.

**FIGURE 4:**
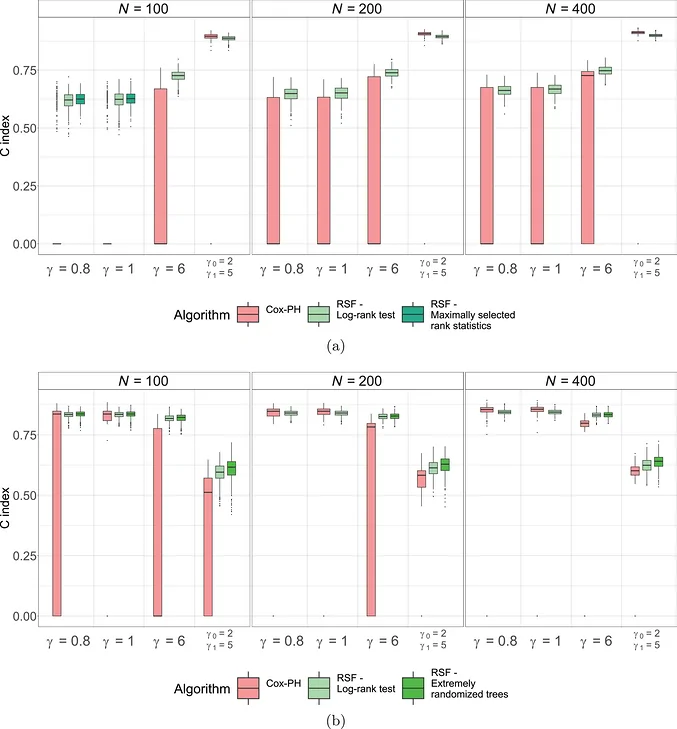
C index estimates in the simulation scenario assuming 60% of censoring and a treatment effect of $\beta_{\text{treatment}}=-0.4$ for the RCT in PBC (a) and in prostate cancer patients (b). Survival times are generated from a Weibull distribution with scale parameters estimated from the respective reference dataset, shape parameters (γ) vary in order to examine the impact of differing hazards (γ = 0.8: decreasing hazard, γ = 1: constant hazard, γ = 6: increasing hazard, {$\gamma_{0}$ = 2, $\gamma_{1}$ = 5}: nonproportional hazards), and the violation of the proportional hazards assumption. Results are shown for different total sample sizes N. Abbreviations: Cox‐PH, Cox proportional hazards model; RSF, random survival forest.

The C index estimates, which correspond to the models' discriminative performance, are shown in Figure 3 (30% censoring rate) and Figure 4 (60% censoring rate). Results for the RCT data without treatment–covariate interactions (PBC dataset) are shown in Figures 3a and 4a. For a censoring rate of 30%, varying hazards, and sample sizes, the RSF based on the log‐rank test splitting rule performs best. For a censoring rate of 60%, the Cox model performs best in the nonproportional hazards setting independent of sample size, and otherwise the RSF performs best: for a total sample size of N=100, the RSF using the “maximally selected rank statistics” splitting rule (slightly) outperforms the standard log‐rank test splitting rule in the scenarios assuming a decreasing and constant hazard. Otherwise, the log‐rank test splitting rule gives the best results. This may indicate that the (discriminative) performance of the RSF suffers more from higher censoring proportions in comparison to the Cox model. Results for the RCT data with multiple treatment–covariate interactions (prostate cancer dataset) are shown in Figures 3b and Figure 4b. For a censoring rate of 30%, the RSF using the “extremely randomized trees” splitting rule performs best in the proportional hazards setting, independent of sample size. For the nonproportional hazards setting, the RSF based on the “Harrell's C” splitting rule performs best. For a censoring rate of 60%, the Cox model performs best for the higher sample sizes (N=200 and N=400) for two of the proportional hazards settings (γ=0.8 and γ=1). For all other scenarios, the RSF based on the “extremely randomized trees” splitting rule performs best. In contrast to the scenario without treatment–covariate interactions, the RSF outperforms the Cox model with respect to discriminative performance in the case of nonproportional hazards. This may indicate the ability of the RSF to handle these interactions even without prior specification in the model.

**FIGURE 5:**
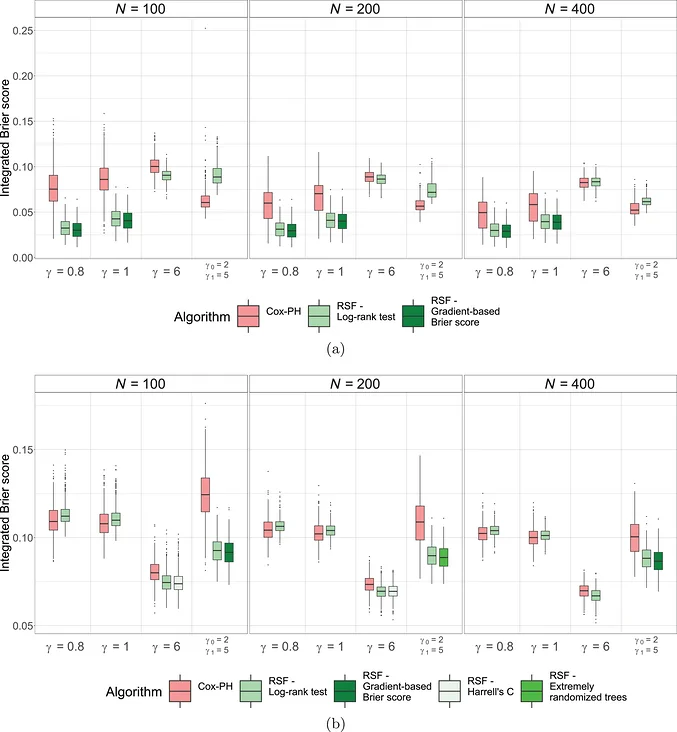
IBS estimates in the simulation scenario assuming 30% censoring and a treatment effect of $\beta_{\text{treatment}}=-0.4$ for the RCT in PBC (a) and in prostate cancer patients (b). Survival times are generated from a Weibull distribution with scale parameters estimated from the respective reference dataset, shape parameters (γ) vary in order to examine the impact of differing hazards (γ = 0.8: decreasing hazard, γ = 1: constant hazard, γ = 6: increasing hazard, {$\gamma_{0}$ = 2, $\gamma_{1}$ = 5}: nonproportional hazards), and the violation of the proportional hazards assumption. Results are shown for different total sample sizes N. Abbreviations: Cox‐PH, Cox proportional hazards model; RSF, random survival forest.

**FIGURE 6:**
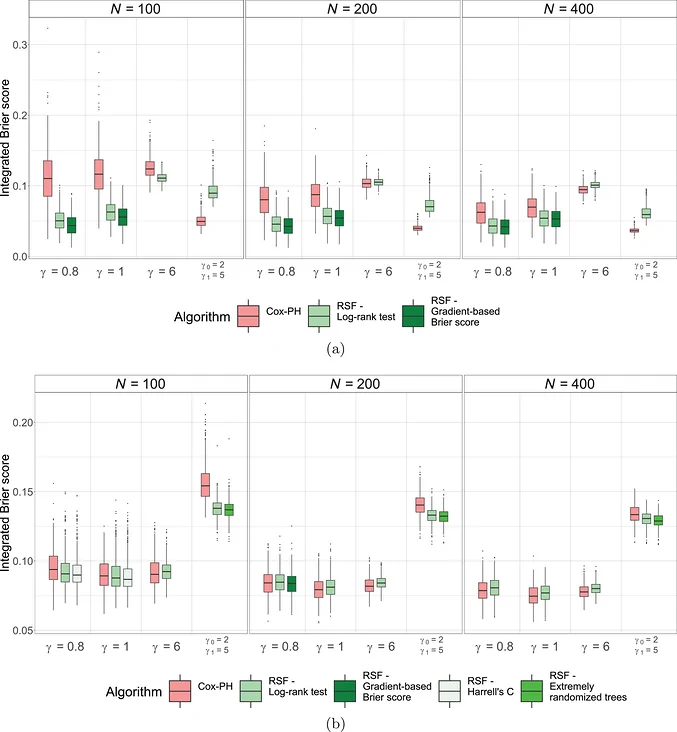
IBS estimates in the simulation scenario assuming 60% of censoring and a treatment effect of $\beta_{\text{treatment}}=-0.4$ for the RCT in PBC (a) and in prostate cancer patients (b). Survival times are generated from a Weibull distribution with scale parameters estimated from the respective reference dataset, shape parameters (γ) vary in order to examine the impact of differing hazards (γ = 0.8: decreasing hazard, γ = 1: constant hazard, γ = 6: increasing hazard, {$\gamma_{0}$ = 2, $\gamma_{1}$ = 5}: nonproportional hazards), and the violation of the proportional hazards assumption. Results are shown for different total sample sizes N. Abbreviations: Cox‐PH, Cox proportional hazards model; RSF, random survival forest.

The IBS estimates, which correspond to the models' overall performance (encompassing both the models' discrimination and calibration), are shown in Figure 5 (30% censoring rate) and Figure 6 (60% censoring rate). Results for the RCT data without treatment–covariate interactions (PBC dataset) are shown in Figures 5a and Figure 6a. The Cox model has clearly the best overall performance in the nonproportional hazards settings. It also (very slightly) outperforms the RSF in the scenario with increasing hazard when either N=400 for a censoring rate of 30% or when N∈{200,400} for a censoring rate of 60%. Otherwise, the RSF performs better. These differences are even more evident with decreasing total sample size. For decreasing and constant hazards, the “Gradient‐based Brier score” splitting rule slightly outperforms the “log‐rank test” splitting rule for the RSF for both censoring rates and all sample sizes. Results for the RCT data with multiple treatment–covariate interactions (prostate cancer dataset) are shown in Figures 5b and 6b. For a censoring proportion of 30%, the Cox model performs slightly better for the proportional hazards settings in case γ=0.8 or γ=1. In the case of increasing hazards (γ=6) or nonproportional hazards (γ∈{2,5}), the RSF clearly performs better. Alternatives to the standard “log‐rank test” splitting rule perform only slightly better, and depending on the scenario and sample size, these are differing alternatives. For a censoring rate of 60%, the Cox model performs better in some cases, especially with increasing sample size. It slightly outperforms the RSF in the case of N=100 when assuming an increasing hazard (γ=6). In the case of N=200, it additionally outperforms the RSF when assuming a constant hazard (γ=1), and in the case of N=400, it outperforms the RSF in all proportional hazards settings (γ=0.8,γ=1,γ=6). For nonproportional hazards, the RSF based on the “extremely randomized tree” splitting rule clearly performs best. For the remaining scenarios, either the RSF using the “gradient‐based Brier score” (N=200 and γ=0.8) or “Harrell's C” splitting rule (N=100 with γ=0.8, or γ=1) performs best. One observation is, that the Cox model has the better overall performance measured by the IBS for the nonproportional hazards setting in the absence of treatment–covariate interactions, but performs worse in comparison to the RSF if treatment covariate interactions are present in the data, similar to the scenario with a censoring rate of 30%.

Some calibration curves at median survival time for the RCT data without treatment–covariate interactions (PBC dataset) are shown in Figures C.5a and C.5b of Supporting Information Section C. Calibration curves for a proportional and nonproportional hazards setting are compared. Calibration curves for the respective scenarios for the RCT data with multiple treatment–covariate interactions (prostate cancer dataset) are shown in Figures C.6a and C.6b of Supporting Information Section C.3.1. Additionally, to the results of the Cox model and the RSF model based on the standard splitting rule, they show the results for those algorithms that outperformed these two approaches with respect to overall performance in the respective scenario. Calibration of the Cox model improves with increasing sample size, while for the RSF this is at least less evident. Especially, in the nonproportional hazards setting and the absence of treatment–covariate interactions, the Cox model's results are better calibrated compared to the RSF (Figure C.5b of Supporting Information Section C.3.1). In contrast, the difference in calibration between the two models is less obvious for the nonproportional hazards setting in case treatment–covariate interactions are present in the data (Figure C.6b of Supporting Information Section C.3.1). Judged by the percentiles shown as dashed lines, calibration generally varies less in the Cox model results than in the RSF results. Deviation from perfect calibration of the RSF results is sometimes caused by a too narrow range of predictions compared to the true values, resulting in calibration curves that are too steep, most notably in the nonproportional hazards settings without treatment–covariate interactions in the data (Figure C.5b of Supporting Information Section C.3.1).

**FIGURE 7:**
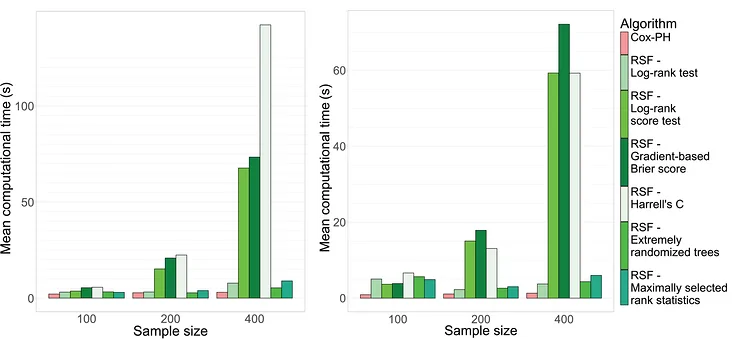
Mean computational times for the RCT data without treatment–covariate interactions (PBC dataset, left), and for the RCT data with three treatment–covariate interactions (prostate cancer dataset, right). Abbreviations: Cox‐PH, Cox proportional hazards model; RSF, random survival forest.

Computational complexity of the methods is compared in Figure 7. It includes the variable selection step for the Cox model and the grid search for finding the optimal combination of hyperparameters for the RSF. Computational times are the lowest for the Cox model, although the RSF still has relatively low computational times for total sample sizes of N=100, and even for the larger sample sizes when the RSF splitting rules “log‐rank test,”“extremely randomized trees,” or “maximally selected rank statistics” are used. In contrast, computational time considerably increases for larger sample sizes as well as a larger number of covariates for the RSF splitting rules “log‐rank score test,” “gradient‐based Brier score,” and “Harrell's C.”

Complete simulation study results can be found in Supporting Information Sections C.1 (C index), C.2 (IBS), and C.3.2 (calibration curves).

## 4 Discussion and Conclusions

An extensive neutral simulation study was performed in order to compare the performance of the Cox regression model and the RSF model for predicting survival probabilities in RCT data. For this, we followed recommendations for neutral comparison studies (Morris et al. 2019; Weber et al. 2019) to ensure an objective evaluation of the results.

A variety of settings were considered using two publicly available RCT datasets as a reference. One dataset is characterized by the absence of treatment–covariate interactions (Therneau and Lumley 2024, biliary cirrhosis dataset) and the other by two significant and one weak treatment–covariate interaction (Byar and Green 1980, prostate cancer dataset). In each case, different total sample sizes, values of the treatment effect, censoring rates, and properties of the hazard were considered for data simulation, which may occur in other real‐world datasets. Comparisons are based on measures of discrimination, calibration, and overall performance as recommended in the literature (McLernon et al. 2023; Moons et al. 2015; Steyerberg et al. 2010).

Depending on the research question, different aspects of the algorithm's performance may be more important. In previous studies comparing the Cox and RSF models in real‐world observational data, conclusions are usually based on the C index, a measure of discrimination, but its extension and application to time‐to‐event medical data has been criticized (Cook 2007; Hartman et al. 2023; Vickers and Cronin 2010). Similar to the findings of previous studies, in our simulation study, the RSF predictions were usually more accurate with respect to the C index, with some exceptions for the data with higher (60%) censoring. In the case of these higher censoring rates, the Cox model performed better in the nonproportional hazards setting in the absence of treatment–covariate interactions, and in the case of multiple treatment–covariate interactions for constant and decreasing hazards with larger sample sizes (N=200,N=400).

With respect to overall performance measured by the IBS, the Cox model performs considerably better in the nonproportional hazards setting for both censoring rates (30%, 60%) in the data without treatment–covariate interactions, but in the presence of treatment–covariate interactions, the RSF performed better for nonproportional hazards. It may be concluded that the overall performance of the Cox model is only affected by deviations from the proportional hazards assumption in the presence of treatment–covariate interactions. Overall performance of the Cox model improves more visibly with increasing sample size, while RSF results are more stable across different sample sizes, maybe due to its ability for good performance even in high‐dimensional settings.

With respect to calibration, a measure of agreement between (estimated) true and predicted outcomes, results for the RSF are worse than those for the Cox model in many cases with considerable differences. Considering overall performance, the Cox model may outperform the RSF model despite poor performance with respect to the C index, due to its better calibration. It is unclear whether results may be influenced by the approach for approximating the true outcomes when estimating the calibration, which is based on Cox model predictions.

In summary, overall performance measures such as the IBS may be more suitable for drawing general conclusions about the superiority of one method over the other for predictions in time‐to‐event data from RCTs. Findings suggest a poor performance of the Cox model when considering the C index, a conclusion that is less obvious or even reversed when considering the IBS.

All currently available splitting rules for the RSF implemented in two widely used R packages, randomForestSRC (Ishwaran et al. 2021) and ranger (Wright et al. 2023), were included. Considering the C index estimates, the “extremely randomized trees” splitting rule most often performed better than the standard “log‐rank test” RSF in the presence of treatment–covariate interactions. Considering the IBS estimates, the same applies. In the absence of treatment–covariate interactions, the “gradient‐based Brier score” splitting rule performed better than the standard RSF in scenarios with decreasing or constant hazards. Thus, it may be worthwhile to try alternative RSF splitting rules besides the default.

Additionally, computational times of some RSF splitting rules, such as the standard “log‐rank test” or the “extremely randomized trees” splitting rule, do not extremely exceed those of the Cox model for sample sizes typically expected in RCT data, in contrast to the computational time required by the RSF using other splitting rules.

Results are only affected to a minor degree by the size of the treatment effect.

Limitations of this simulation study are that only a limited number of datasets and scenarios, as well as a limited number of performance measures, can be considered. Moreover, only the combination of Weibull distributed survival times and uniformly distributed censoring times was considered. There also exist further RSF splitting rules (Ishwaran et al. 2008) that are not currently implemented in the R packages randomForestSRC (Ishwaran et al. 2021) and ranger (Wright et al. 2023), so they were not included in the method comparison.

## Funding

The authors are grateful for financial support from the Young Researchers Travel Scholarship Program of the University of Augsburg and for the financial support of the International Dimension of ERASMUS+ during Ricarda Graf's research visit to the University of Reading. The sponsors had no role in study design, collection, analysis, and interpretation of data, writing of the report, and decision to submit the article for publication.

## Conflicts of Interest

The authors declare no conflicts of interest.

## Open Research Badges

This article has earned an Open Data badge for making publicly available the digitally‐shareable data necessary to reproduce the reported results. The data is available in the Supporting Information section.

This article has earned an open data badge “**Reproducible Research**” for making publicly available the code necessary to reproduce the reported results. The results reported in this article could fully be reproduced.

## Supporting information


**Supporting File 1:** bimj70171‐sup‐0001‐SuppMat.pdf.

**Supporting File 2:** bimj70171‐sup‐0002‐Data.zip.

## Data Availability

The two datasets used as references for data simulations are publicly available: the RCT in primary biliary cirrhosis patients is available from a number of sources, for example from the Vanderbilt Department of Biostatistics (Vanderbilt Department of Biostatistics 2023), from the book by Fleming and Harrington (2005), from Kaggle (fedesoriano 1980), and in the R package survival (Therneau and Lumley 2024), and the RCT in prostate cancer patients is available in the R package subtee (Ballarini et al. 2021). The R code for reproducing the results of the simulation study is available as a supplement to this paper.
