# Supplementary material for: Comparison of the Cox Proportional Hazards Model and Random Survival Forest Algorithm for Predicting Patient‐Specific Survival Probabilities in Clinical Trial Data
Source: Biom J. 2026 Sep 25;68(5):e70171. doi: 10.1002/bimj.70171 (PMC13615326; doi:10.1002/bimj.70171)
Supplement: Supplementary file 1 [file BIMJ-68-e70171-s001.pdf]

Supplementary Material: Comparison of the Cox proportional hazards model and Random Survival Forest algorithm for predicting patient-specific survival probabilities in clinical trial data

Keywords: Cox regression, Random survival forest, Randomized controlled trials, Simulation study

A: Additional information on the bootstrap and simulation study approach

Model building in the bootstrap and simulated data

For both scenarios, the scenario without treatment-covariate interactions (primary biliary cirrhosis dataset) and the scenario with multiple treatment-covariate interactions (prostate cancer dataset) all variables (main effects) are included in the Cox regression model as well as the Random Survival forest model. Interaction terms are not included as part of the evaluation as opposed to the initial data generation. The reason is that in real-world datasets of similar sample sizes, which are typical for clinical trial settings, it is usually impossible to conclude whether interactions or nonlinear effects are present by testing their statistical significance alone due to the limited sample size (Efthimiou et al., 2024). For the Cox model, backward variable selection is done based on the Akaike information criterion (as implemented in the `R` function `stepAIC` from the `MASS` package (Ripley et al., 2024)) in order to determine which main effects will be included. The RSF does intrinsic variable selection: for each split in the tree a random subset of splitting variables among the available variables is selected, and data are partitioned based on the splitting value which yields the optimal result with respect to the splitting rule, i.e. which results in highest heterogeneity between the two respective subsets.
Application of the RSF requires specification of some hyperparameter values, which can be done using grid search. This means that multiple values around the recommended defaults are specified, and for each combination the error rate ($1 - C$ index) is computed in those

data which were not used to build a particular tree model, i.e. the “out-of-bag” data. The RSF does work reasonably well even without extensive hyperparameter tuning (Boehmke and Greenwell, 2019). According to Boehmke and Greenwell (2019), the number of trees and the number of randomly chosen variables considered for data splitting in a node (m_{try}) are the most important hyperparameters. They recommend to use 10 times the number of variables (covariates) present in the model as the number of trees. They mention the default which equals the square root of the number of covariates rounded down to the nearest integer ($m_{\text{try}} = \lfloor \sqrt{\#\text{covariates}} \rfloor$) for the number of randomly chosen splitting variables per node, and which is used in both `R` packages, `randomForestSRC` (Ishwaran and Kogalur, 2023) and `ranger` (Wright et al., 2023). Boehmke and Greenwell (2019) recommend to consider five evenly spaced values around this default during grid search. In order to prevent overfitting, early-stopping criteria can be used with both `R` functions, either the maximum tree depth or a minimum number of samples per terminal/leaf node can be specified. In `randomForestSRC` (Ishwaran and Kogalur, 2023) the default for the minimum number of samples in a terminal node is set to 15 observations. For grid search, five, ten and 15 times the number of covariates for the number of trees were considered, 15, 30, and 45 for the number of observations in terminal nodes, and three, four, and five for the value of m_{try}. Less than the recommended five values for m_{try} were chosen because these RCT datasets consist of a rather small number of covariates compared to other applications of the RSF.

Random survival forest (RSF) splitting rules

The following table contains information on the currently available RSF splitting rules that were compared in the simulation study.

Table A.1: Overview of currently available splitting rules for the RSF in the `R` packages `randomForestSRC` (Ishwaran et al., 2008) and `ranger` (Wright et al., 2023) compared in the simulation study.

Splitting rule	Notes	Recommendations ("+")/drawbacks ("−")	Abbreviation in `R` package	Availability
Log-rank test	• standard split criterion, most widely used	+ log-rank and log-rank score splitting almost always have the lowest prediction error but poor performance in data with high censoring rates<br>+ stable/preferable in noisy scenarios (Ishwaran et al., 2008; Schmid et al., 2016)<br>−biased, i.e. favors splitting variables with many possible splits (Wright et al., 2016)<br>−preference for more unbalanced splits ("end-cut preference")(Schmid et al., 2016)<br>−less suitable for small-scale clinical trials and high censoring rates (Schmid et al., 2016)	logrank	`randomForestSRC`<br>`ranger`
Log-rank score (Hothorn and Lausen, 2003)	• a standardized log-rank statistic	+ log-rank and log-rank score splitting almost always have the lowest prediction error but poor performance in data with high censoring rates, stable in presence of noise variables (Ishwaran et al., 2008)	logrankscore	`randomForestSRC`
Gradient-based (global non-quantile) Brier score		+ preferable to log-rank splitting when censoring depends strongly on the covariates (more robust) (Ishwaran and Lu, 2019)	bs.gradient	`randomForestSRC`
Harrell's C (Schmid et al., 2016)	• Harrell's C (C index) most common performance measure for RSF → overcomes discrepancy between node splitting and model evaluation	+ recommend it for smaller scale clinical trials and if censoring is high<br>+ has a lower end-cut preference than the log-rank statistic<br>−recommend log-rank test for large-scale (omics) studies (Schmid et al., 2016)	C	`ranger`
Extremely randomized trees (Geurts et al., 2006)	• strong randomization of variable and cut-point choices while splitting	+ main strength: computational efficiency (Geurts et al., 2006)	extratrees	`ranger`
Maximally selected rank statistics (Wright et al., 2016)	• splitting variables are compared on the p-value scale	+ unbiased in case of informative dichotomous and uninformative categorical variables with more possible splits	maxstat	`ranger`

B: Parameters used for data simulation

In this section, details regarding data simulations from copula models are given, i.e. correlation matrices and parametric distributions. Comparisons of the true covariate distributions and the fitted distributions are also shown.

Table B.1a: Correlation matrix of the variables in the primary biliary cirrhosis dataset used in the copula model.

	Age	Sex	Presence of ascites	Presence of hepatomegaly	Presence of spiders	Presence of edema	Serum bilirubin	Serum cholesterol	Albumin	Urine copper	Alkaline phosphatase	SGOT	Triglycerides	Platelet count	Prothrombin time	Histologic stage of disease
Age	1	-0.1931	0.216	0.0862	-0.0529	0.1966	0.0386	-0.1493	-0.1953	0.0613	-0.0472	-0.1499	0.0209	-0.1384	0.1963	0.1297
Sex	-0.1931	1	0.0087	0.0509	0.14	0.0371	-0.0385	0.0053	-0.0653	-0.2301	0.0043	-0.0184	-0.0874	0.0855	-0.1211	-0.0268
Presence of ascites	0.216	0.0087	1	0.159	0.1593	0.4257	0.3756	-0.1071	-0.3574	0.218	0.0326	0.0887	0.1348	-0.2184	0.3214	0.3683
Presence of hepato-megaly	0.0862	0.0509	0.159	1	0.283	0.1714	0.4109	0.0957	-0.3083	0.283	0.1686	0.1621	0.1564	-0.2062	0.2144	0.4953
Presence of spiders	-0.0529	0.14	0.1593	0.283	1	0.2427	0.3673	-0.006	-0.2348	0.2704	0.0948	0.1565	0.0091	-0.1588	0.2566	0.3298
Presence of edema	0.1966	0.0371	0.4257	0.1714	0.2427	1	0.3863	-0.1294	-0.3237	0.2124	0.0389	0.106	0.0652	-0.2126	0.3403	0.2814
Serum bilirubin	0.0386	-0.0385	0.3756	0.4109	0.3673	0.3863	1	0.3806	-0.3346	0.4565	0.117	0.4417	0.4185	-0.0868	0.3617	0.3049
Serum cholesterol	-0.1493	0.0053	-0.1071	0.0957	-0.006	-0.1294	0.3806	1	-0.064	0.123	0.1401	0.3372	0.2768	0.178	-0.0294	-0.004
Albumin	-0.1953	-0.0653	-0.3574	-0.3083	-0.2348	-0.3237	-0.3346	-0.064	1	-0.2643	-0.1015	-0.22	-0.0947	0.203	-0.234	-0.2684
Urine copper	0.0613	-0.2301	0.218	0.283	0.2704	0.2124	0.4565	0.123	-0.2643	1	0.1873	0.2936	0.2723	-0.0641	0.2179	0.23
Alkaline phospha-tase	-0.0472	0.0043	0.0326	0.1686	0.0948	0.0389	0.117	0.1401	-0.1015	0.1873	1	0.1122	0.1687	0.1428	0.0894	0.0992
SGOT	-0.1499	-0.0184	0.0887	0.1621	0.1565	0.106	0.4417	0.3372	-0.22	0.2936	0.1122	1	0.1194	-0.1195	0.1122	0.1455
Triglycerides	0.0209	-0.0874	0.1348	0.1564	0.0091	0.0652	0.4185	0.2768	-0.0947	0.2723	0.1687	0.1194	1	0.0948	0.0192	0.0749
Platelet count	-0.1384	0.0855	-0.2184	-0.2062	-0.1588	-0.2126	-0.0868	0.178	0.203	-0.0641	0.1428	-0.1195	0.0948	1	-0.2213	-0.1984
Prothrombin time	0.1963	-0.1211	0.3214	0.2144	0.2566	0.3403	0.3617	-0.0294	-0.234	0.2179	0.0894	0.1122	0.0192	-0.2213	1	0.2572
Histologic stage of disease	0.1297	-0.0268	0.3683	0.4953	0.3298	0.2814	0.3049	-0.004	-0.2684	0.23	0.0992	0.1455	0.0749	-0.1984	0.2572	1

Table B.1b: Correlation matrix of the variables in the RCT in prostate cancer patients used in the copula model.

	Age	Standardized weight	Systolic blood pressure	Diastolic blood pressure	Size of primary tumour	Serum prostatic acid phosphatase	Haemoglobin	Gleason stage-grade	Performance status	History of cardio-vascular disease	Presence of bone metastases	Prostate cancer stage	Abnormal electrocardiogram
Age	1	-0.0761	0.0863	-0.0727	0.0137	-0.0628	-0.0842	-0.0518	0.0529	0.1699	-0.0717	-0.0125	0.1658
Standardized weight	-0.0761	1	0.1911	0.2265	-0.0440	-0.0654	0.2612	-0.0864	-0.0940	0.0558	-0.1827	-0.0792	0.0148
Systolic blood pressure	0.0863	0.1911	1	0.6289	0.0451	-0.0532	0.0608	-0.0283	0.0538	0.1231	-0.0424	-0.0067	0.1080
Diastolic blood pressure	-0.0727	0.2265	0.6289	1	-0.0474	-0.0605	0.1437	-0.0731	-0.0225	0.0362	-0.0825	-0.0227	0.0775
Size of primary tumour	0.0137	-0.0440	0.0451	-0.0474	1	0.0895	-0.1375	0.3748	0.0704	-0.0876	0.2538	0.2190	0.0183
Serum prostatic acid phosphatase	-0.0628	-0.0654	-0.0532	-0.0605	0.0895	1	-0.1355	0.1531	0.0999	-0.0335	0.3061	0.6594	-0.0122
Haemoglobin	-0.0842	0.2612	0.0608	0.1437	-0.1375	-0.1355	1	-0.1396	-0.1409	0.0108	-0.3004	-0.1241	-0.0245
Gleason stage-grade	-0.0518	-0.0864	-0.0283	-0.0731	0.3748	0.1531	-0.1396	1	0.1277	-0.1474	0.3873	0.6509	-0.0203
Performance status	0.0529	-0.0940	0.0538	-0.0225	0.0704	0.0999	-0.1409	0.1277	1	-0.0653	-0.1919	0.127	-0.0565
History of cardiovascular disease	0.1699	0.0558	0.1231	0.0362	-0.0876	-0.0335	0.0108	-0.1474	-0.0653	1	0.0576	0.0946	0.1849
Presence of bone metastases	-0.0717	-0.1827	-0.0424	-0.0825	0.2538	0.3061	-0.3004	0.3873	-0.1919	0.0576	1	-0.6679	0.0745
Prostate cancer stage	-0.0125	-0.0792	-0.0067	-0.0227	0.2190	0.6594	-0.1241	0.6509	0.127	0.0946	-0.6679	1	-0.0303
Abnormal electrocardiogram	0.1658	0.0148	0.108	0.0775	0.0183	-0.0122	-0.0245	-0.0203	-0.0565	0.1849	0.0745	-0.0303	1

Table B.2a: Univariate distributions from which covariate values are simulated (primary biliary cirrhosis dataset).

Variable	Description	Distribution
Z_2	Age *[Years]*	$Z_2 \sim \mathcal{TN}(\mu = 50.02, \sigma = 10.58, \mathrm{a} = 26.28, \mathrm{b} = 78.44)$
Z_3	Sex *[0: male, 1: female]*	$Z_3 \sim Ber(p = 0.88)$
Z_4	Presence of ascites *[0: no, 1: yes]*	$Z_4 \sim Ber(p = 0.08)$
Z_5	Presence of hepatomelagy *[0: no, 1: yes]*	$Z_5 \sim Ber(p = 0.51)$
Z_6	Presence of spiders *[0: no, 1: yes]*	$Z_6 \sim Ber(p = 0.29)$
Z_7	Presence of edema [1)]	$Z_7 \sim \mathcal{TN}(\mu = 0.11, \sigma = 0.27, \mathrm{a} = 0, \mathrm{b} = 1)$
Z_8	Serum bilirubin *[mg/dl]*	$Z_8 \sim \mathcal{TLN}(\log(\mu) = 0.58, \log(\sigma) = 1.03, \mathrm{a} = 0.3, \mathrm{b} = 28)$
Z_9	Serum cholesterol *[mg/dl]*	$Z_9 \sim \mathcal{TLN}(\log(\mu) = 5.81, \log(\sigma) = 0.42, \mathrm{a} = 120, \mathrm{b} = 1775)$
Z_{10}	Albumin *[gm/dl]*	$Z_{10} \sim \mathcal{TN}(\mu = 3.52, \sigma = 0.42, \mathrm{a} = 1.96, \mathrm{b} = 4.64)$
Z_{11}	Urine copper *[mg/day]*	$Z_{11} \sim \mathcal{TEXP}(\lambda = 0.01, \mathrm{b} = 488)$
Z_{12}	Alkaline phosphatase *[U/liter]*	$Z_{12} \sim \mathcal{TLN}(\log(\mu) = 7.27, \log(\sigma) = 0.72, \mathrm{a} = 289, \mathrm{b} = 13862.4)$
Z_{13}	Aspartate aminotransferase - SGOT *[U/ml]*	$Z_{13} \sim \mathcal{TLN}(\log(\mu) = 4.71, \log(\sigma) = 0.45, \mathrm{a} = 26.35, \mathrm{b} = 457.25)$
Z_{14}	Triglycerides *[mg/dl]*	$Z_{14} \sim \mathcal{TLN}(\log(\mu) = 4.73, \log(\sigma) = 0.43, \mathrm{a} = 33, \mathrm{b} = 598)$
Z_{15}	Platelet count *[# platelets per m^3/1000]*	$Z_{15} \sim \mathcal{TN}(\mu = 261.94, \sigma = 95.0, \mathrm{a} = 62, \mathrm{b} = 563)$
Z_{16}	Prothrombin time *[sec]*	$Z_{16} \sim \mathcal{TLN}(\log(\mu) = 2.37, \log(\sigma) = 0.09, \mathrm{a} = 9, \mathrm{b} = 17.1)$
Z_{17}	Histologic stage of disease *[grade]*	$Z_{17} \sim \mathcal{TN}(\mu = 3.03, \sigma = 0.88, \mathrm{a} = 1, \mathrm{b} = 4)$

Table B.2b: Univariate distributions from which covariate values are simulated (RCT in prostate cancer patients).

Variable	Description	Distribution
AGE	Age *[Years]*	AGE $\sim \mathcal{TN}(\mu = 71.56, \sigma = 6.9, \mathrm{a} = 48, \mathrm{b} = 89)$
WT	Standardized weight	WT $\sim \mathcal{TLN}(\log(\mu) = 4.58, \log(\sigma) = 0.13, \mathrm{a} = 69, \mathrm{b} = 152)$
SBP	Systolic blood pressure	SBP $\sim \mathcal{TLN}(\log(\mu) = 2.65, \log(\sigma) = 0.16, \mathrm{a} = 8, \mathrm{b} = 30)$
DBP	Diastolic blood pressure	DBP $\sim \mathcal{TLN}(\log(\mu) = 2.08, \log(\sigma) = 0.18, \mathrm{a} = 4, \mathrm{b} = 18)$
SZ	Size of primary tumour *[centimeters squared]*	SZ $\sim \mathcal{TEXP}(\lambda = 0.07, \mathrm{b} = 69)$
AP	Serum (prostatic) acid phosphatase *[King-Armstrong units]*	AP $\sim \mathcal{TLN}(\log(\mu) = 2.64, \log(\sigma) = 1.63, \mathrm{a} = 1, \mathrm{b} = 9999)$
HG	Haemoglobin *[g/100ml]*	HG $\sim \mathcal{TN}(\mu = 134.2, \sigma = 19.36, \mathrm{a} = 59, \mathrm{b} = 182)$
SG	Gleason stage-grade category	SG $\sim \mathcal{TN}(\mu = 10.3, \sigma = 2.02, \mathrm{a} = 5, \mathrm{b} = 15)$
PF	Performance status	PF $\sim \mathcal{B}(p = 0.1)$
HX	History of cardiovascular disease *[0: no, 1: yes]*	HX $\sim \mathcal{B}(p = 0.43)$
BM	Presence of bone metastases *[0: no, 1: yes]*	BM $\sim \mathcal{B}(p = 0.16)$
EKG	Abnormal electrocardiogram *[0: normal, 1: abnormal]*	EKG $\sim \mathcal{B}(p = 0.66)$

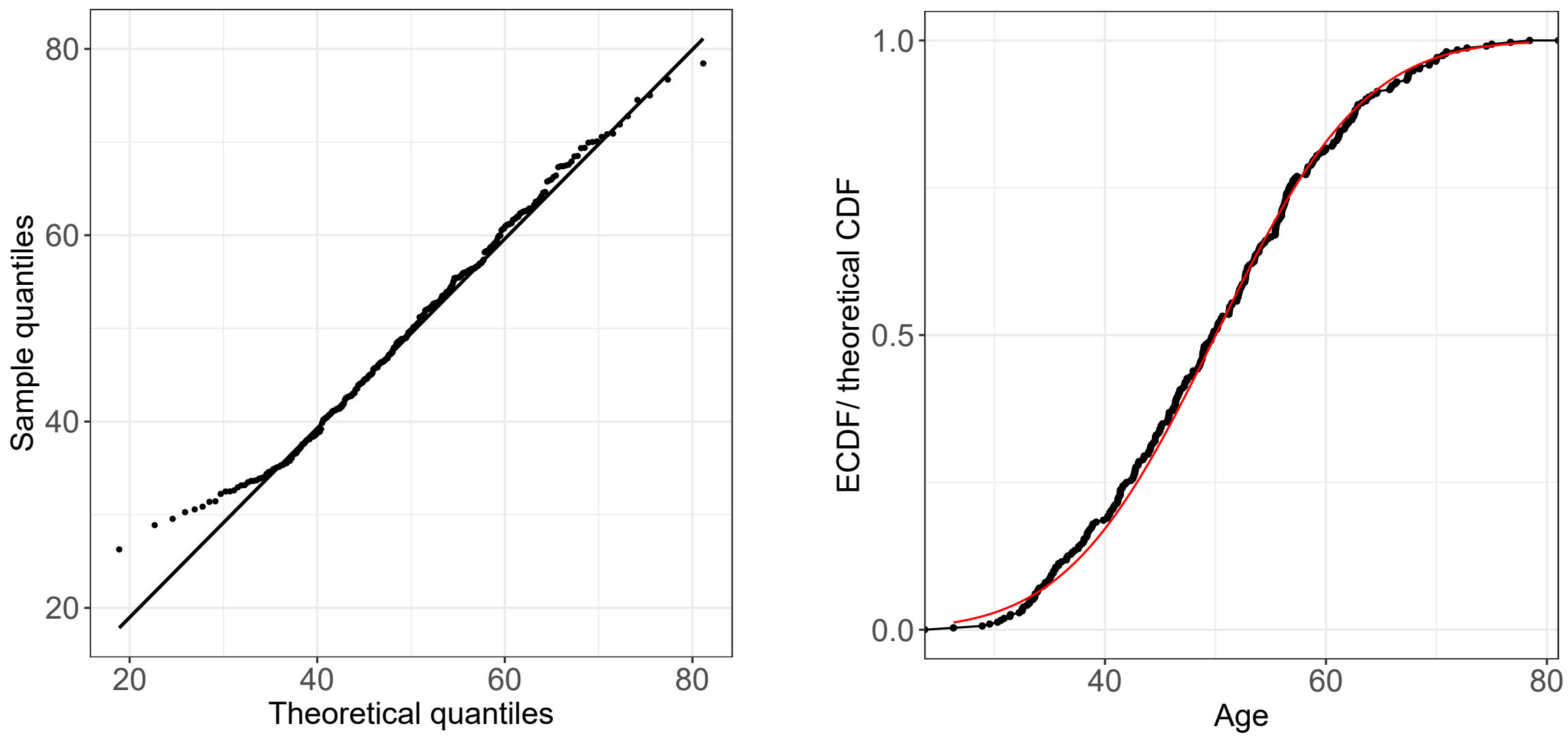

Fig. B.1a Distribution of age compared to the truncated normal distribution with $\mu = 50.02$, and $\sigma = 10.58$.

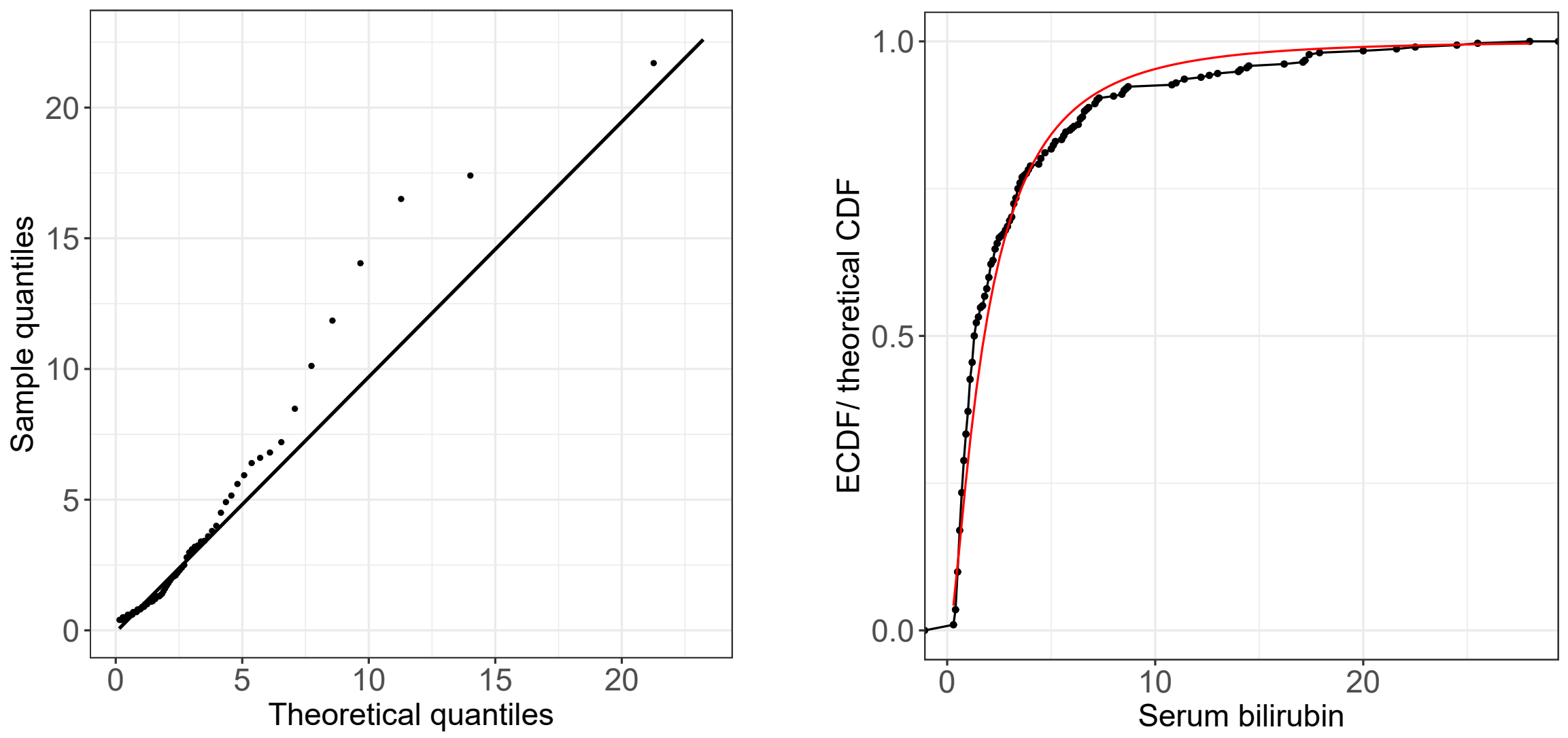

Fig. B.1b Distribution of serum bilirubin compared to the truncated log-normal distribution with $\log(\mu) = 0.58$, and $\log(\sigma) = 1.03$.

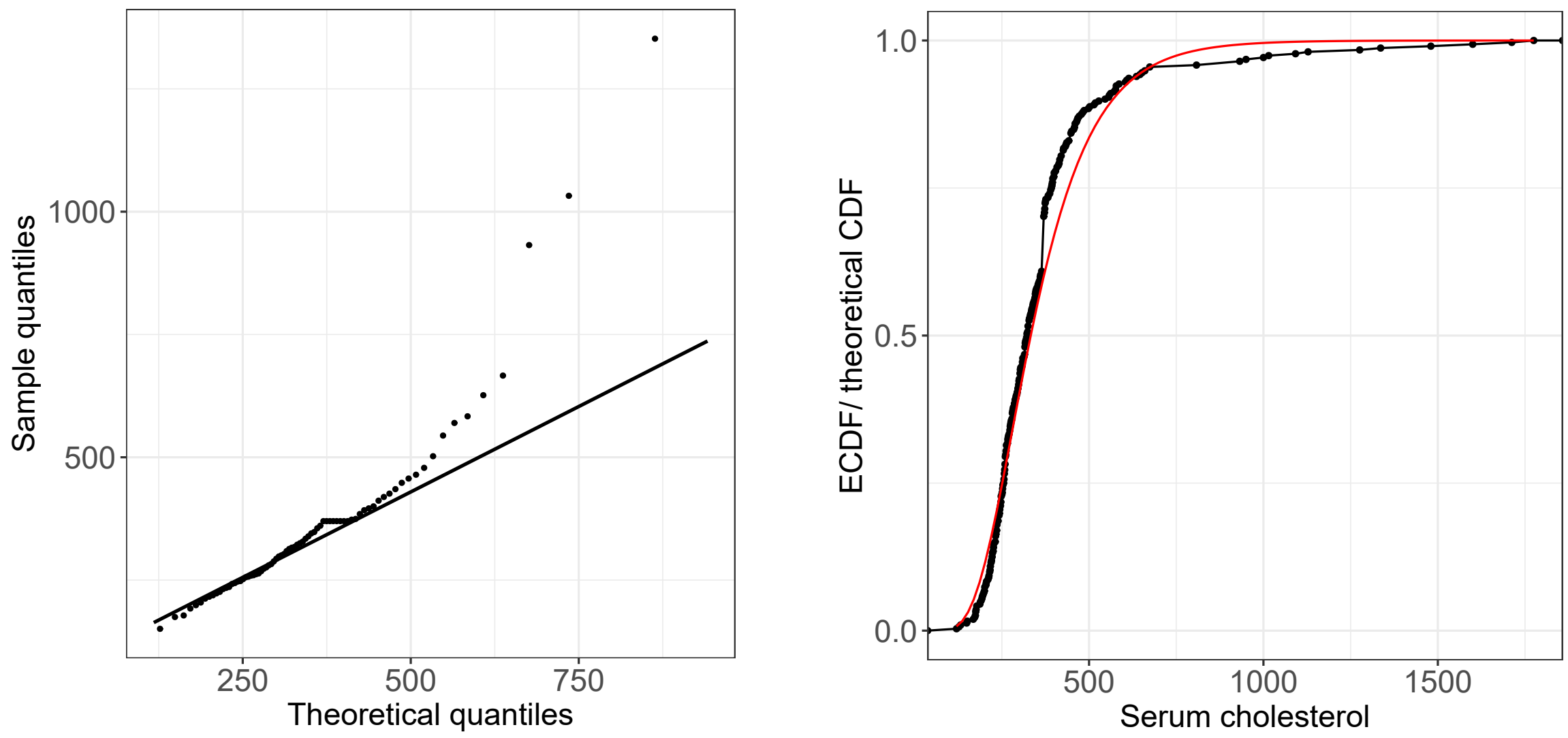

Fig. B.1c Distribution of serum cholesterol compared to the truncated log-normal distribution with $\log(\mu) = 5.81$, and $\log(\sigma) = 0.42$.

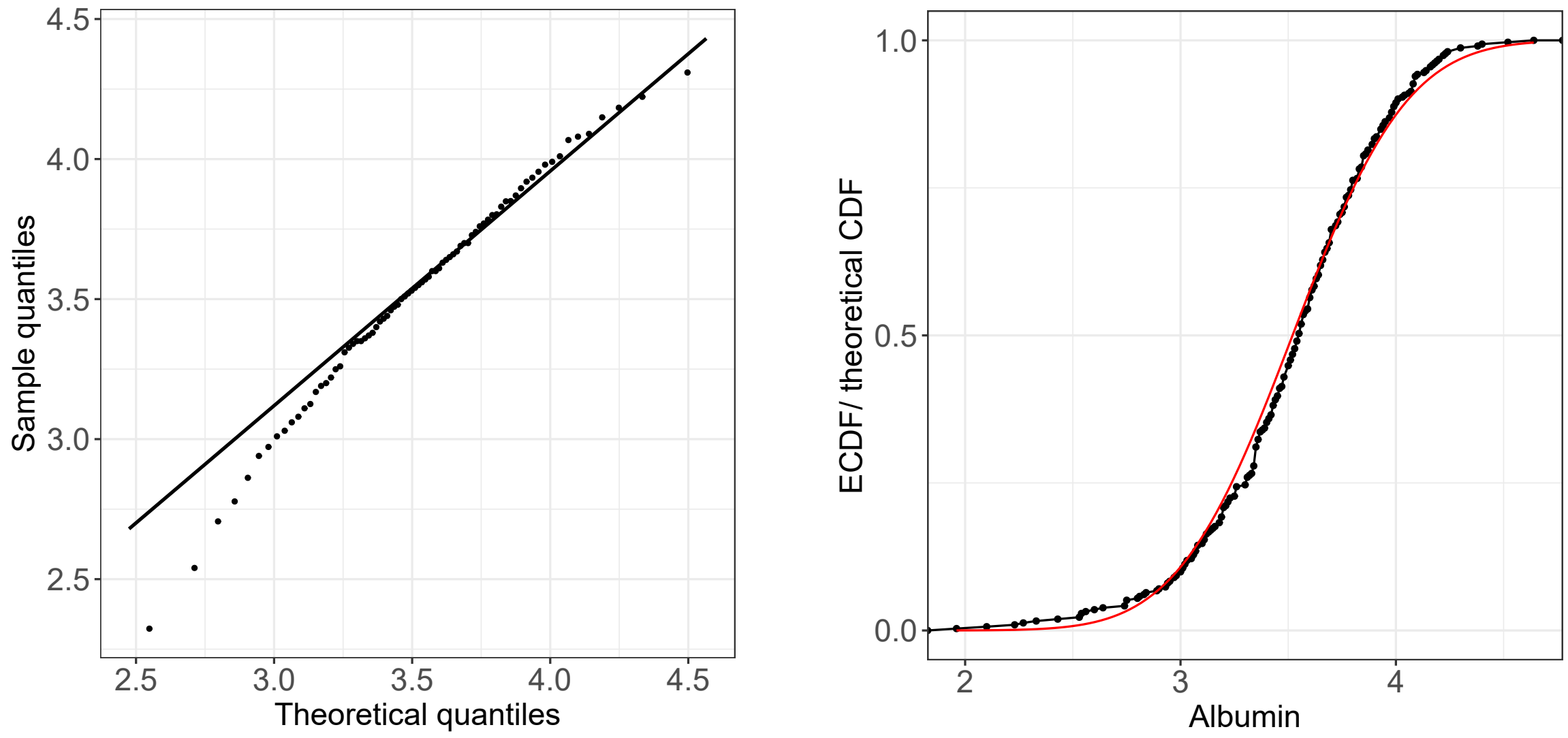

Fig. B.1d Distribution of albumin compared to the truncated normal distribution with $\mu = 3.52$, and $\sigma = 0.42$.

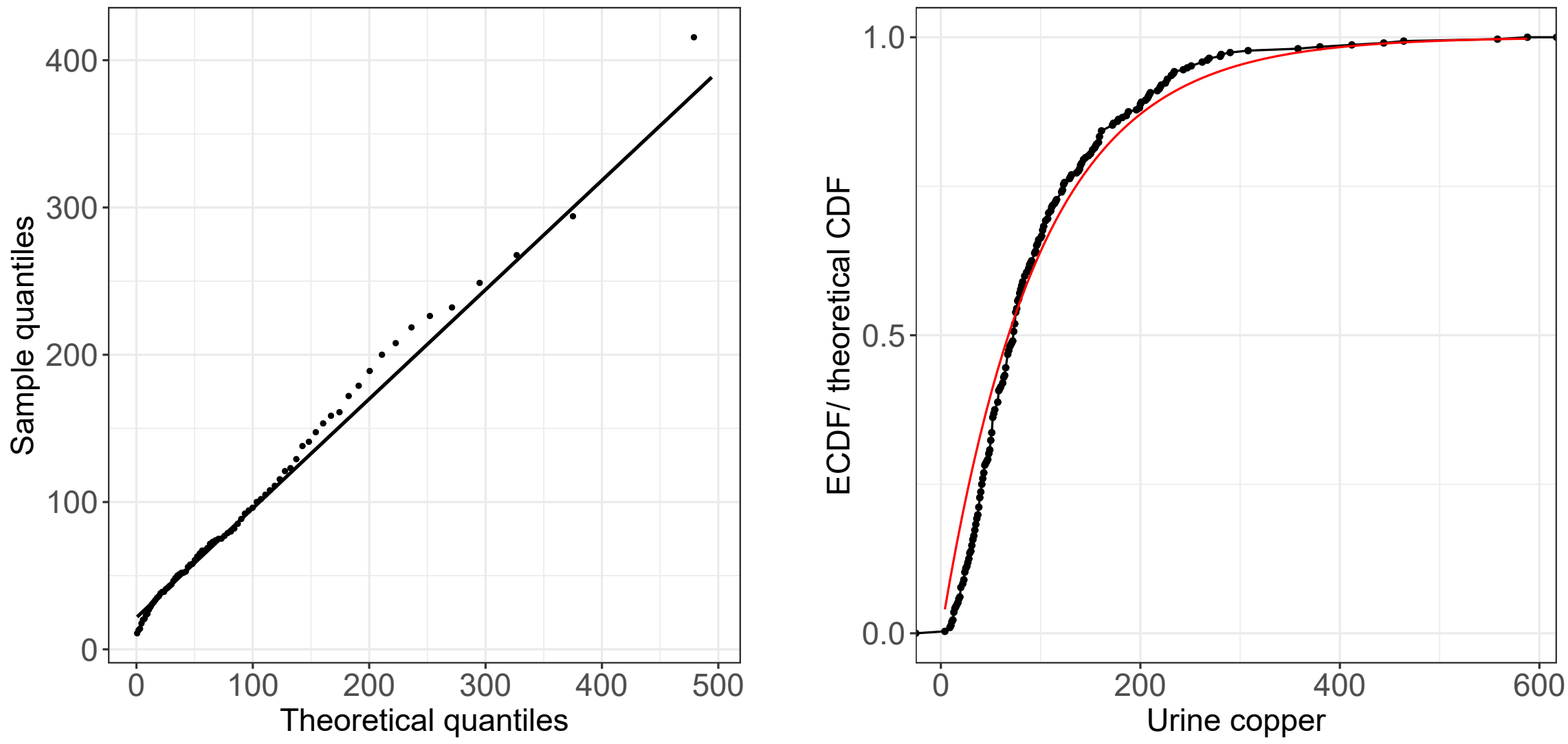

Fig. B.1e Distribution of urine copper compared to the truncated exponential distribution with $\lambda = 0.01$.

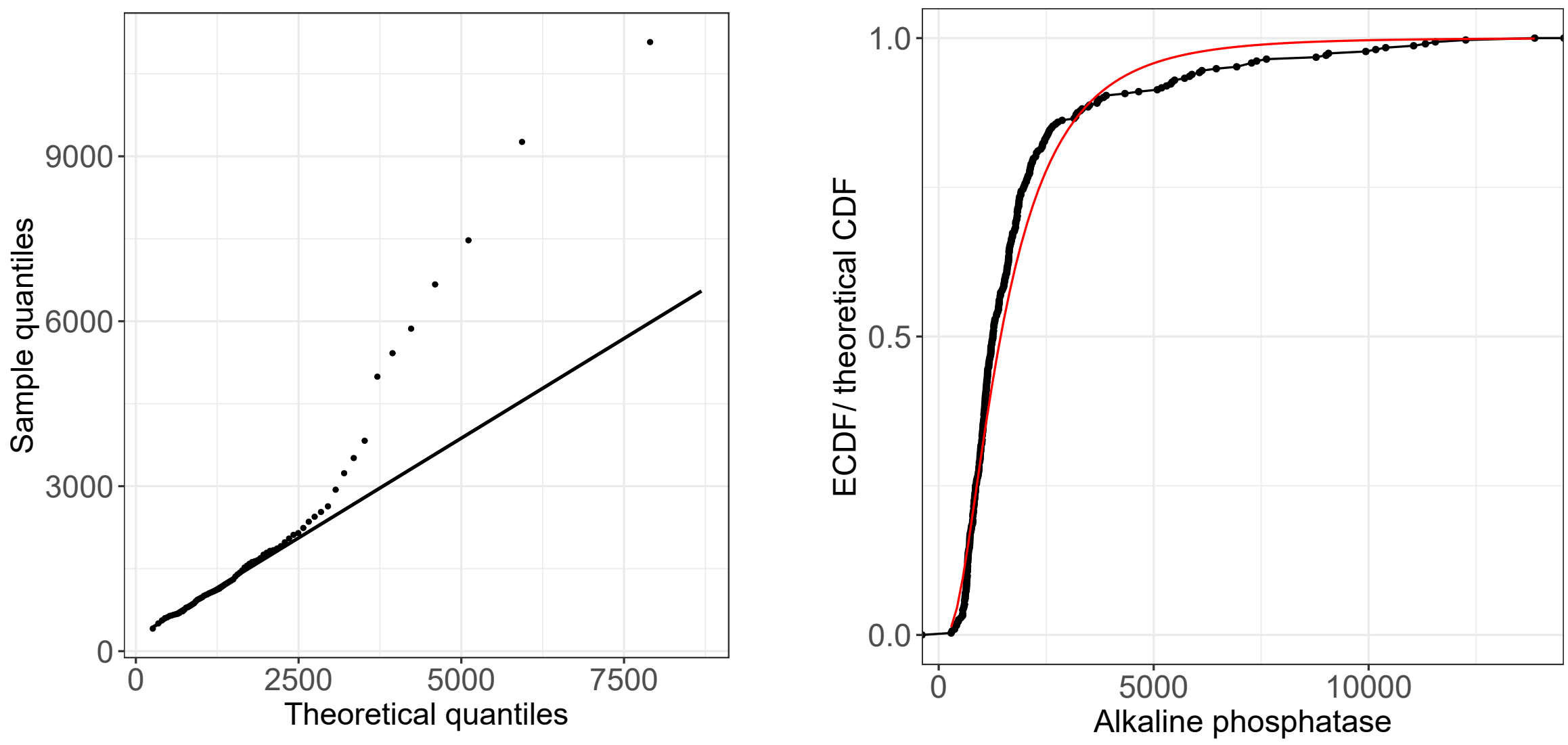

Fig. B.1f Distribution of alkaline phosphatase compared to the truncated log-normal distribution with $\log(\mu) = 7.27$, and $\log(\sigma) = 0.72$.

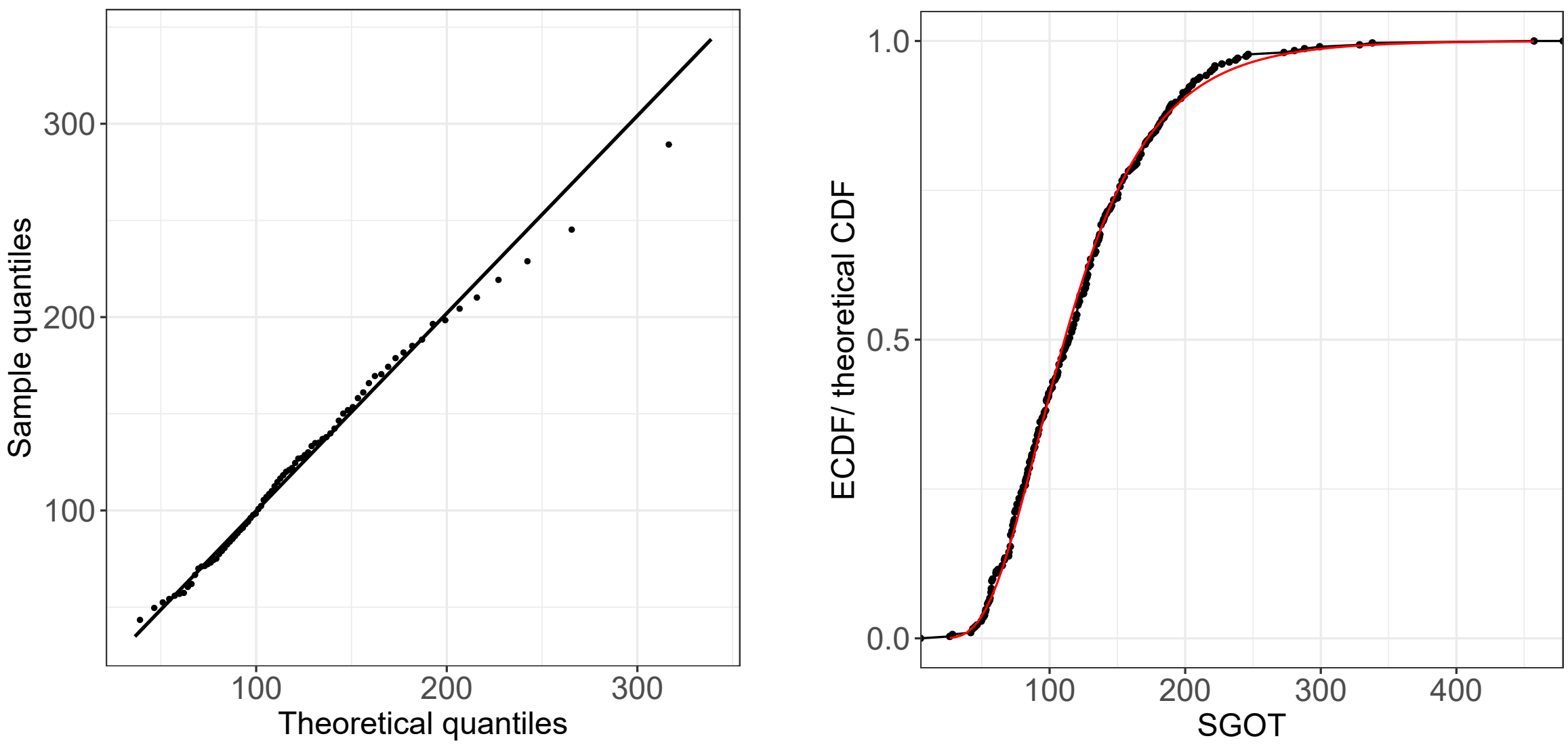

Fig. B.1g Distribution of SGOT compared to the truncated log-normal distribution with $\log(\mu) = 4.71$, and $\log(\sigma) = 0.45$.

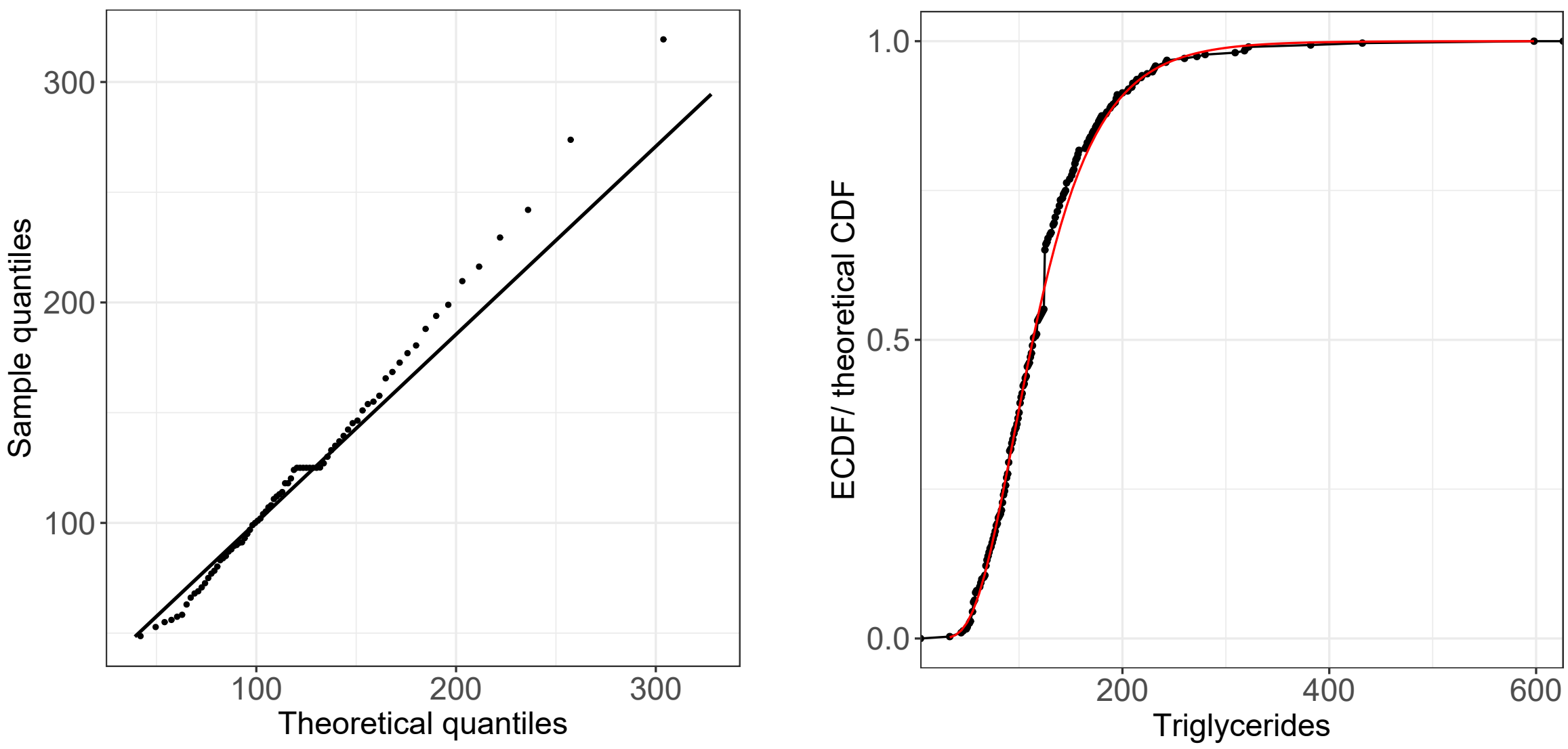

Fig. B.1h Distribution of triglycerides compared to the truncated log-normal distribution with $\log(\mu) = 4.73$, and $\log(\sigma) = 0.43$.

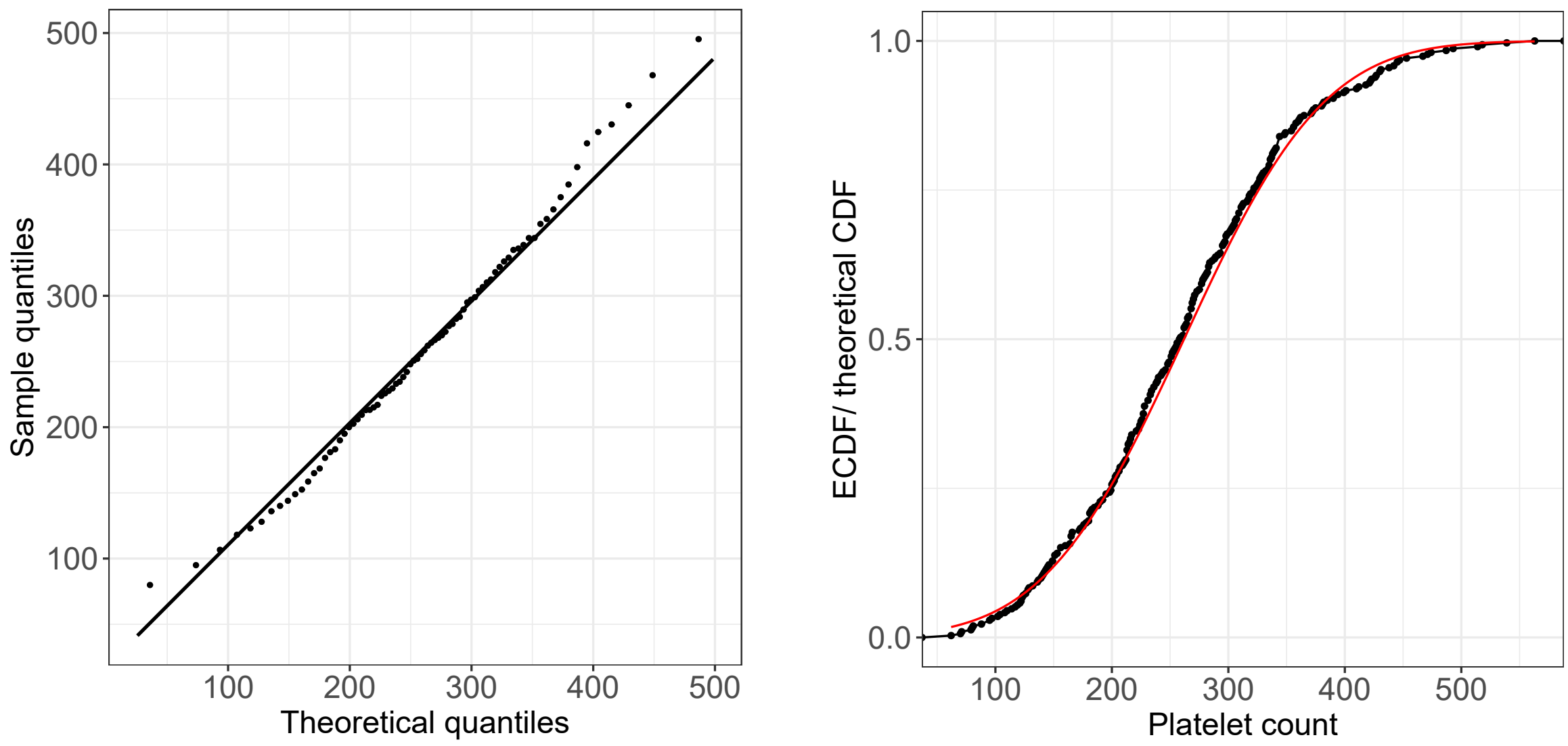

Fig. B.1i: Distribution of platelet count compared to the truncated normal distribution with $\mu = 261.94$, and $\sigma = 95.0$.

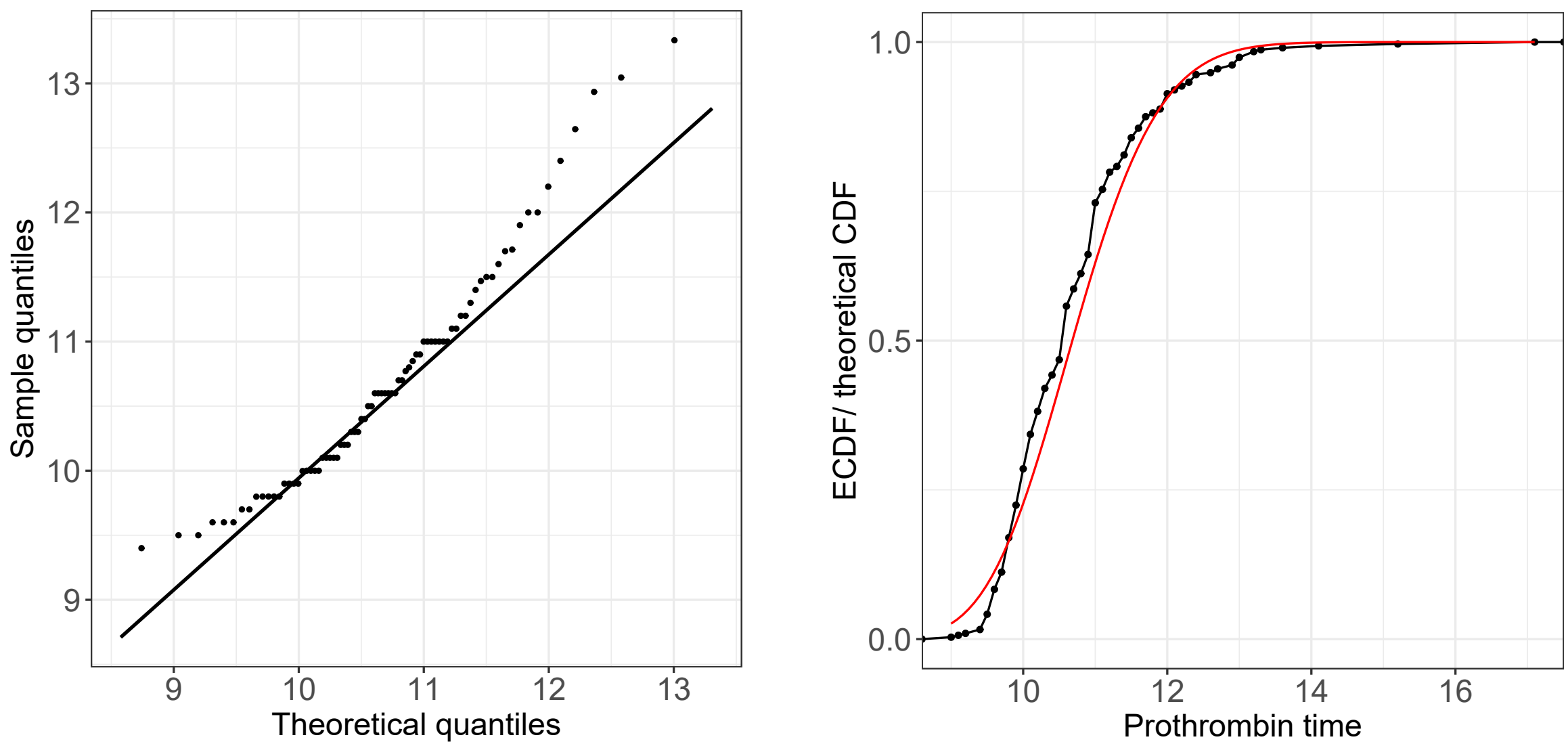

Fig. B.1j: Distribution of prothrombin time compared to the truncated log-normal distribution with $\log(\mu) = 2.37$, and $\log(\sigma) = 0.09$.

B.1: **Comparison of the true covariate distribution with the distribution used in the copula model (biliary cirrhosis dataset).** QQ-plot (left) and plot comparing the theoretical and empirical cumulative distribution functions (right) based on maximum likelihood estimates of the respective distributional parameters for each continuous variable in the primary biliary cirrhosis dataset. Truncated distributions were fitted with lower and upper limits corresponding to the minimum and maximum value of the variable.

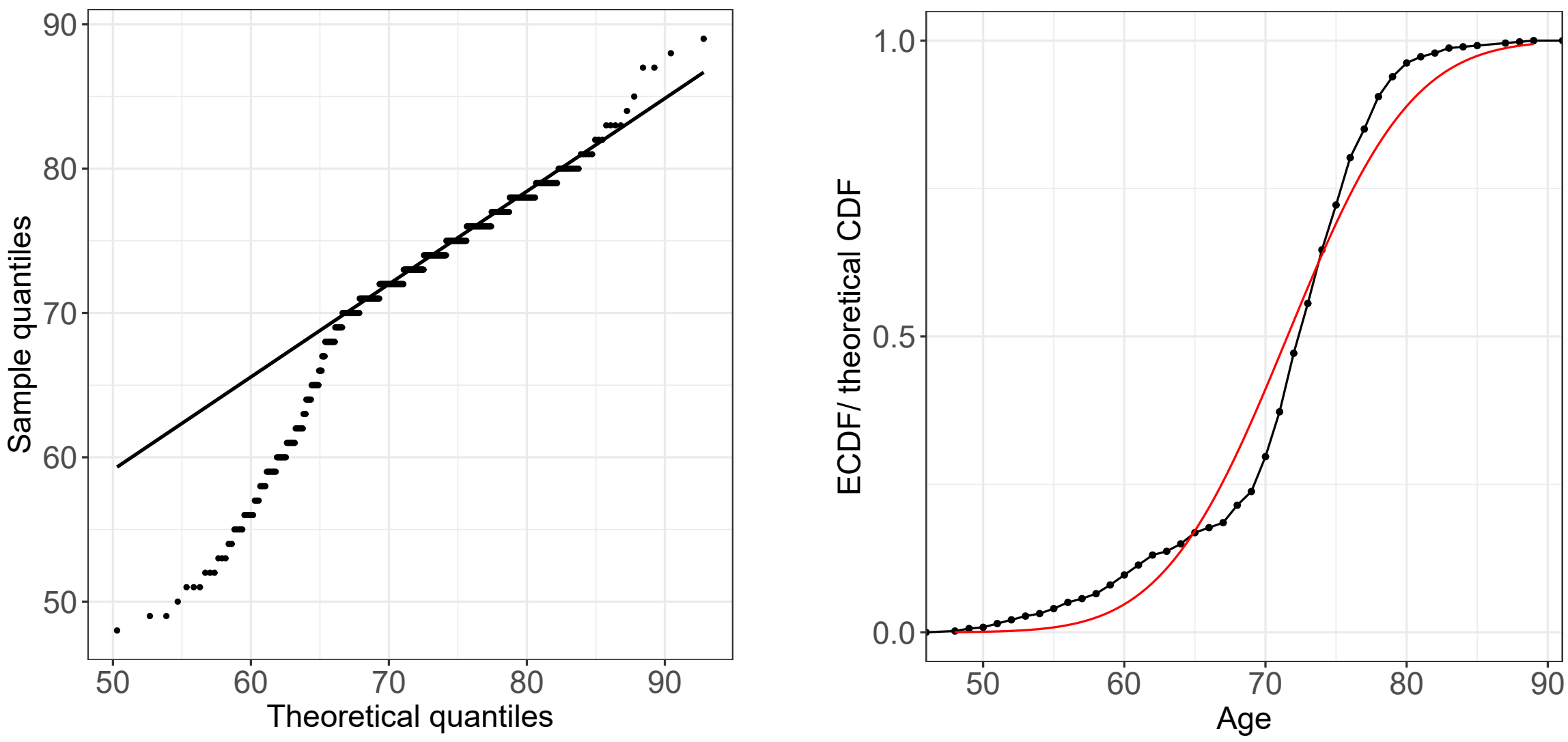

Fig. B.2a Distribution of age compared to the truncated normal distribution with $\mu = 71.56$, and $\sigma = 6.91$.

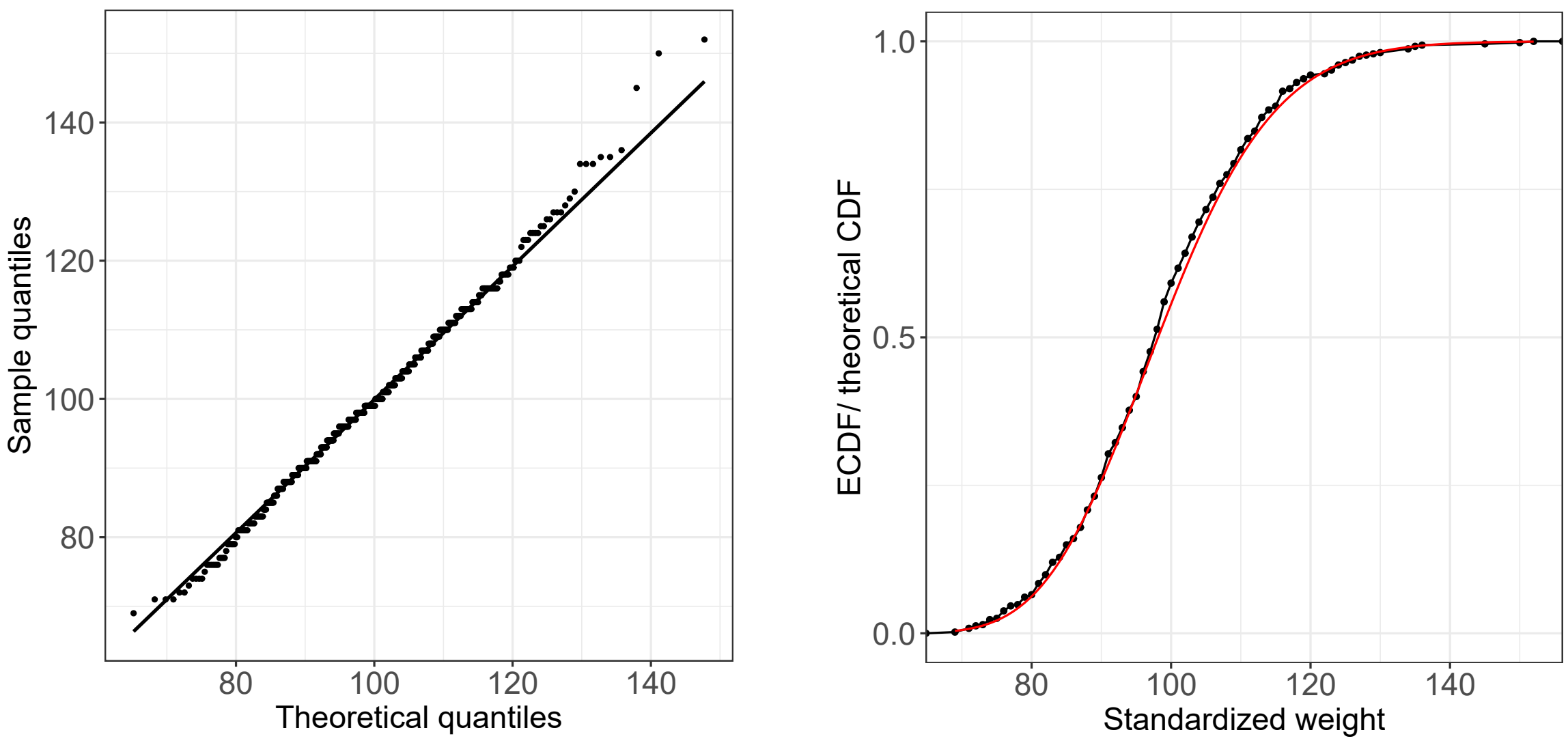

Fig. B.2b Distribution of standardized weight compared to the truncated log-normal distribution with $\log(\mu) = 4.59$, and $\log(\sigma) = 0.13$.

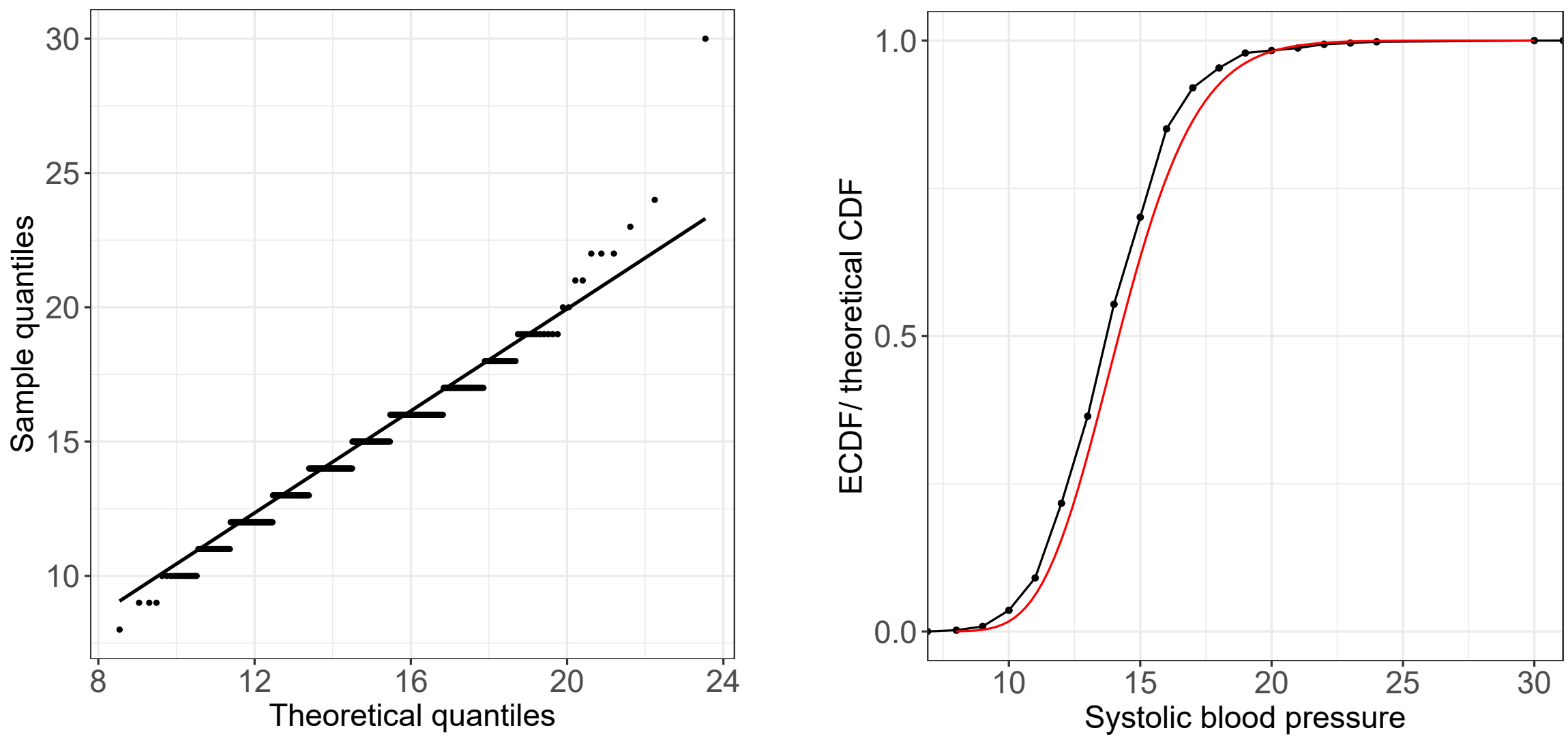

Fig. B.2c Distribution of systolic blood pressure compared to the truncated log-normal distribution with $\log(\mu) = 2.65$, and $\log(\sigma) = 0.16$.

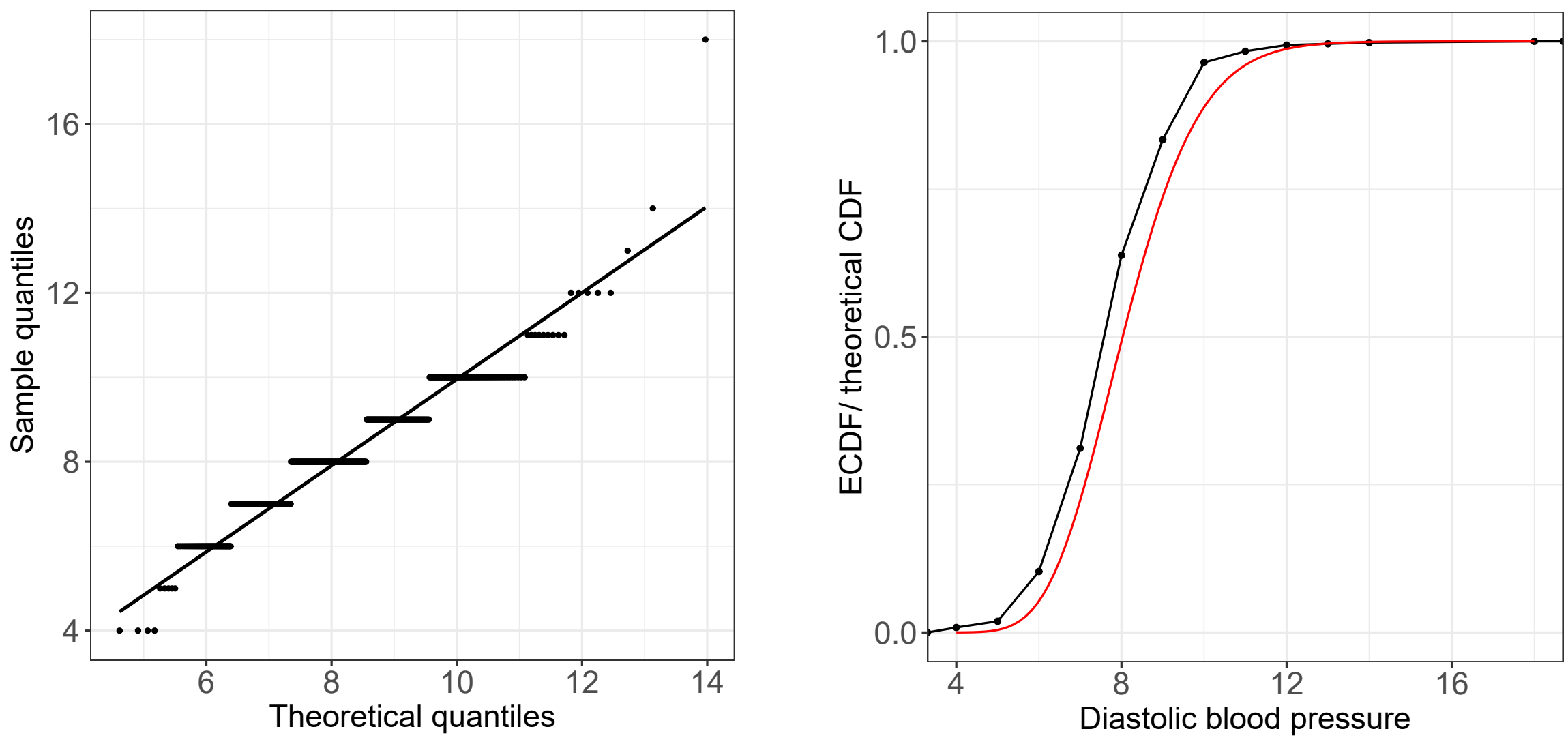

Fig. B.2d Distribution of diastolic blood pressure compared to the truncated log-normal distribution with $\log(\mu) = 2.08$, and $\log(\sigma) = 0.18$.

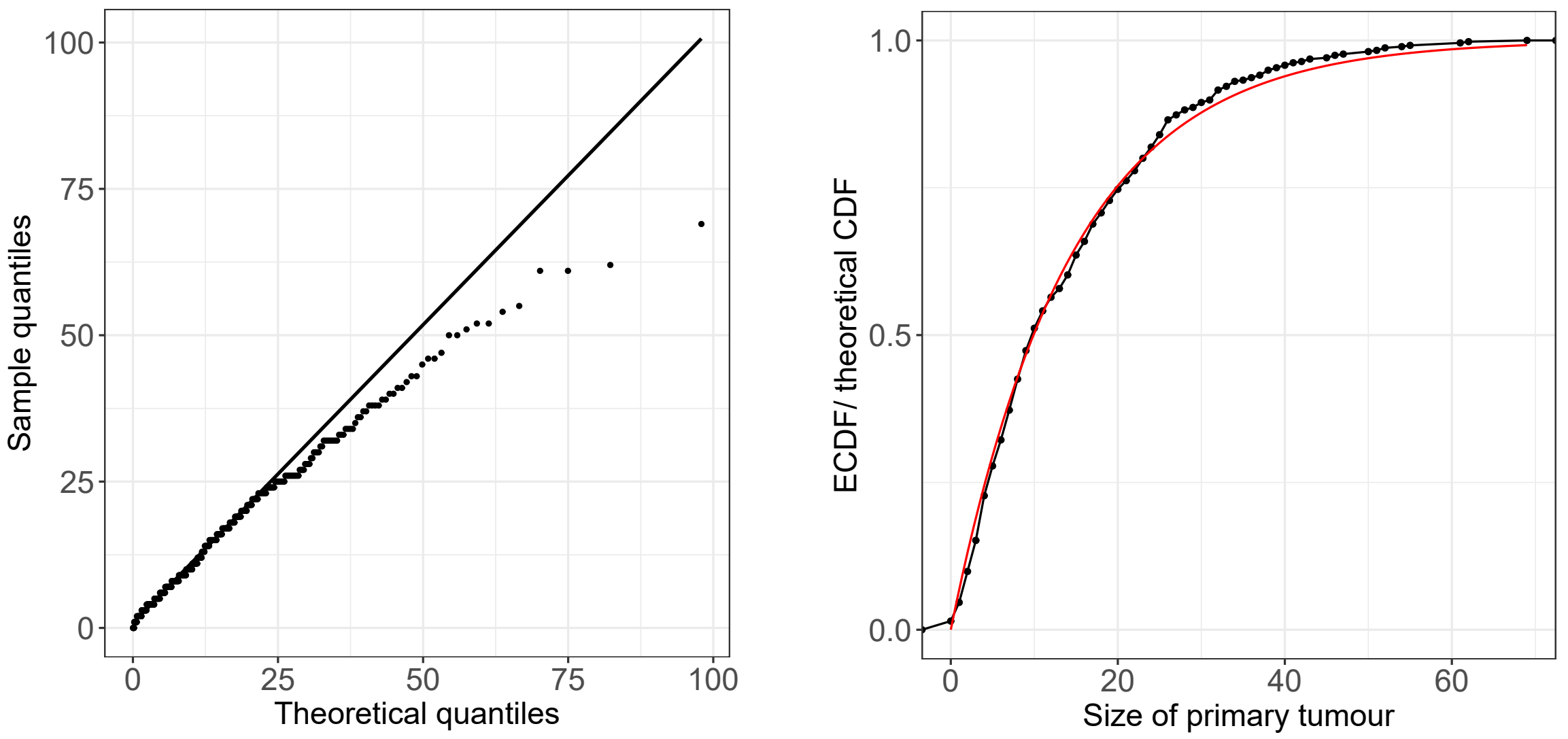

Fig. B.2e Distribution of size of primary tumour compared to the truncated exponential distribution with $\lambda = 0.07$.

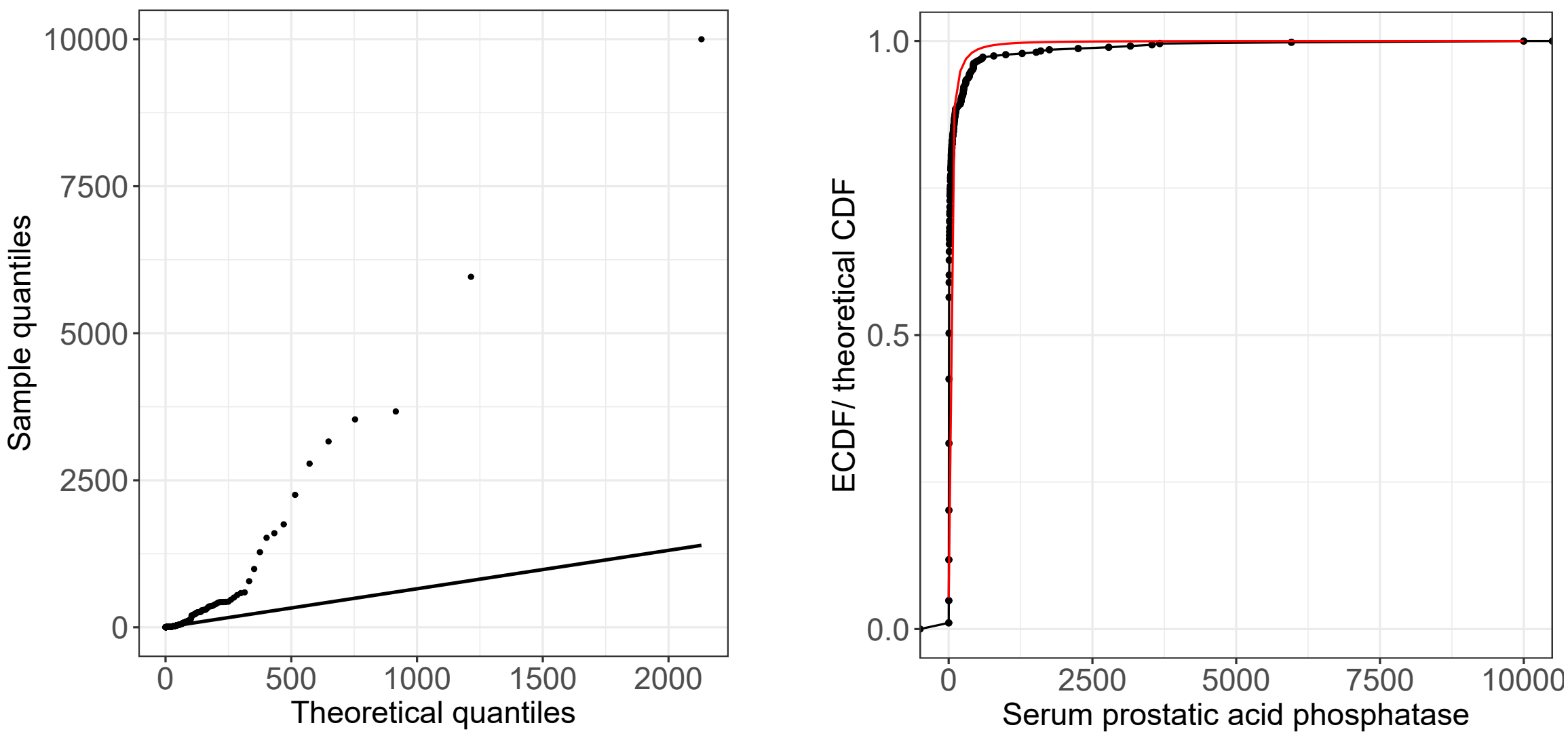

Fig. B.2f Distribution of serum (prostatic) acid phosphatase compared to the truncated log-normal distribution with $\log(\mu) = 2.64$, and $\log(\sigma) = 1.63$.

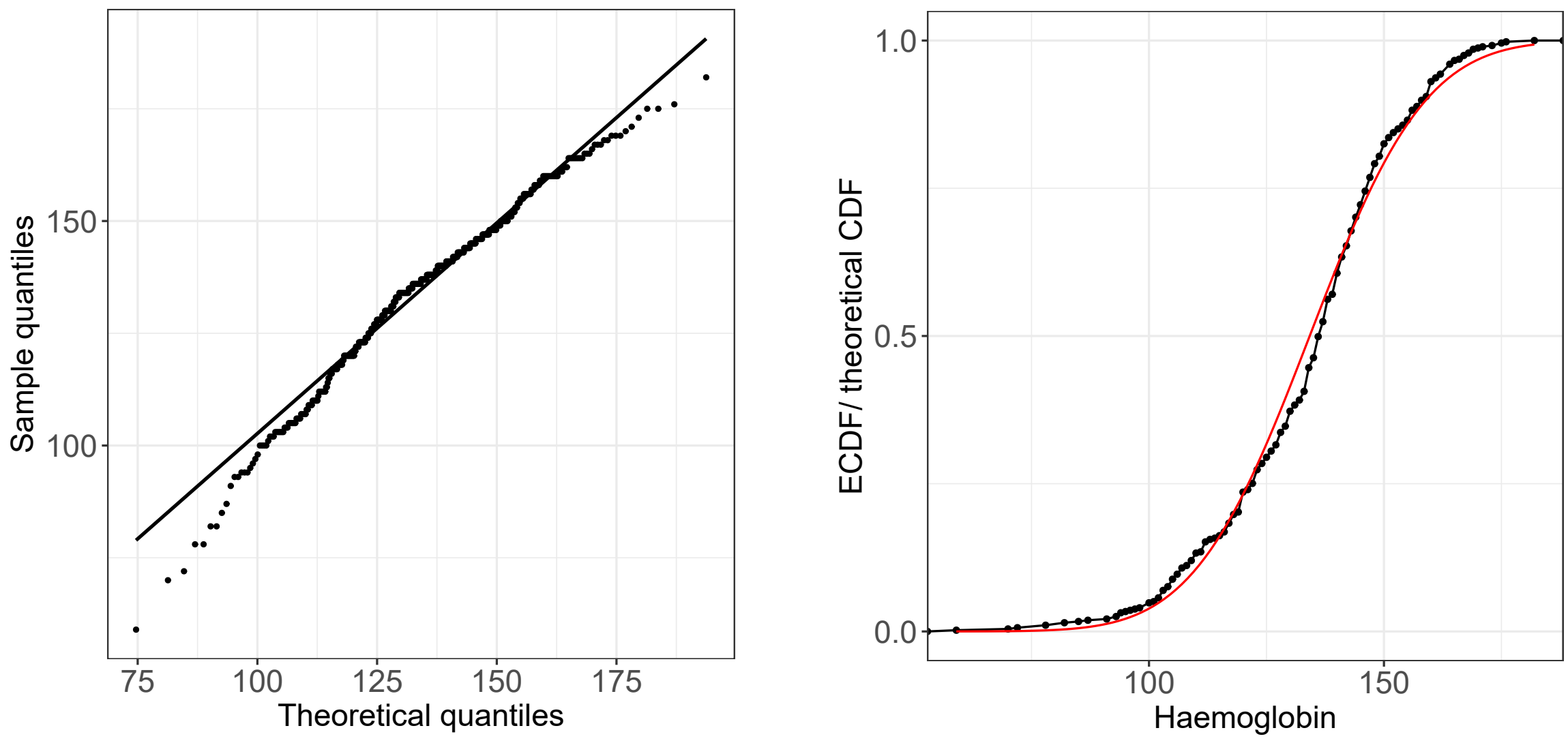

Fig. B.2g Distribution of haemoglobin compared to the truncated normal distribution with $\mu = 134.2$, and $\sigma = 19.36$.

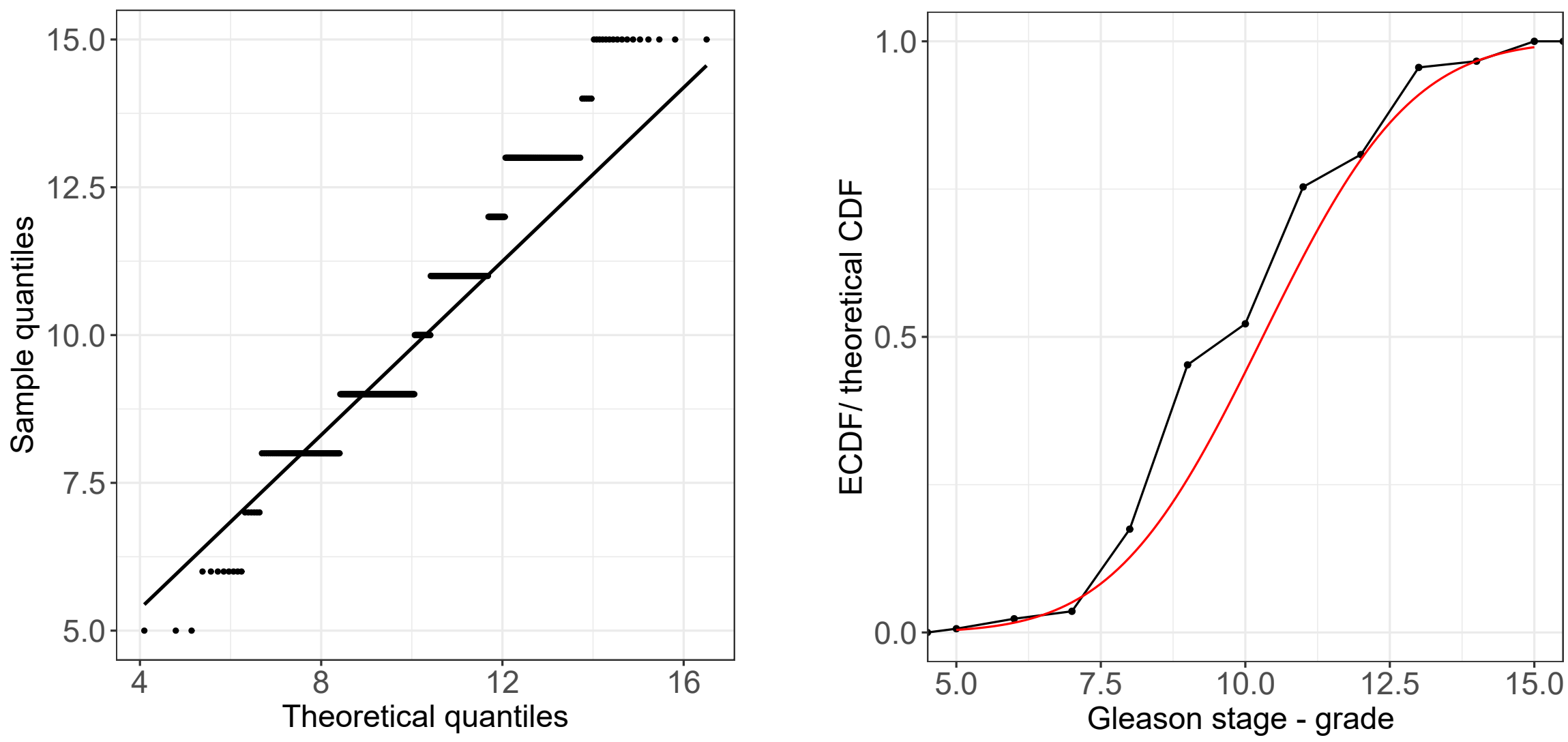

Fig. B.2h Distribution of Gleason stage-grade category compared to the truncated normal distribution with $\mu = 10.3$, and $\sigma = 2.01$.

B.2: **Comparison of the true covariate distribution with the distribution used in the copula model (prostate cancer dataset).** QQ-plot (left) and plot comparing the theoretical and empirical cumulative distribution functions (right) based on maximum likelihood estimates of the respective distributional parameters for each continuous variable in the prostate cancer dataset. Truncated distributions were fitted with lower and upper limits corresponding to the minimum and maximum value of the variable.

C: Simulation study results

In this section, full simulation study results of the C index (B.1), Integrated Brier score (B.2), and calibration curves (B.3) are shown, i.e. boxplots of the estimates' distribution and tables of means and standard errors for the 500 simulated datasets per scenario.

C.1: C index

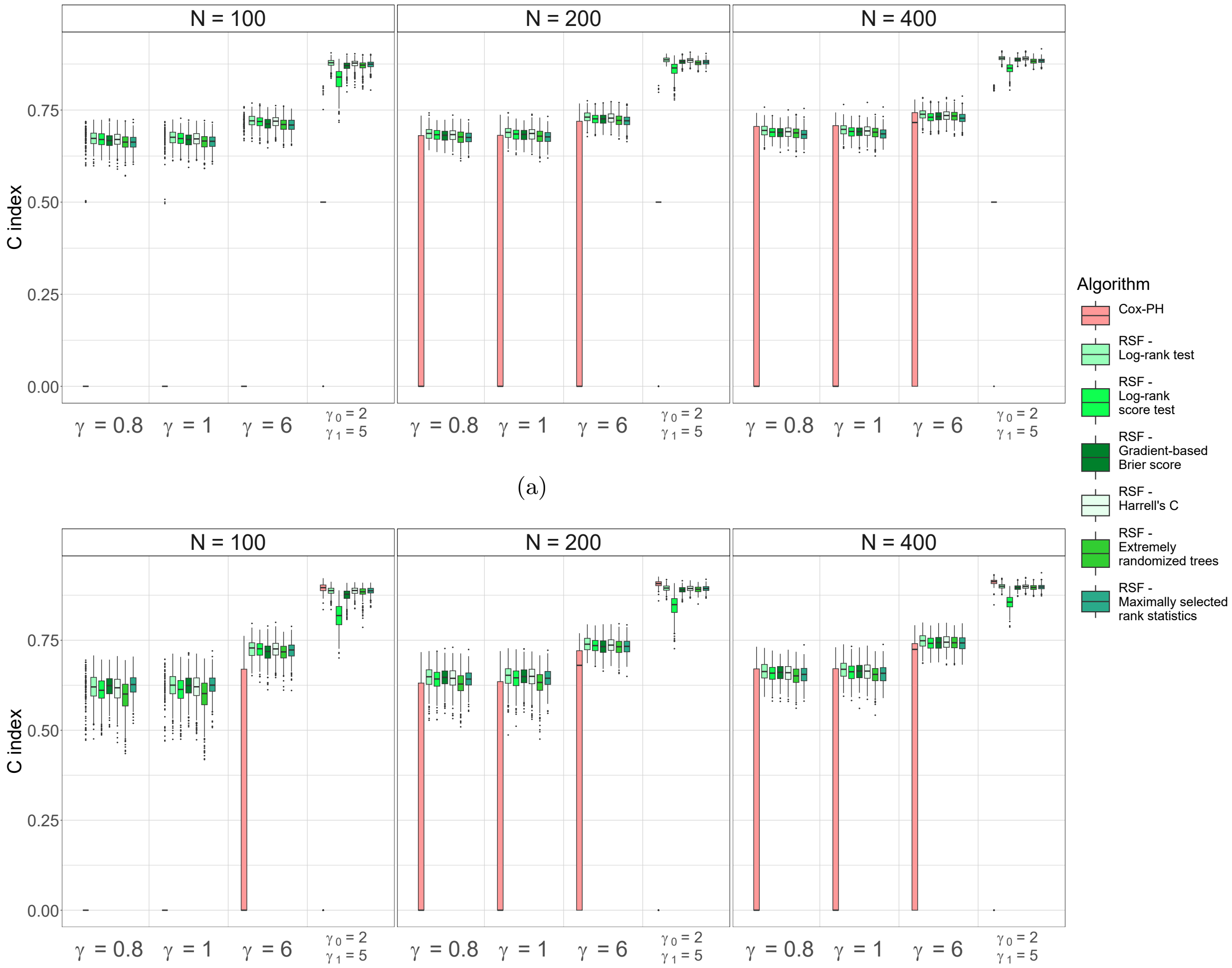

(a)

(b)

Fig. C.1a: Distribution of C index estimates for the Cox and RSF model when applied to the simulated data (primary biliary cirrhosis dataset, $\beta_{\text{treatment}} = 0$). Boxplots showing the distribution of the C index estimates obtained from 500 simulated datasets based on data without treatment-covariate interactions (primary biliary cirrhosis dataset) for the regression coefficient of the treatment effect $\beta_{\text{treatment}} = 0$. The scale parameter of the Weibull distributed survival times ($\lambda = 2241.74$) is chosen to be constant, shape parameters (γ) vary ($\gamma = 0.8$: decreasing hazard, $\gamma = 1$: constant hazard, $\gamma = 6$: increasing hazard, $\{\gamma_0 = 2, \gamma_1 = 5\}$: nonproportional hazards). Results are shown for different total sample sizes N, and censoring rates (a: 30%, b: 60%).

Abbreviations: Cox-PH - Cox proportional hazards model, RSF - Random survival forest.

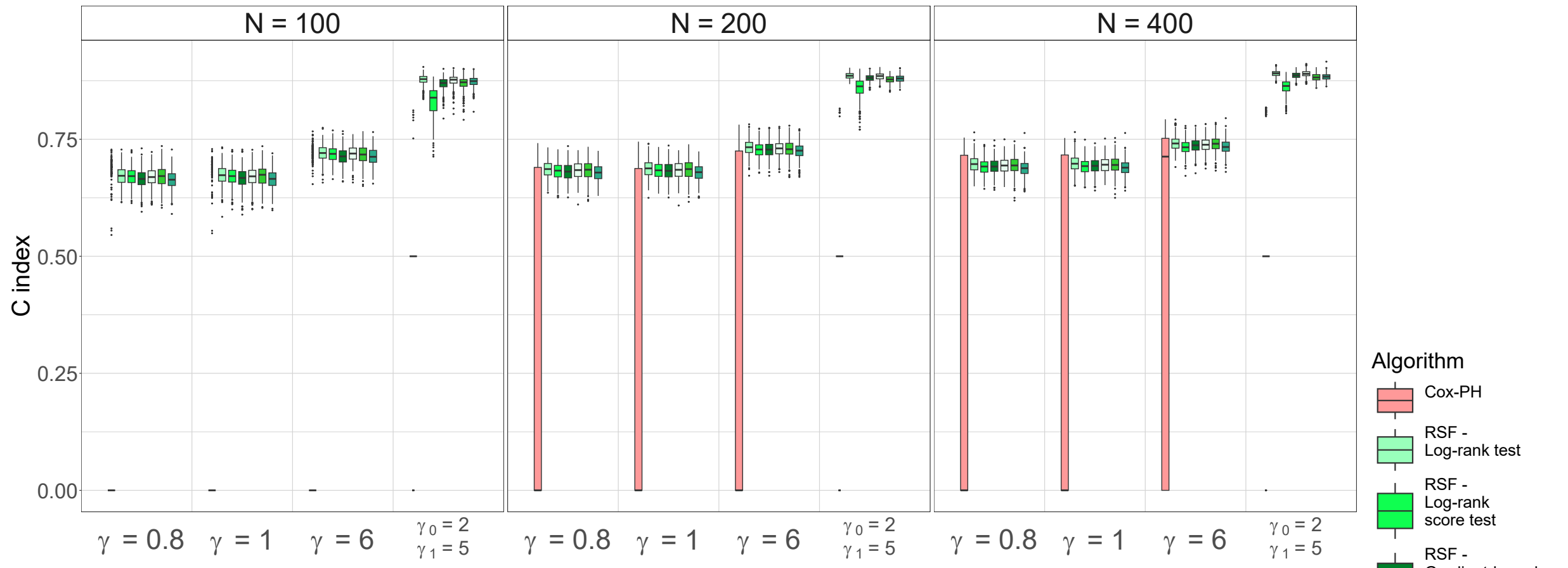

(a)

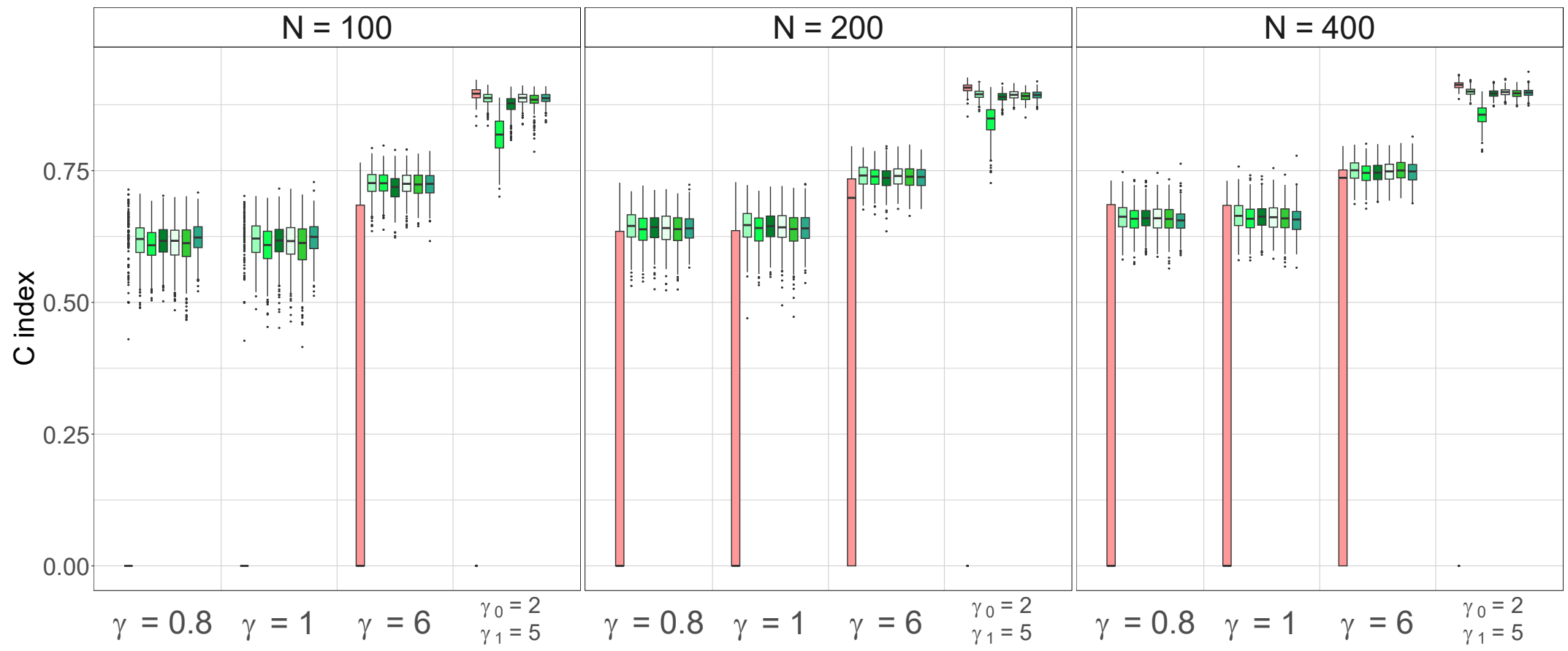

(b)

Fig. C.1b: Distribution of C index estimates for the Cox and RSF model when applied to the simulated data (primary biliary cirrhosis dataset, $\beta_{\text{treatment}} = 0.8$). Boxplots showing the distribution of the C index estimates obtained from 500 simulated datasets based on data without treatment-covariate interactions (primary biliary cirrhosis dataset) for the regression coefficient of the treatment effect $\beta_{\text{treatment}} = 0.8$. The scale parameter of the Weibull distributed survival times ($\lambda = 2241.74$) is chosen to be constant, shape parameters (γ) vary ($\gamma = 0.8$: decreasing hazard, $\gamma = 1$: constant hazard, $\gamma = 6$: increasing hazard, $\{\gamma_0 = 2, \gamma_1 = 5\}$: nonproportional hazards). Results are shown for different total sample sizes N, and censoring rates (a: 30%, b: 60%).

Abbreviations: Cox-PH - Cox proportional hazards model, RSF - Random survival forest.

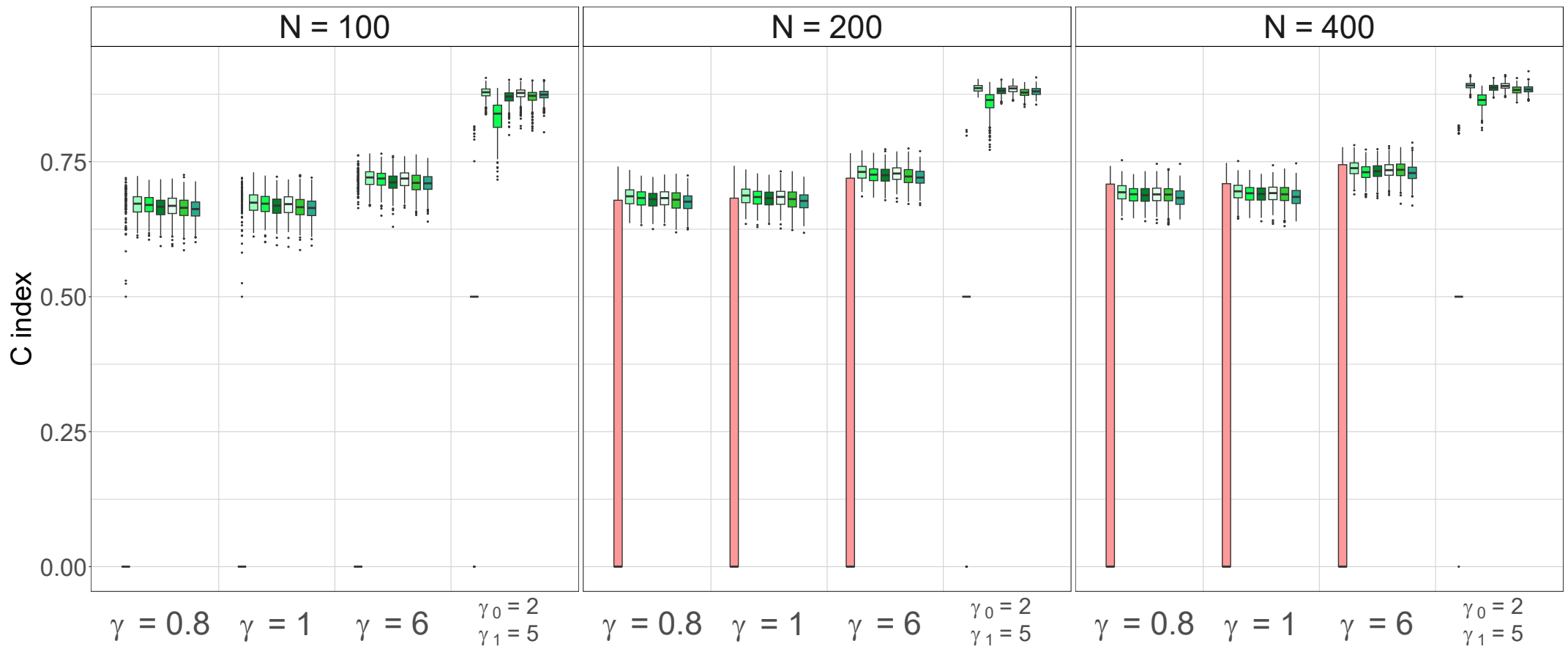

(a)

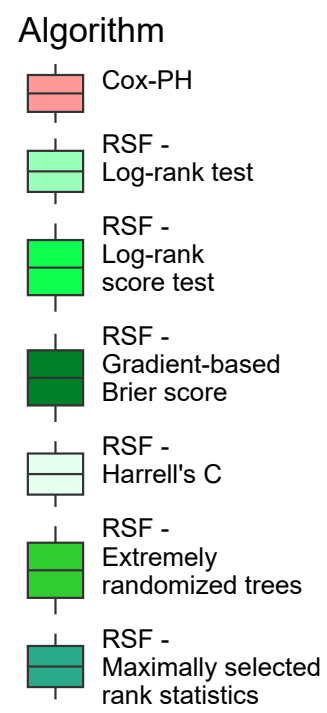

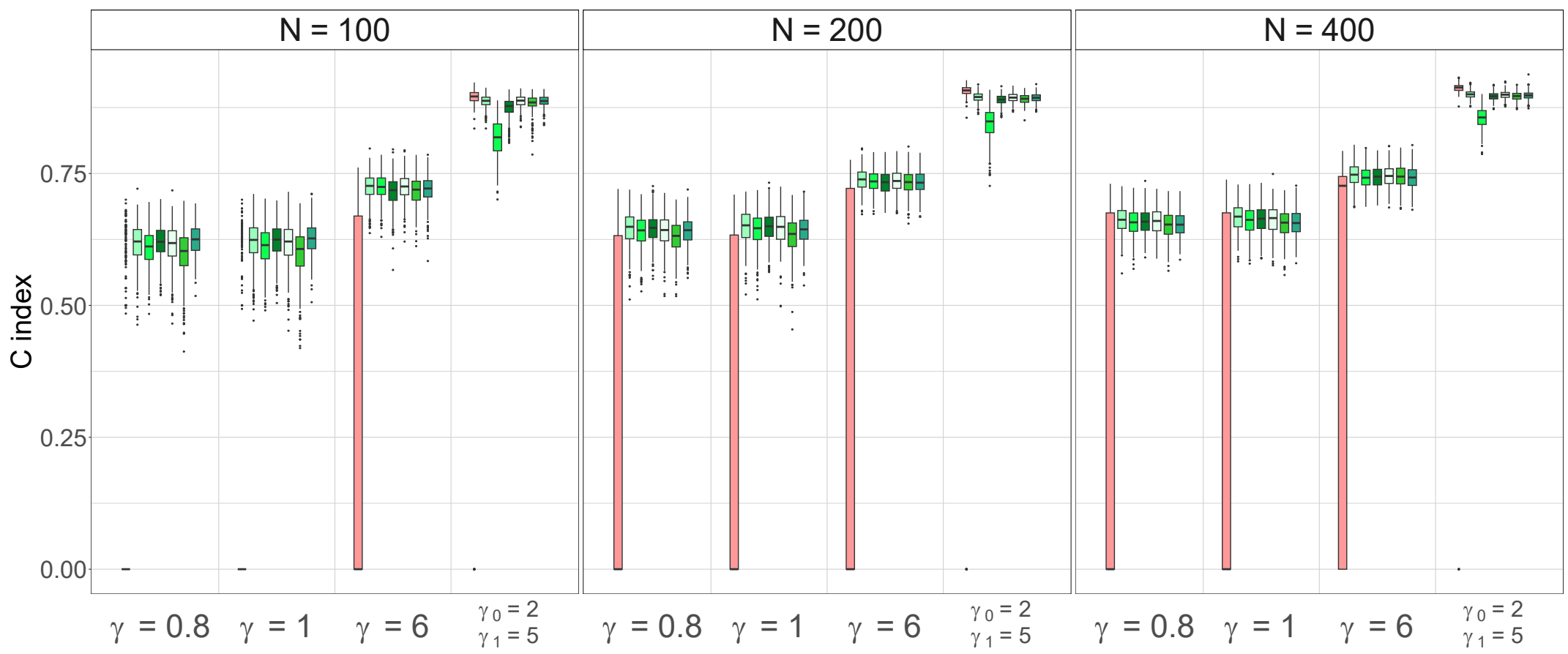

(b)

Fig. C.1c: Distribution of C index estimates for the Cox and RSF model when applied to the simulated data (primary biliary cirrhosis dataset, $\beta_{\text{treatment}} = -0.4$). Boxplots showing the distribution of the C index estimates obtained from 500 simulated datasets based on data without treatment-covariate interactions (primary biliary cirrhosis dataset) for the regression coefficient of the treatment effect $\beta_{\text{treatment}} = -0.4$. The scale parameter of the Weibull distributed survival times ($\lambda = 2241.74$) is chosen to be constant, shape parameters (γ) vary ($\gamma = 0.8$: decreasing hazard, $\gamma = 1$: constant hazard, $\gamma = 6$: increasing hazard, $\{\gamma_0 = 2, \gamma_1 = 5\}$: nonproportional hazards). Results are shown for different total sample sizes N, and censoring rates (a: 30%, b: 60%).

Abbreviations: Cox-PH - Cox proportional hazards model, RSF - Random survival forest.

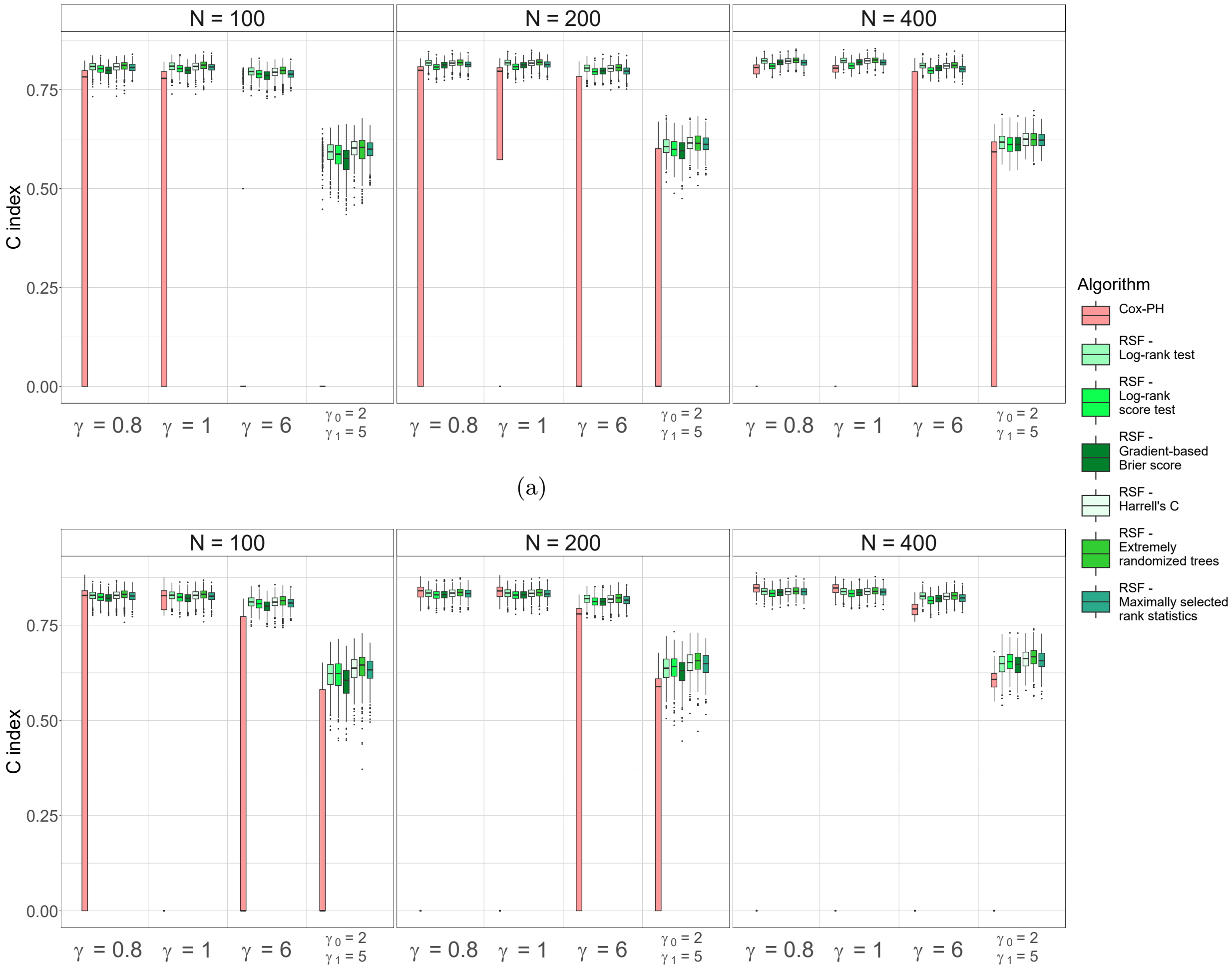

(a)

(b)

Fig. C.2a: **Distribution of** C **index estimates for the Cox and RSF model when applied to the simulated data (prostate cancer dataset,** $\beta_{\text{treatment}} = 0$**).** Boxplots showing the distribution of the C index estimates obtained from 500 simulated datasets based on data with three treatment-covariate interactions (prostate cancer dataset) for the regression coefficient of the treatment effect $\beta_{\text{treatment}} = 0$. The scale parameter of the Weibull distributed survival times ($\lambda = 39.2$) is chosen to be constant, shape parameters (γ) vary ($\gamma = 0.8$: decreasing hazard, $\gamma = 1$: constant hazard, $\gamma = 6$: increasing hazard, $\{\gamma_0 = 2, \gamma_1 = 5\}$: nonproportional hazards). Results are shown for different total sample sizes N, and censoring rates (a: 30%, b: 60%).

Abbreviations: Cox-PH - Cox proportional hazards model, RSF - Random survival forest.

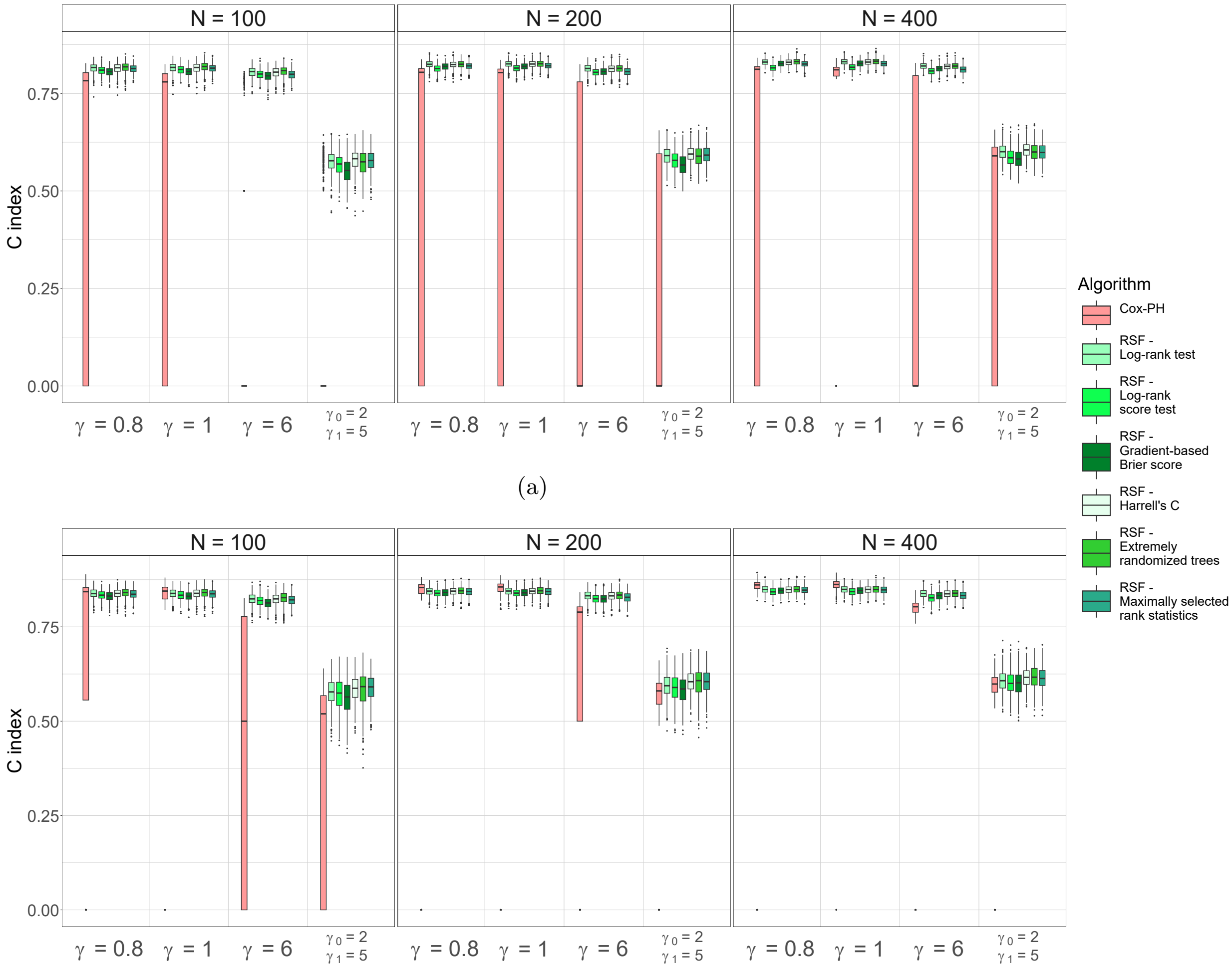

(a)

(b)

Fig. C.2b: Distribution of C index estimates for the Cox and RSF model when applied to the simulated data (prostate cancer dataset, $\beta_{\text{treatment}} = 0.8$). Boxplots showing the distribution of the C index estimates obtained from 500 simulated datasets based on data with three treatment-covariate interactions (prostate cancer dataset) for the regression coefficient of the treatment effect $\beta_{\text{treatment}} = 0.8$. The scale parameter of the Weibull distributed survival times ($\lambda = 39.2$) is chosen to be constant, shape parameters (γ) vary ($\gamma = 0.8$: decreasing hazard, $\gamma = 1$: constant hazard, $\gamma = 6$: increasing hazard, $\{\gamma_0 = 2, \gamma_1 = 5\}$: nonproportional hazards). Results are shown for different total sample sizes N, and censoring rates (a: 30%, b: 60%).

Abbreviations: Cox-PH - Cox proportional hazards model, RSF - Random survival forest.

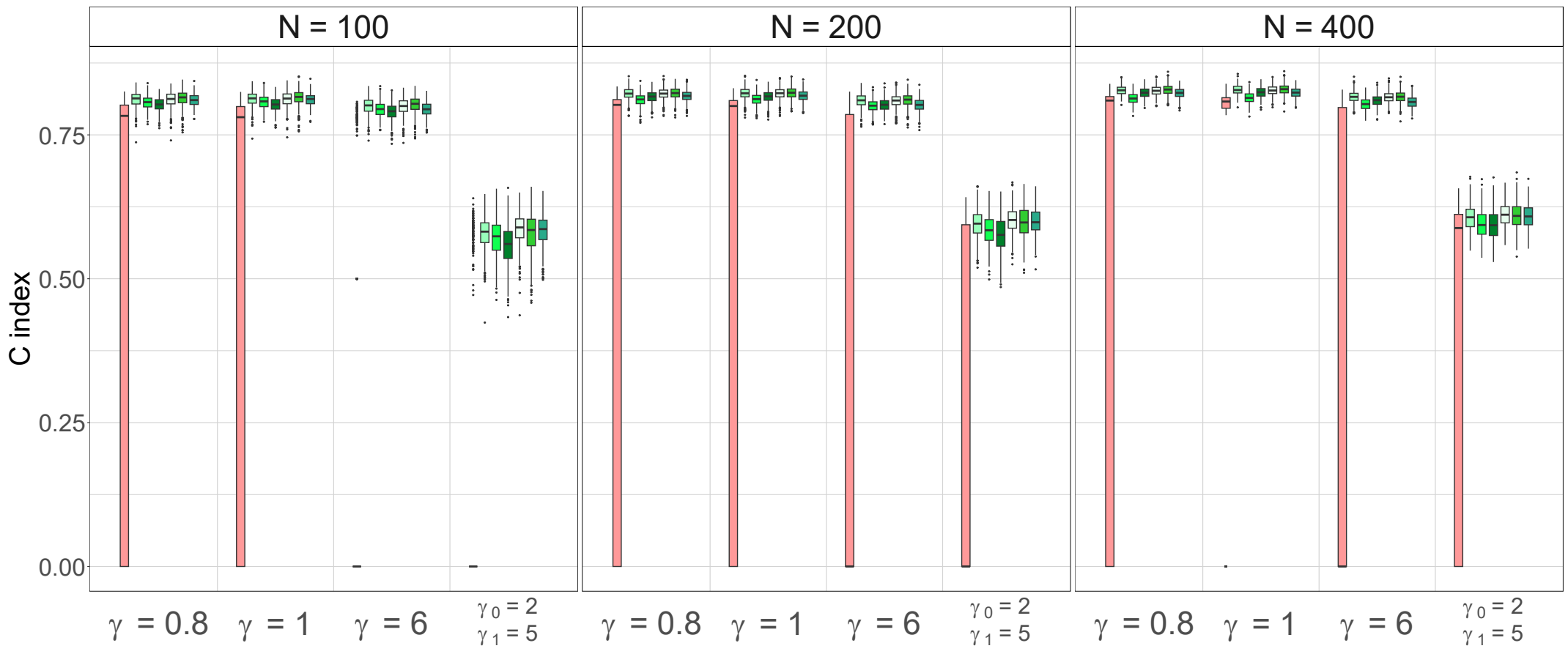

(a)

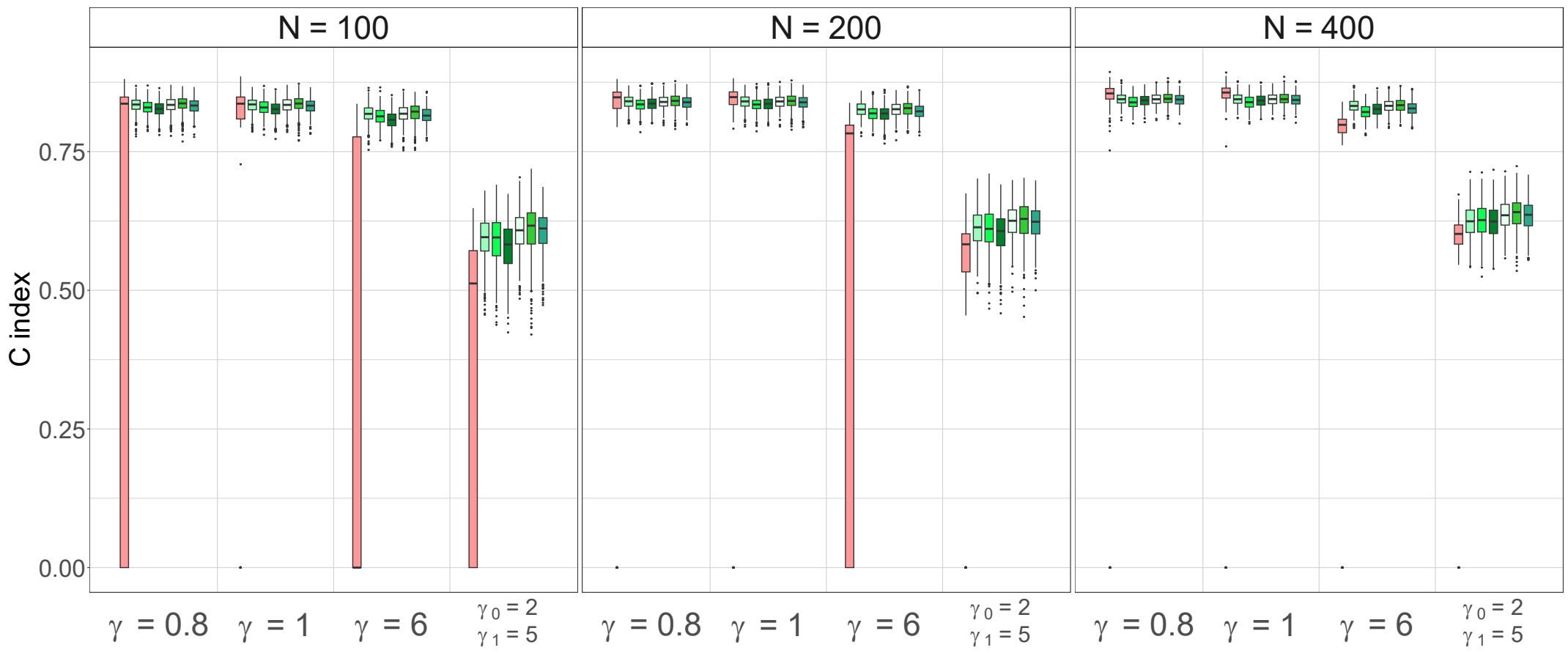

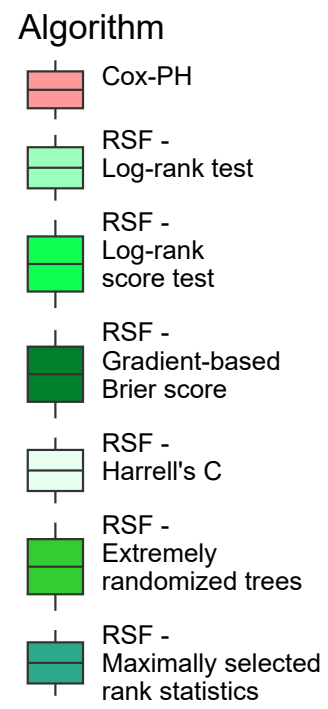

(b)

Fig. C.2c: **Distribution of C index estimates for the Cox and RSF model when applied to the simulated data (prostate cancer dataset, $\beta_{\text{treatment}} = -0.4$).** Boxplots showing the distribution of the C index estimates obtained from 500 simulated datasets based on data with three treatment-covariate interactions (prostate cancer dataset) for the regression coefficient of the treatment effect $\beta_{\text{treatment}} = -0.4$. The scale parameter of the Weibull distributed survival times ($\lambda = 39.2$) is chosen to be constant, shape parameters (γ) vary. Results are shown for different total sample sizes N, and censoring rates (a: 30%, b: 60%).

Abbreviations: Cox-PH - Cox proportional hazards model, RSF - Random survival forest.

C.2: Integrated Brier score (IBS)

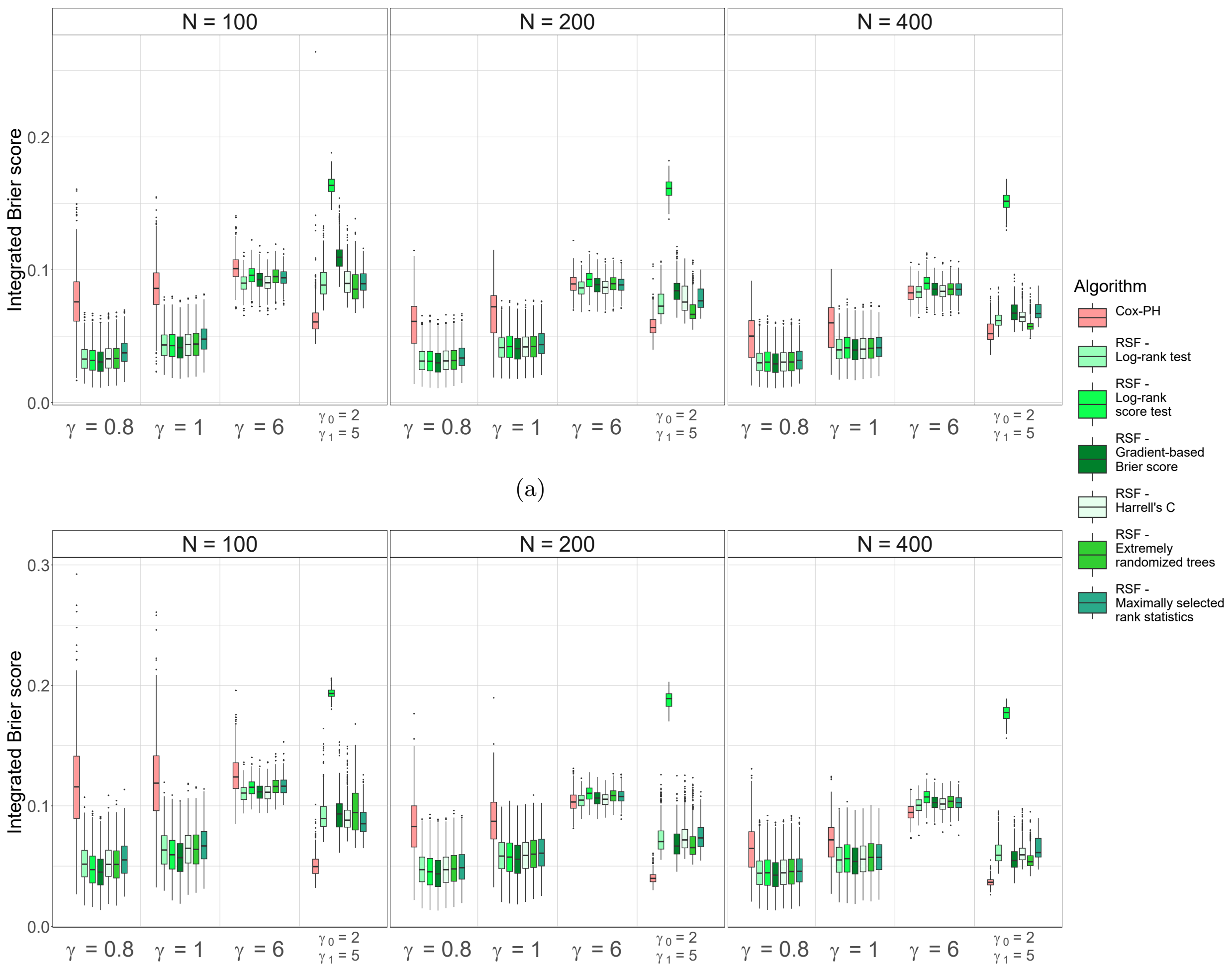

Fig. C.3a: **Distribution of Integrated Brier score (IBS) estimates for the Cox and RSF model when applied to the simulated data (primary biliary cirrhosis dataset, $\beta_{\text{treatment}} = 0$).** Boxplots showing the distribution of the Integrated Brier score (IBS) estimates obtained from 500 simulated datasets based on data without treatment-covariate interactions (primary biliary cirrhosis dataset) for the regression coefficient of the treatment effect $\beta_{\text{treatment}} = 0$. The scale parameter of the Weibull distributed survival times ($\lambda = 2241.74$) is chosen to be constant, shape parameters (γ) vary ($\gamma = 0.8$: decreasing hazard, $\gamma = 1$: constant hazard, $\gamma = 6$: increasing hazard, $\{\gamma_0 = 2, \gamma_1 = 5\}$: nonproportional hazards). Results are shown for different total sample sizes N, and censoring rates (a: 30%, b: 60%).

Abbreviations: Cox-PH - Cox proportional hazards model, RSF - Random survival forest.

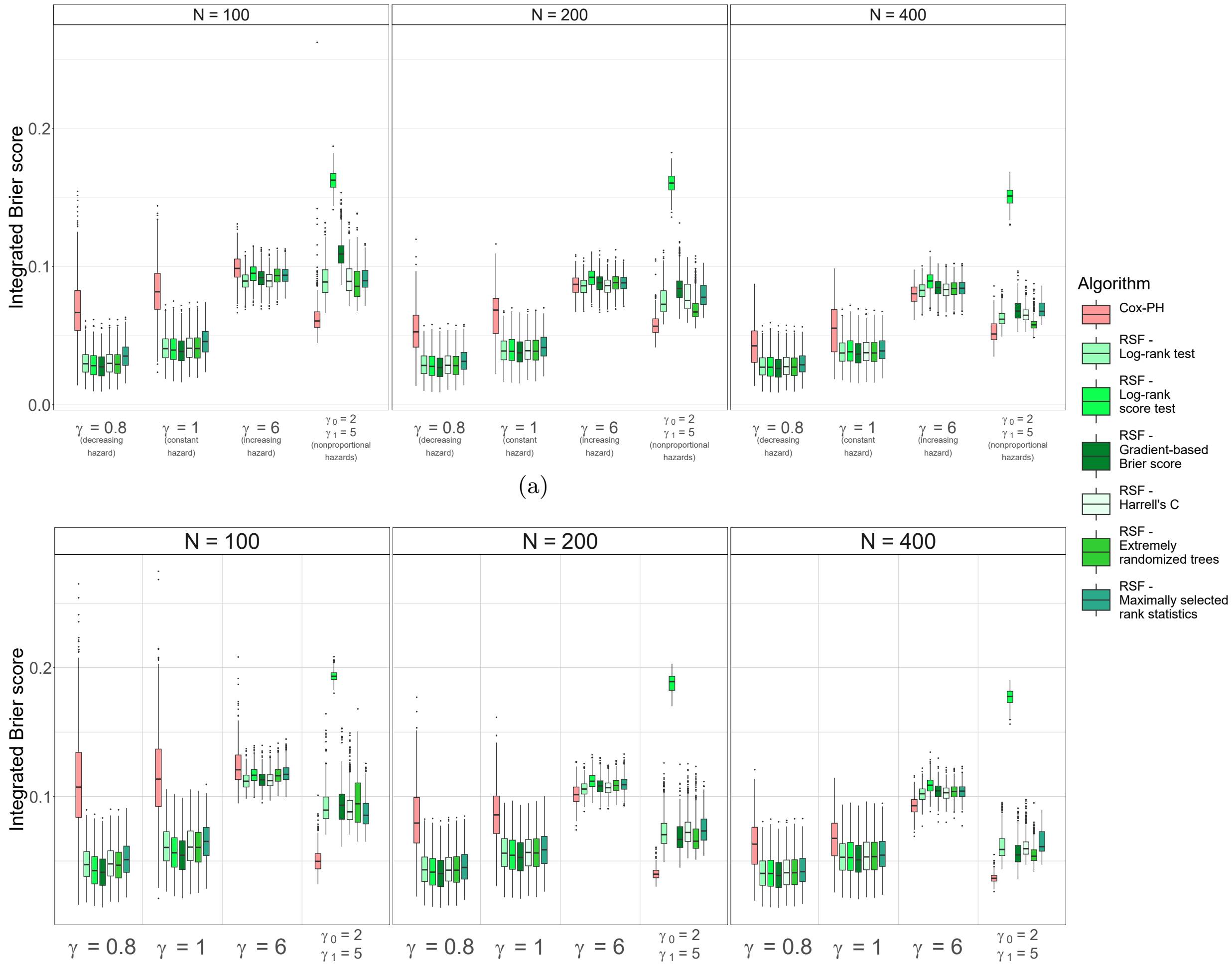

(a)

(b)

Fig. C.3b: Distribution of Integrated Brier score (IBS) estimates for the Cox and RSF model when applied to the simulated data (primary biliary cirrhosis dataset, $\beta_{\text{treatment}} = 0.8$). Boxplots showing the distribution of the Integrated Brier score (IBS) estimates obtained from 500 simulated datasets based on data without treatment-covariate interactions (primary biliary cirrhosis dataset) for the regression coefficient of the treatment effect $\beta_{\text{treatment}} = 0.8$. The scale parameter of the Weibull distributed survival times ($\lambda = 2241.74$) is chosen to be constant, shape parameters (γ) vary ($\gamma = 0.8$: decreasing hazard, $\gamma = 1$: constant hazard, $\gamma = 6$: increasing hazard, $\{\gamma_0 = 2, \gamma_1 = 5\}$: nonproportional hazards). Results are shown for different total sample sizes N, and censoring rates (a: 30%, b: 60%).

Abbreviations: Cox-PH - Cox proportional hazards model, RSF - Random survival forest.

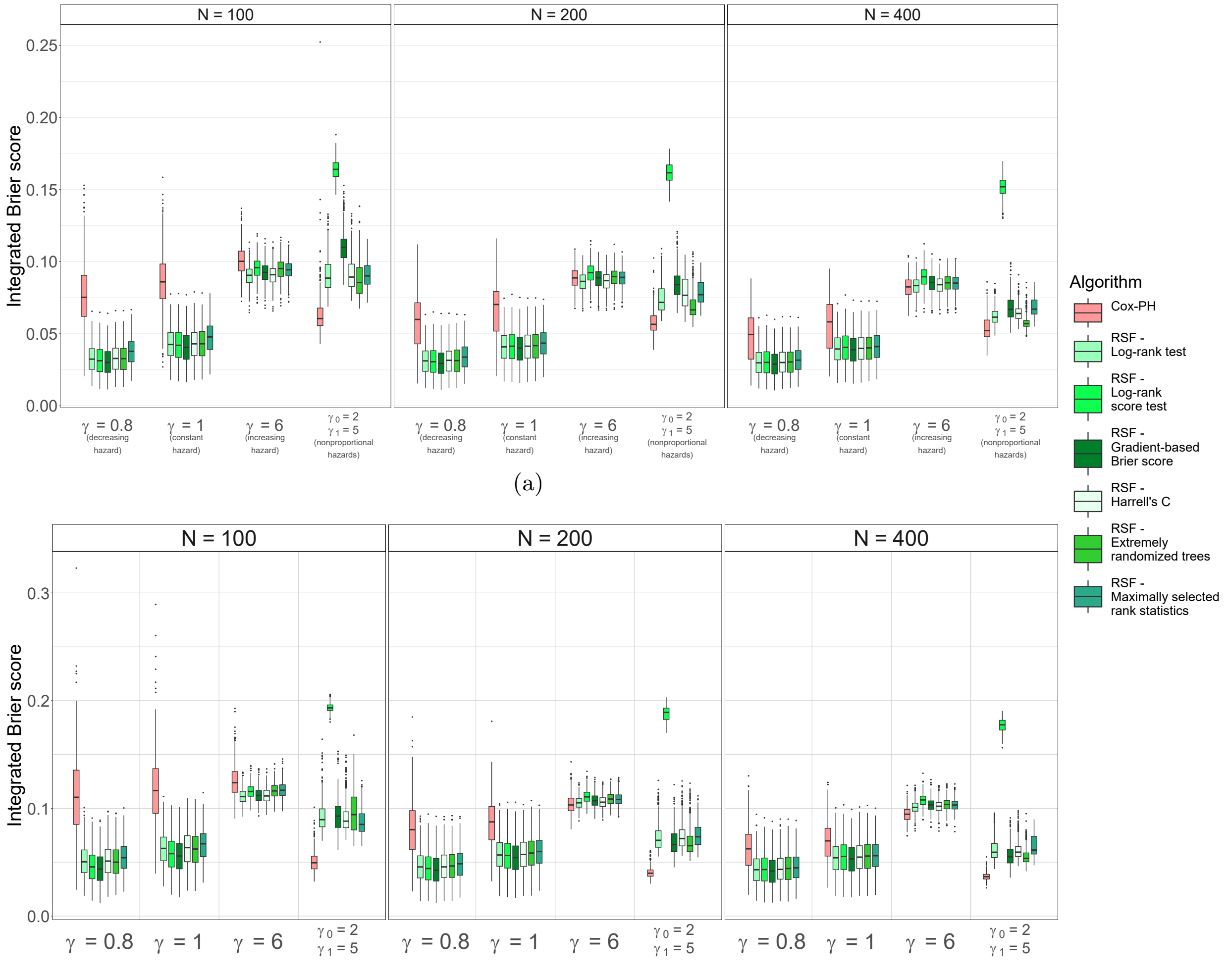

(a) (b)

Fig. C.3c: Distribution of Integrated Brier score (IBS) estimates for the Cox and RSF model when applied to the simulated data (primary biliary cirrhosis dataset, $\beta_{\text{treatment}} = -0.4$). Boxplots showing the distribution of the Integrated Brier score (IBS) estimates obtained from 500 simulated datasets based on data without treatment-covariate interactions (primary biliary cirrhosis dataset) for the regression coefficient of the treatment effect $\beta_{\text{treatment}} = -0.4$. The scale parameter of the Weibull distributed survival times ($\lambda = 2241.74$) is chosen to be constant, shape parameters (γ) vary ($\gamma = 0.8$: decreasing hazard, $\gamma = 1$: constant hazard, $\gamma = 6$: increasing hazard, $\{\gamma_0 = 2, \gamma_1 = 5\}$: nonproportional hazards). Results are shown for different total sample sizes N, and censoring rates (a: 30%, b: 60%).

Abbreviations: Cox-PH - Cox proportional hazards model, RSF - Random survival forest.

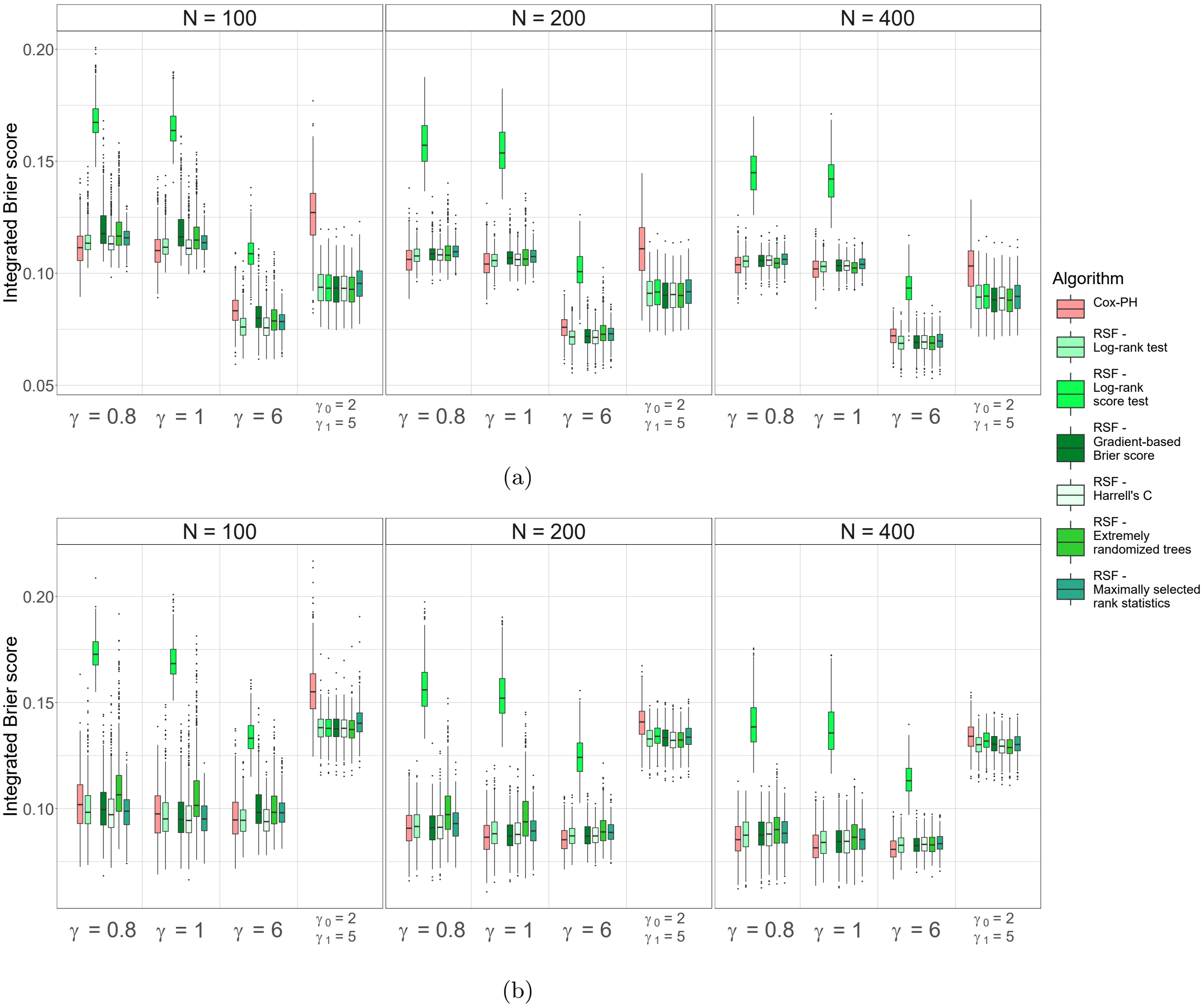

Fig. C.4a: Distribution of Integrated Brier score (IBS) estimates for the Cox and RSF model when applied to the simulated data (prostate cancer dataset, $\beta_{\text{treatment}} = 0$). Boxplots showing the distribution of the Integrated Brier score (IBS) estimates obtained from 500 simulated datasets based on data with three treatment-covariate interactions (prostate cancer dataset) for the regression coefficient of the treatment effect $\beta_{\text{treatment}} = 0$. The scale parameter of the Weibull distributed survival times ($\lambda = 39.2$) is chosen to be constant, shape parameters (γ) vary ($\gamma = 0.8$: decreasing hazard, $\gamma = 1$: constant hazard, $\gamma = 6$: increasing hazard, $\{\gamma_0 = 2, \gamma_1 = 5\}$: nonproportional hazards). Results are shown for different total sample sizes N, and censoring rates (a: 30%, b: 60%).

Abbreviations: Cox-PH - Cox proportional hazards model, RSF - Random survival forest.

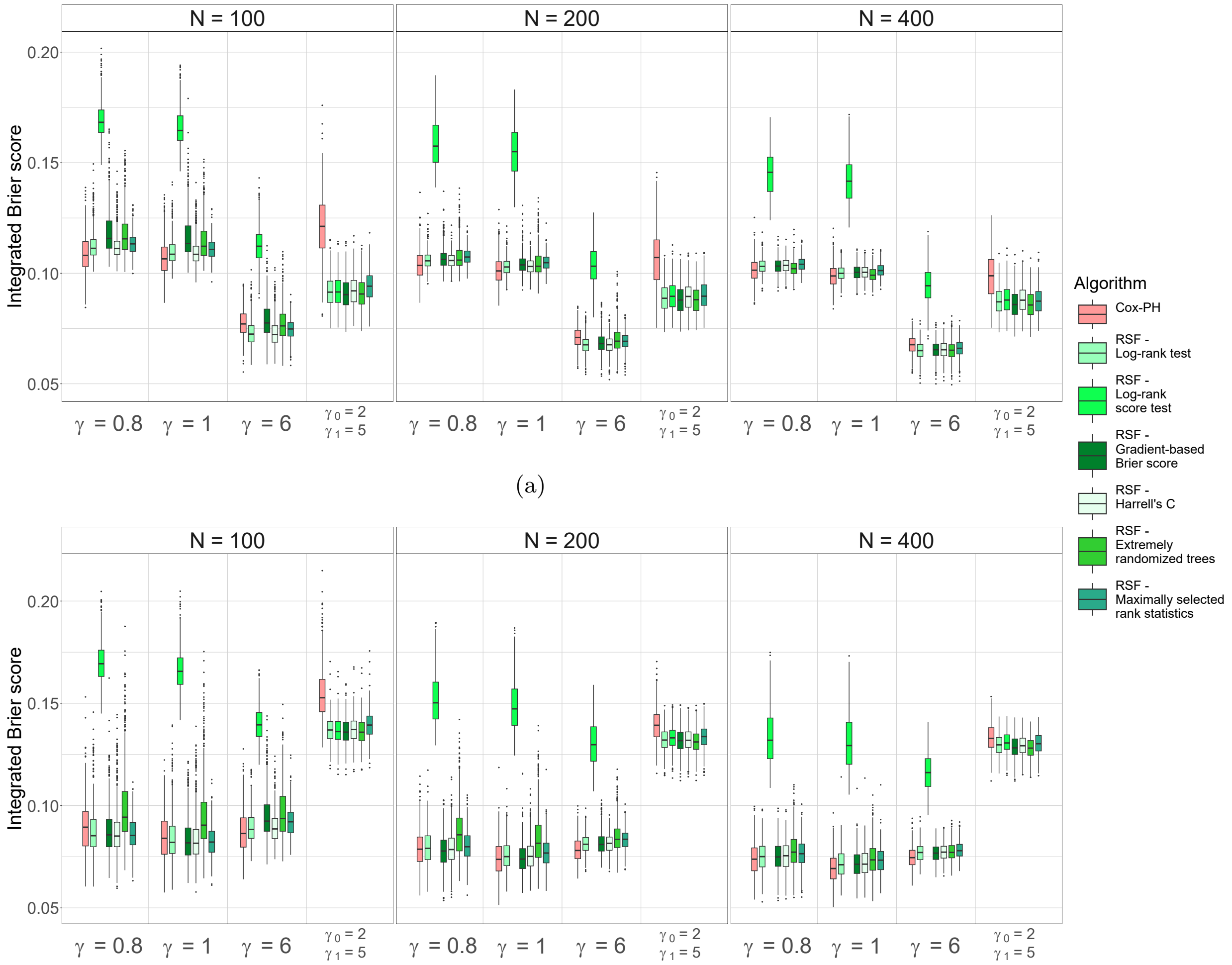

(a)

(b)

Fig. C.4b: Distribution of Integrated Brier score (IBS) estimates for the Cox and RSF model when applied to the simulated data (prostate cancer dataset, $\beta_{\text{treatment}} = 0.8$). Boxplots showing the distribution of the Integrated Brier score (IBS) estimates obtained from 500 simulated datasets based on data with three treatment-covariate interactions (prostate cancer dataset) for the regression coefficient of the treatment effect $\beta_{\text{treatment}} = 0.8$. The scale parameter of the Weibull distributed survival times ($\lambda = 39.2$) is chosen to be constant, shape parameters (γ) vary ($\gamma = 0.8$: decreasing hazard, $\gamma = 1$: constant hazard, $\gamma = 6$: increasing hazard, $\{\gamma_0 = 2, \gamma_1 = 5\}$: nonproportional hazards). Results are shown for different total sample sizes N, and censoring rates (a: 30%, b: 60%).

Abbreviations: Cox-PH - Cox proportional hazards model, RSF - Random survival forest.

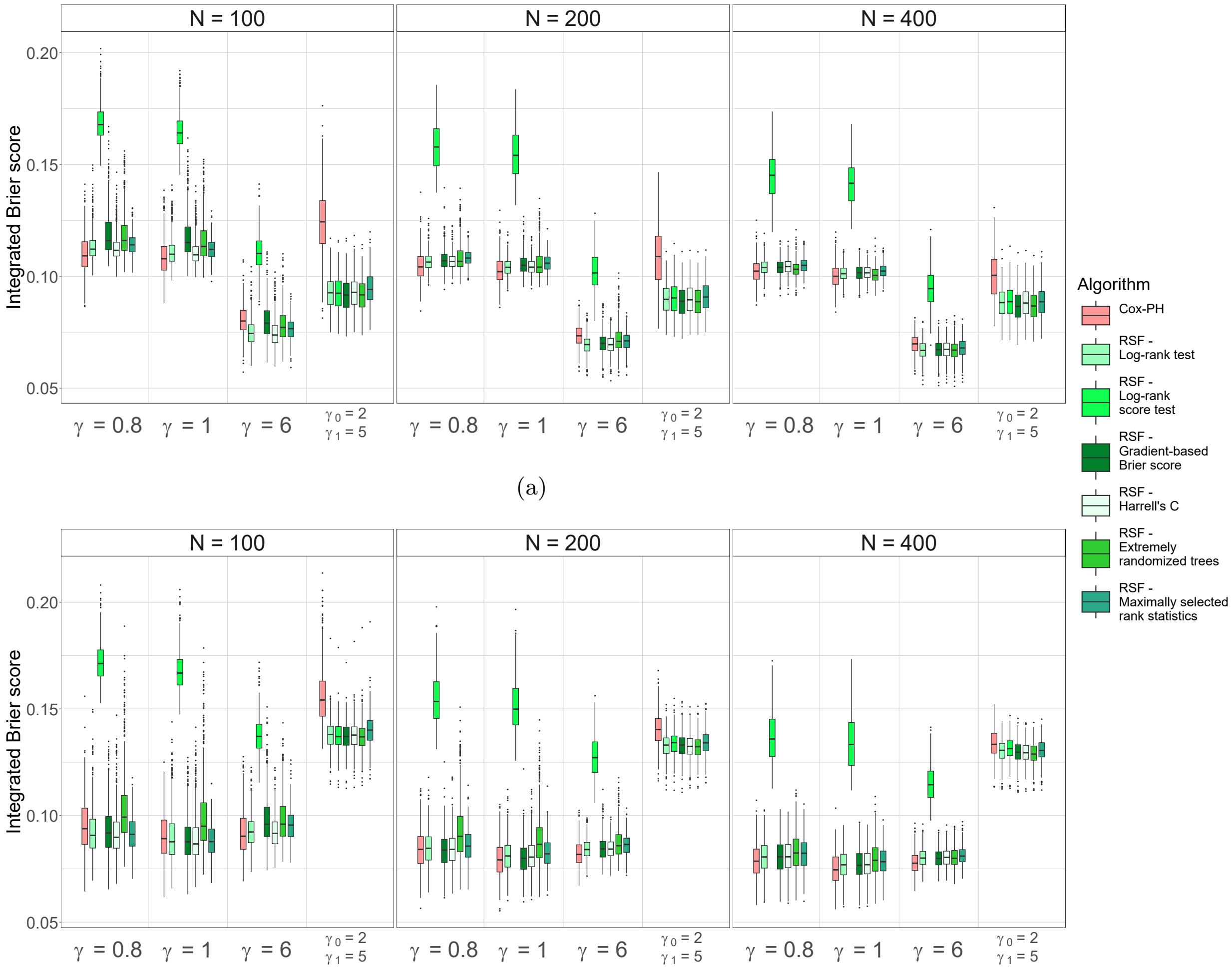

(a)

(b)

Fig. C.4c: Distribution of Integrated Brier score (IBS) estimates for the Cox and RSF model when applied to the simulated data (prostate cancer dataset, $\beta_{\text{treatment}} = -0.4$). Boxplots showing the distribution of the Integrated Brier score (IBS) estimates obtained from 500 simulated datasets based on data with three treatment-covariate interactions (prostate cancer dataset) for the regression coefficient of the treatment effect $\beta_{\text{treatment}} = -0.4$. The scale parameter of the Weibull distributed survival times ($\lambda = 39.2$) is chosen to be constant, shape parameters (γ) vary ($\gamma = 0.8$: decreasing hazard, $\gamma = 1$: constant hazard, $\gamma = 6$: increasing hazard, $\{\gamma_0 = 2, \gamma_1 = 5\}$: nonproportional hazards). Results are shown for different total sample sizes N, and censoring rates (a: 30%, b: 60%).

Abbreviations: Cox-PH - Cox proportional hazards model, RSF - Random survival forest.

C.3: Calibration curves

C.3.1: Calibration curves corresponding to the results shown in the main manuscript

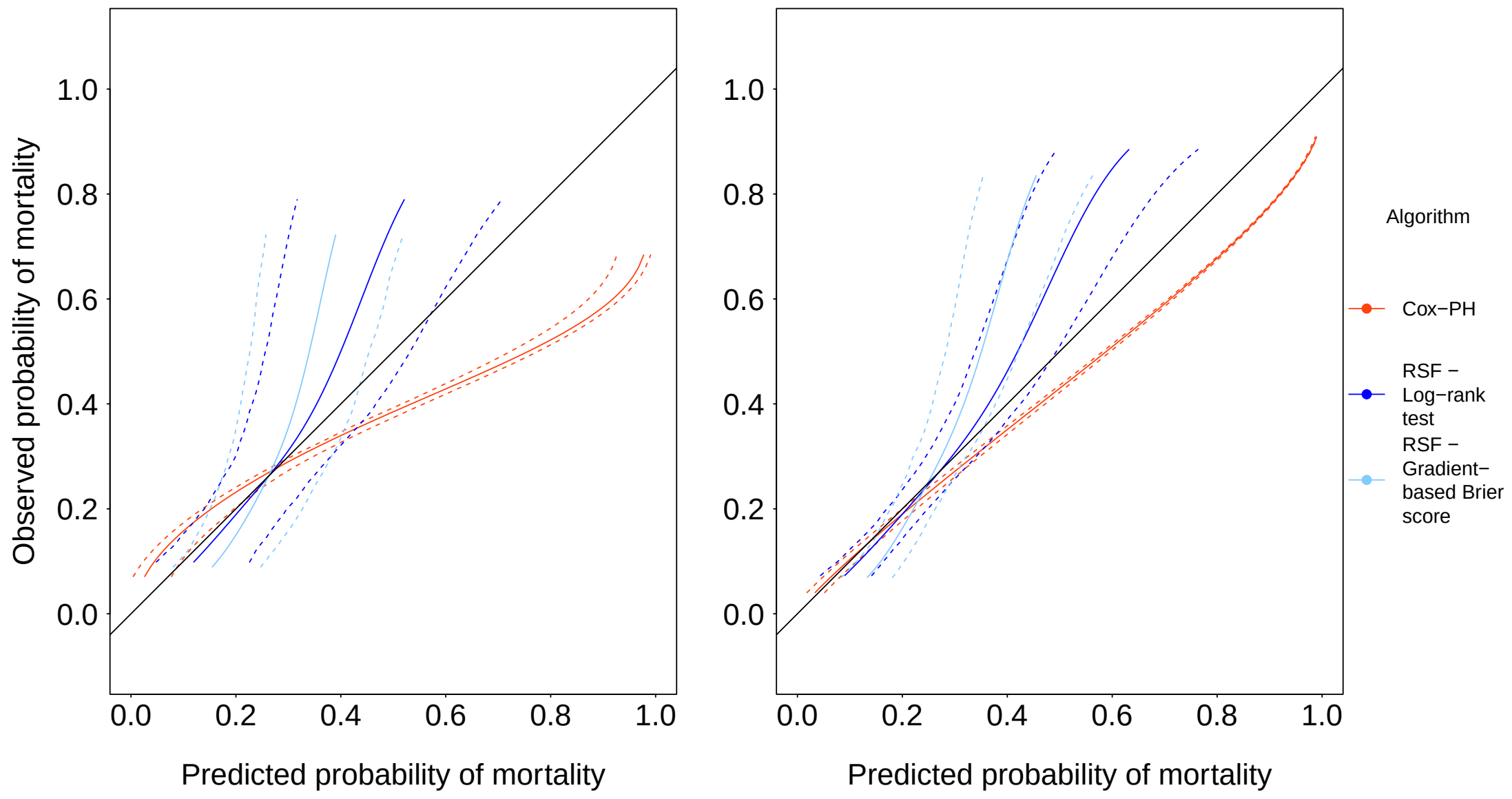

(a) (b)

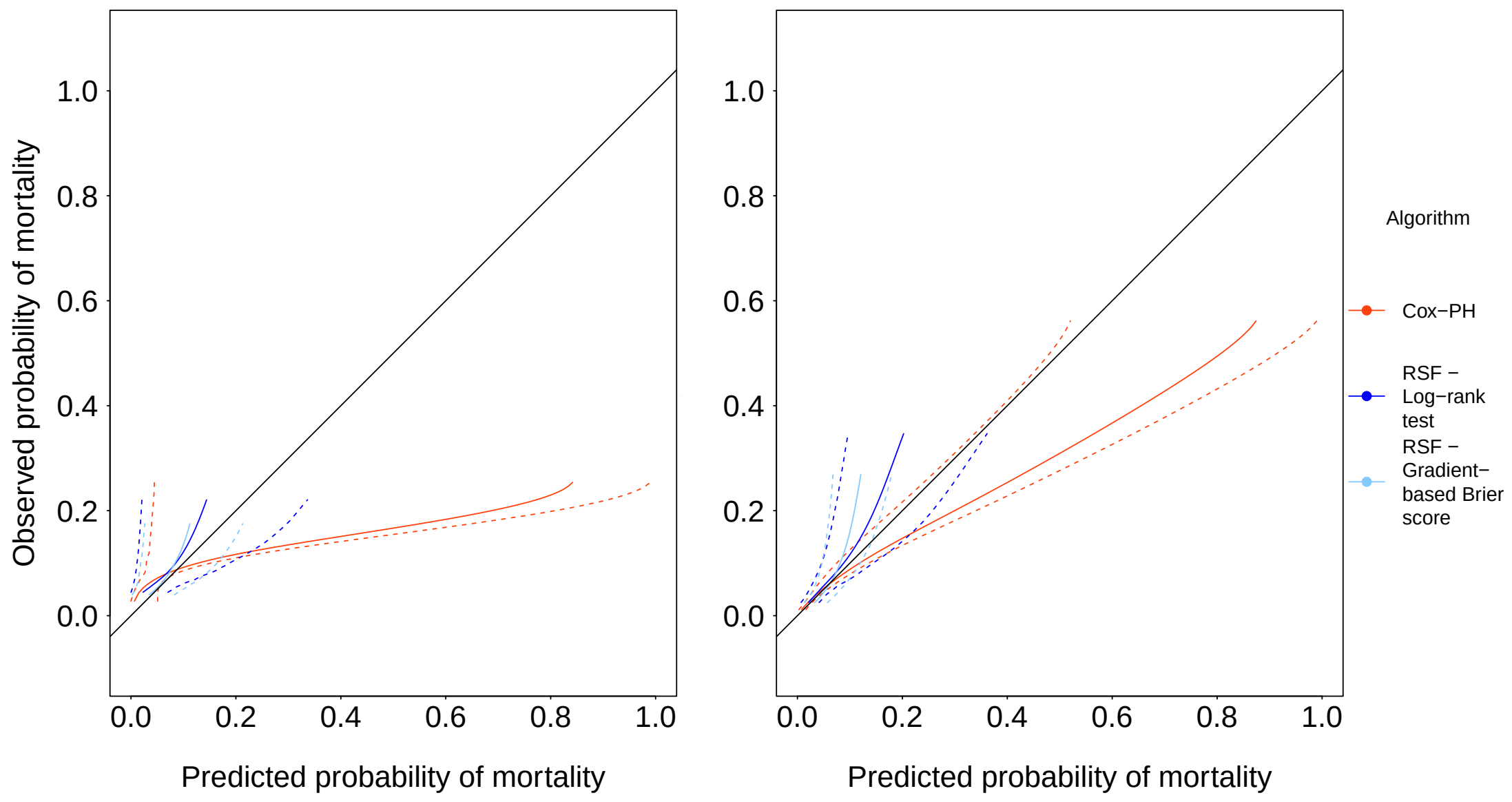

(c) (d)

Fig. C.5a: **Calibration curves for a proportional hazards scenario (primary biliary cirrhosis dataset).** Calibration curves at the median (50% quantile) survival time for a proportional hazards setting (Weibull survival time distribution W(λ = 2241.74, γ = 1)), $\beta_{\text{treatment}}$ = −0.4, and n_{sim} = 500 simulated datasets based on data without treatment-covariate interactions (primary biliary cirrhosis dataset). The solid line represents the mean calibration curve, the outer dotted lines represent the 2.5th and 97.5th percentile of the calibration curve. The black diagonal line corresponds to perfect calibration.
(a) 30% censoring, $N = 100$, (b) 30% censoring, $N = 400$,
(c) 60% censoring, $N = 100$, (d) 60% censoring, $N = 400$.
Abbreviations: Cox-PH - Cox proportional hazards model, RSF - Random survival forest.

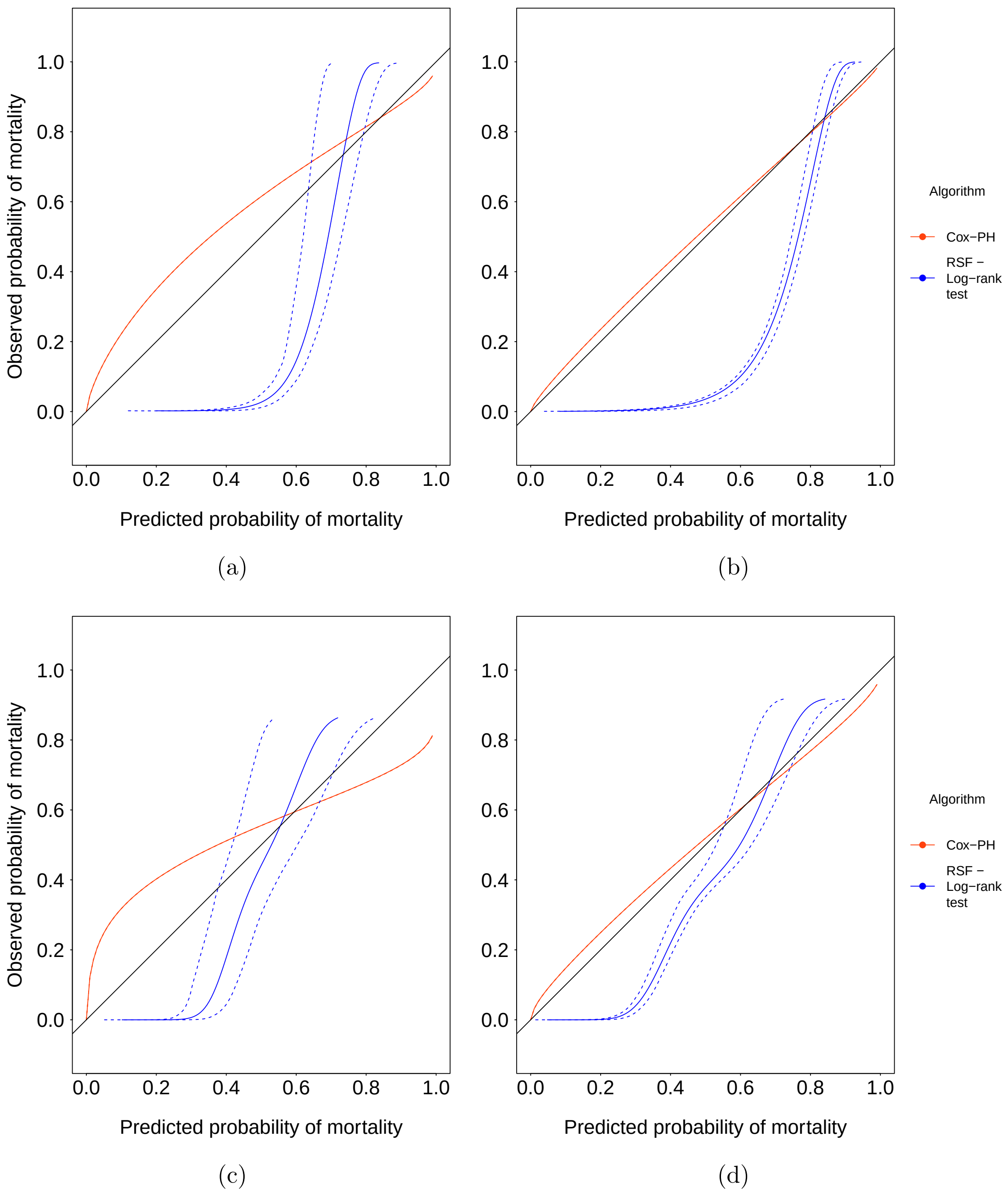

Fig. C.5b: **Calibration curves for a nonproportional hazards setting (primary biliary cirrhosis dataset).** Calibration curves at the median (50% quantile) survival time for a nonproportional hazards setting (Weibull survival time distribution $W(\lambda = 2241.74, \gamma \in \{2,5\})$), $\beta_{\text{treatment}} = -0.4$, and $n_{\text{sim}} = 500$ simulated datasets based on data without treatment-covariate interactions (primary biliary cirrhosis dataset). The solid line represents the mean calibration curve, the outer dotted lines represent the 2.5th and 97.5th percentile of the calibration curve. The black diagonal line corresponds to perfect calibration.
(a) 30% censoring, $N = 100$, (b) 30% censoring, $N = 400$,
(c) 60% censoring, $N = 100$, (d) 60% censoring, $N = 400$.
Abbreviations: Cox-PH - Cox proportional hazards model, RSF - Random survival forest.

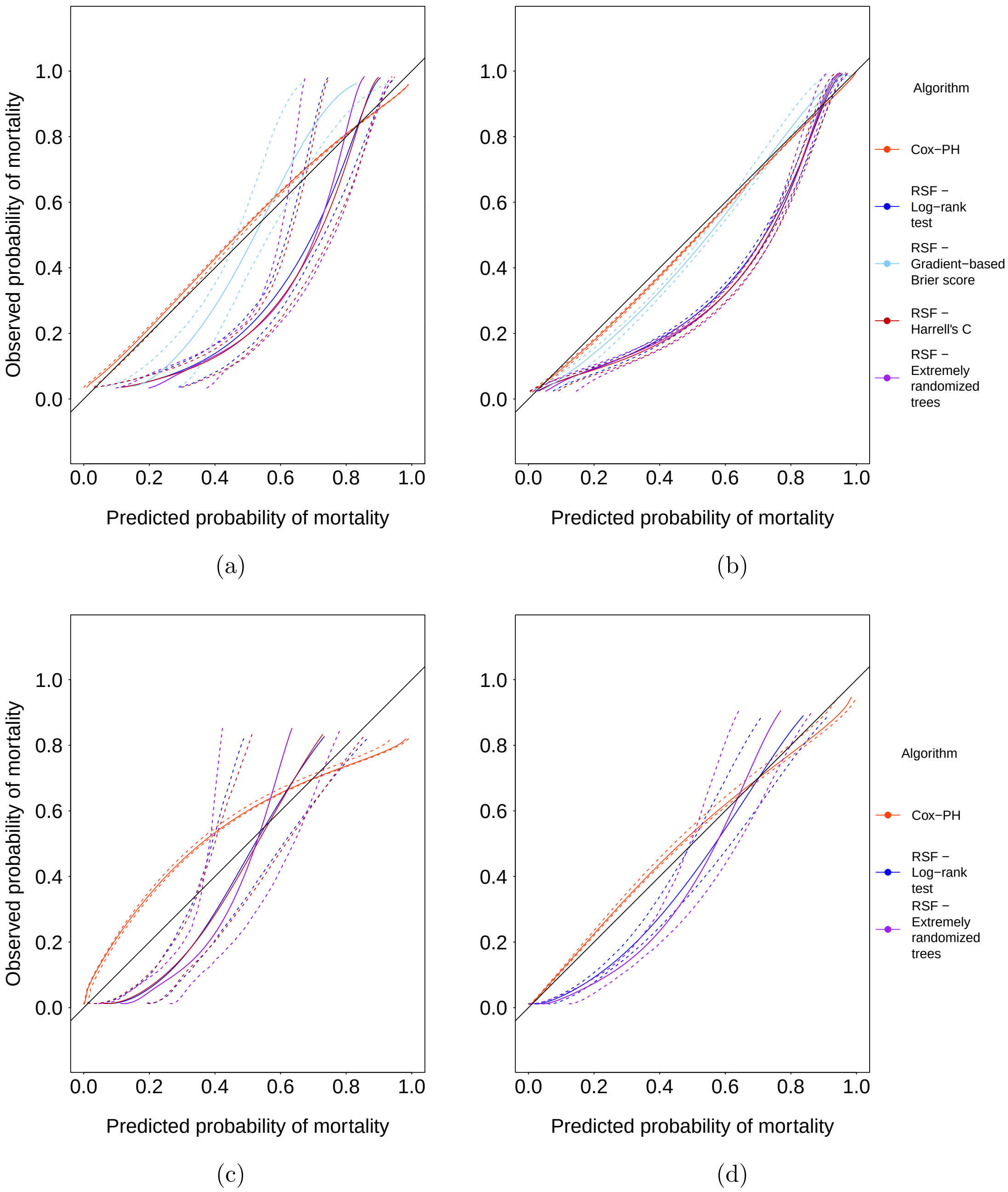

(a) (b) (c) (d)

Fig. C.6a: **Calibration curves for a proportional hazards setting (prostate cancer dataset).** Calibration curves at the median (50% quantile) survival time for a proportional hazards setting (Weibull survival time distribution $W(\lambda = 2241.74, \gamma = 1)$), $\beta_{\text{treatment}} = -0.4$, and $n_{\text{sim}} = 500$ simulated datasets based on data with three treatment-covariate interactions (prostate cancer dataset). The solid line represents the mean calibration curve, the outer dotted lines represent the 2.5th and 97.5th percentile of the calibration curve. The black diagonal line corresponds to perfect calibration.
(a) 30% censoring, $N = 100$, (b) 30% censoring, $N = 400$,
(c) 60% censoring, $N = 100$, (d) 60% censoring, $N = 400$.
Abbreviations: Cox-PH - Cox proportional hazards model, RSF - Random survival forest.

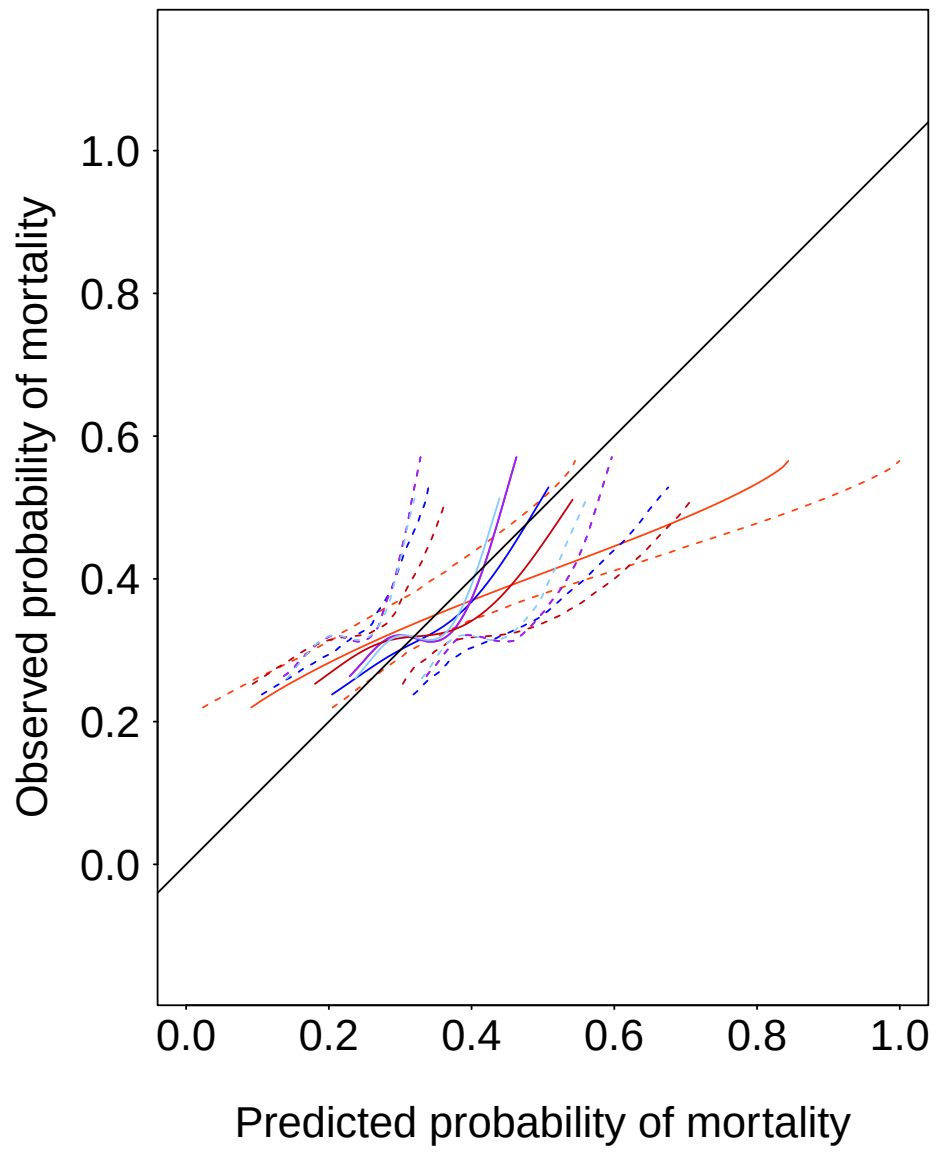

(a)

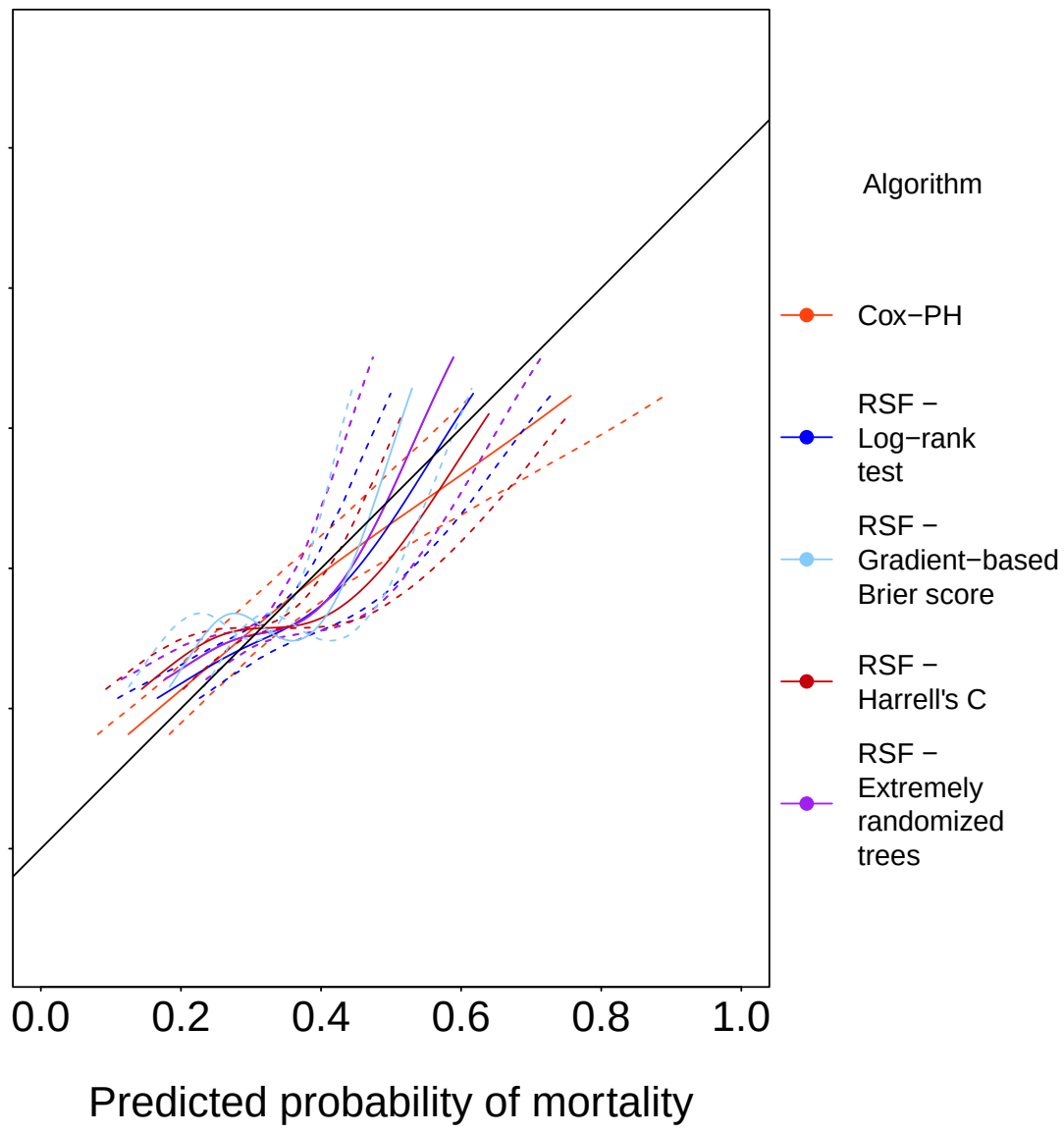

(b)

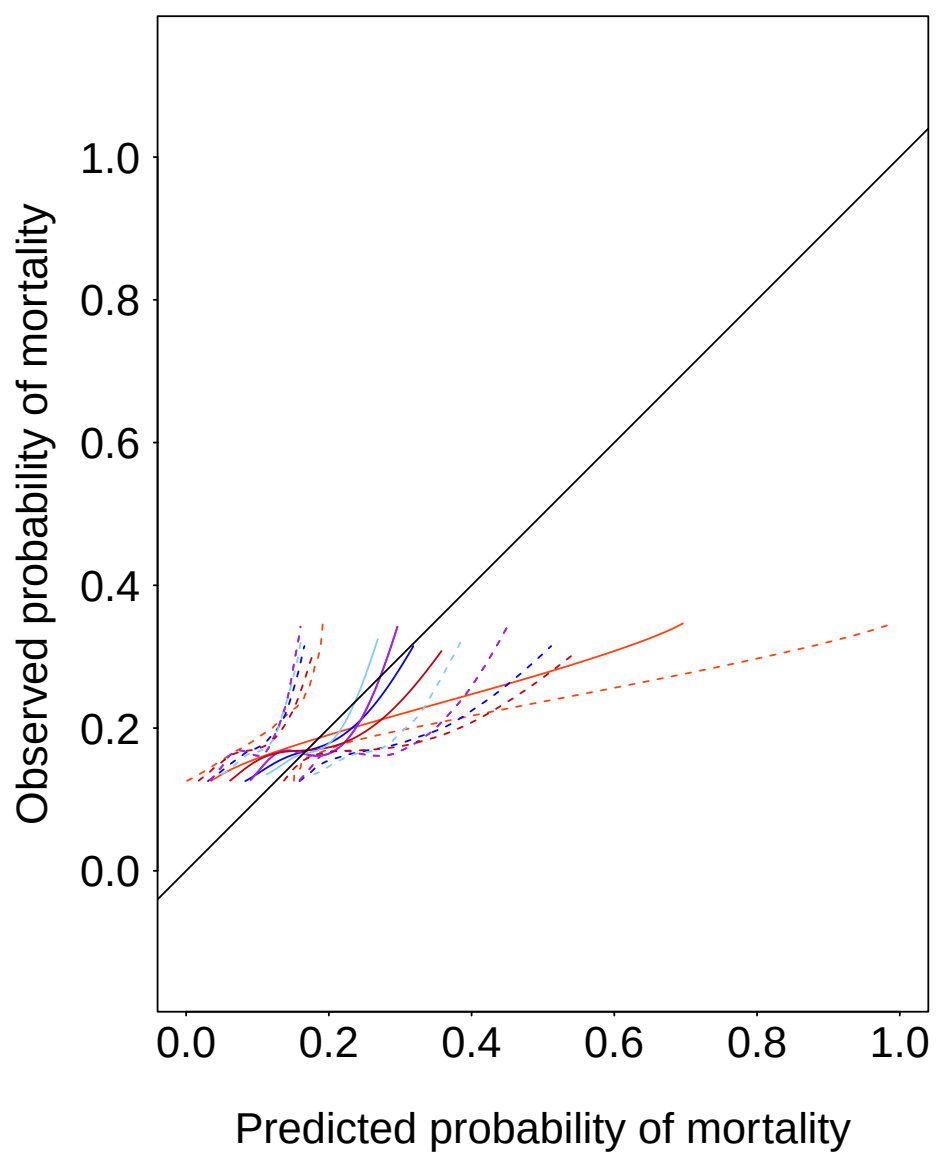

(c)

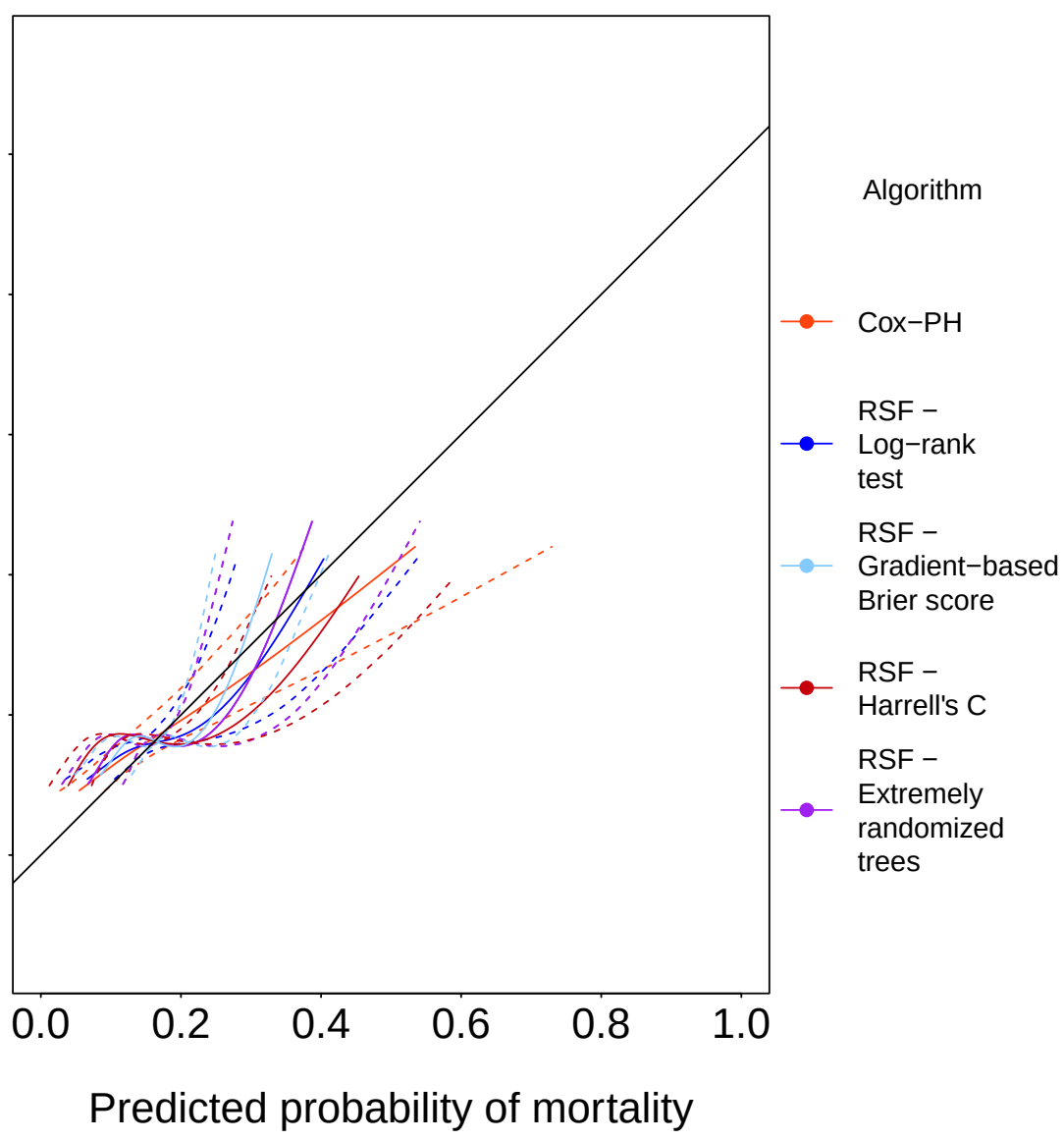

(d)

Fig. C.6b: **Calibration curves for a nonproportional hazards setting (prostate cancer dataset).** Calibration curves at the median (50% quantile) survival time for a nonproportional hazard setting (Weibull survival time distribution $W(\lambda = 39.2, \gamma \in \{2, 5\})$), $\beta_{\text{treatment}} = -0.4$, and $n_{\text{sim}} = 500$ simulated datasets based on data with three treatment-covariate interactions (prostate cancer dataset). The solid line represents the mean calibration curve, the outer dotted lines represent the 2.5th and 97.5th percentile of the calibration curve. The black diagonal line corresponds to perfect calibration.
(a) 30% censoring, $N = 100$, (b) 30% censoring, $N = 400$,
(c) 60% censoring, $N = 100$, (d) 60% censoring, $N = 400$.
Abbreviations: Cox-PH - Cox proportional hazards model, RSF - Random survival forest.

64 C.3.2: Calibration curves (complete results)

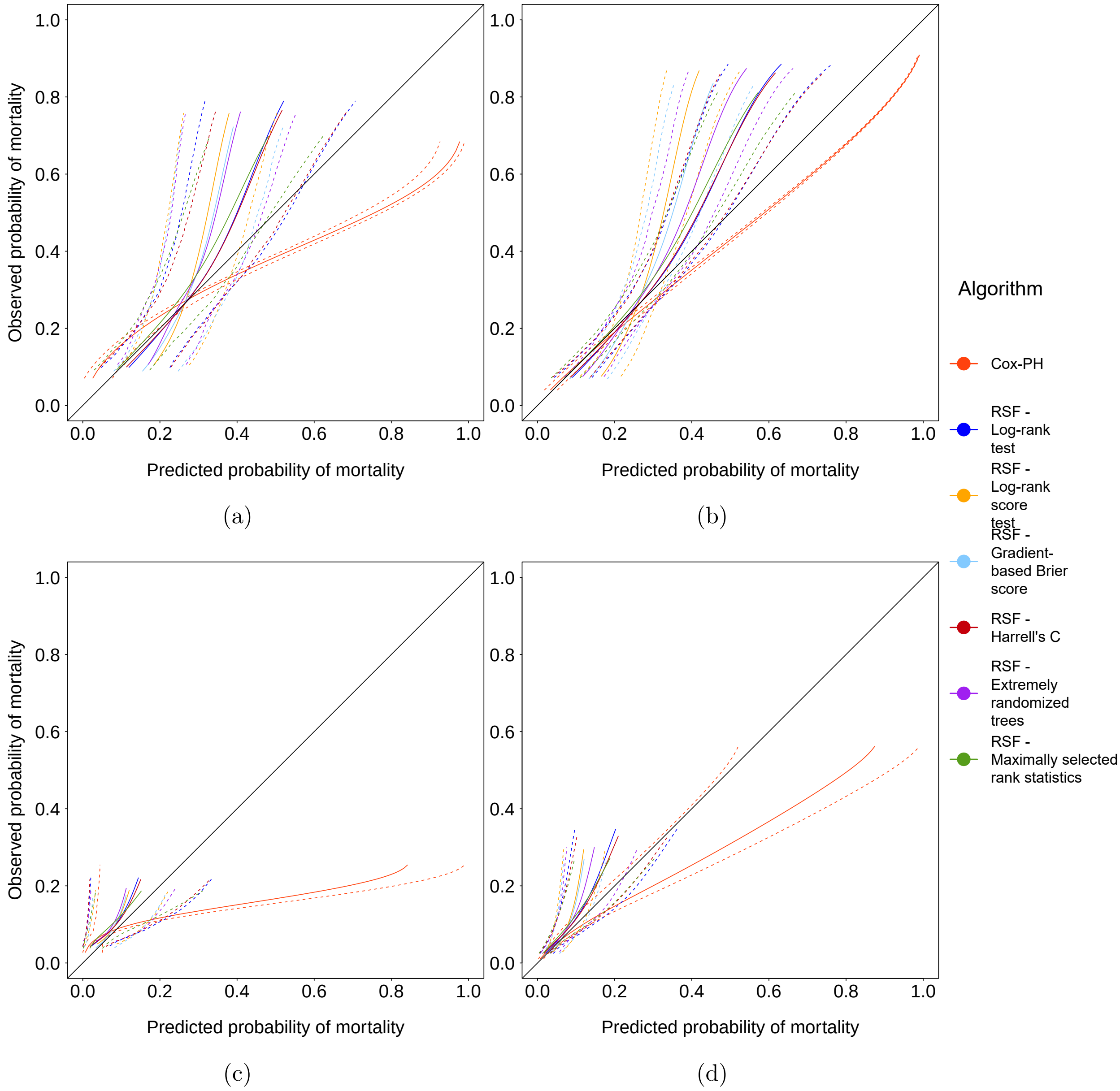

Fig. C.7a: **Calibration curves for a proportional hazards scenario (primary biliary cirrhosis dataset).** Calibration curves at the median (50% quantile) survival time for a proportional hazards setting (Weibull survival time distribution $W(\lambda = 2241.74, \gamma = 1)$ and $n_{\text{sim}} = 500$ simulated datasets based on data without treatment-covariate interactions (primary biliary cirrhosis dataset). The solid line represents the mean calibration curve, the outer dotted lines represent the 2.5th and 97.5th percentile of the calibration curve. The black diagonal line corresponds to perfect calibration.
(a) 30% censoring, $N = 100$, (b) 30% censoring, $N = 400$,
(c) 60% censoring, $N = 100$, (d) 60% censoring, $N = 400$.
Abbreviations: Cox-PH - Cox proportional hazards model, RSF - Random survival forest.

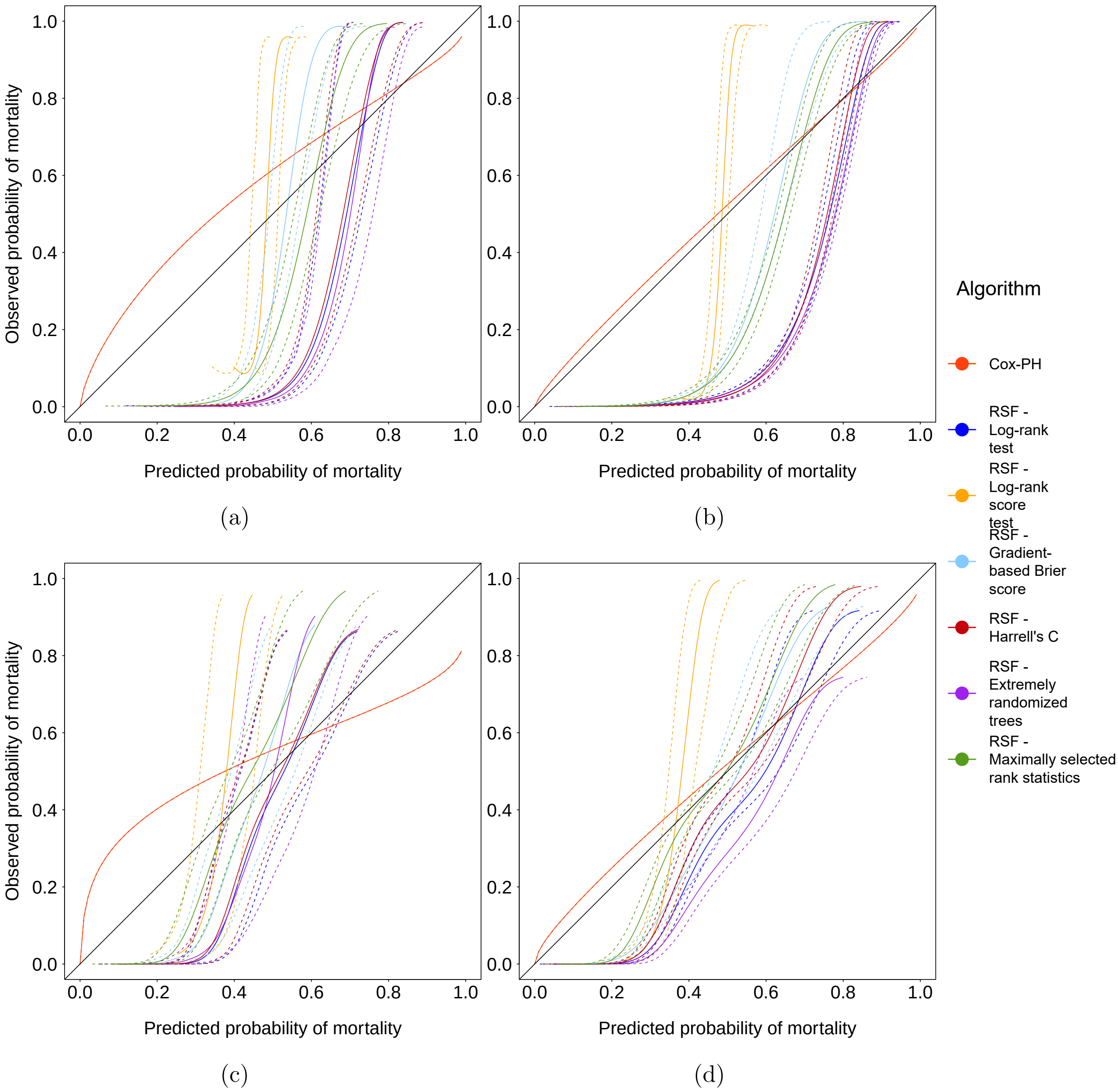

Fig. C.7b: **Calibration curves for a nonproportional hazards setting (primary biliary cirrhosis dataset).** Calibration curves at the median (50% quantile) survival time for a nonproportional hazards setting (Weibull survival time distribution $W(\lambda = 2241.74, \gamma \in \{2, 5\})$) and $n_{\text{sim}} = 500$ simulated datasets based on data without treatment-covariate interactions (primary biliary cirrhosis dataset). The solid line represents the mean calibration curve, the outer dotted lines represent the 2.5th and 97.5th percentile of the calibration curve. The black diagonal line corresponds to perfect calibration.
(a) 30% censoring, $N = 100$, (b) 30% censoring, $N = 400$,
(c) 60% censoring, $N = 100$, (d) 60% censoring, $N = 400$.
Abbreviations: Cox-PH - Cox proportional hazards model, RSF - Random survival forest.

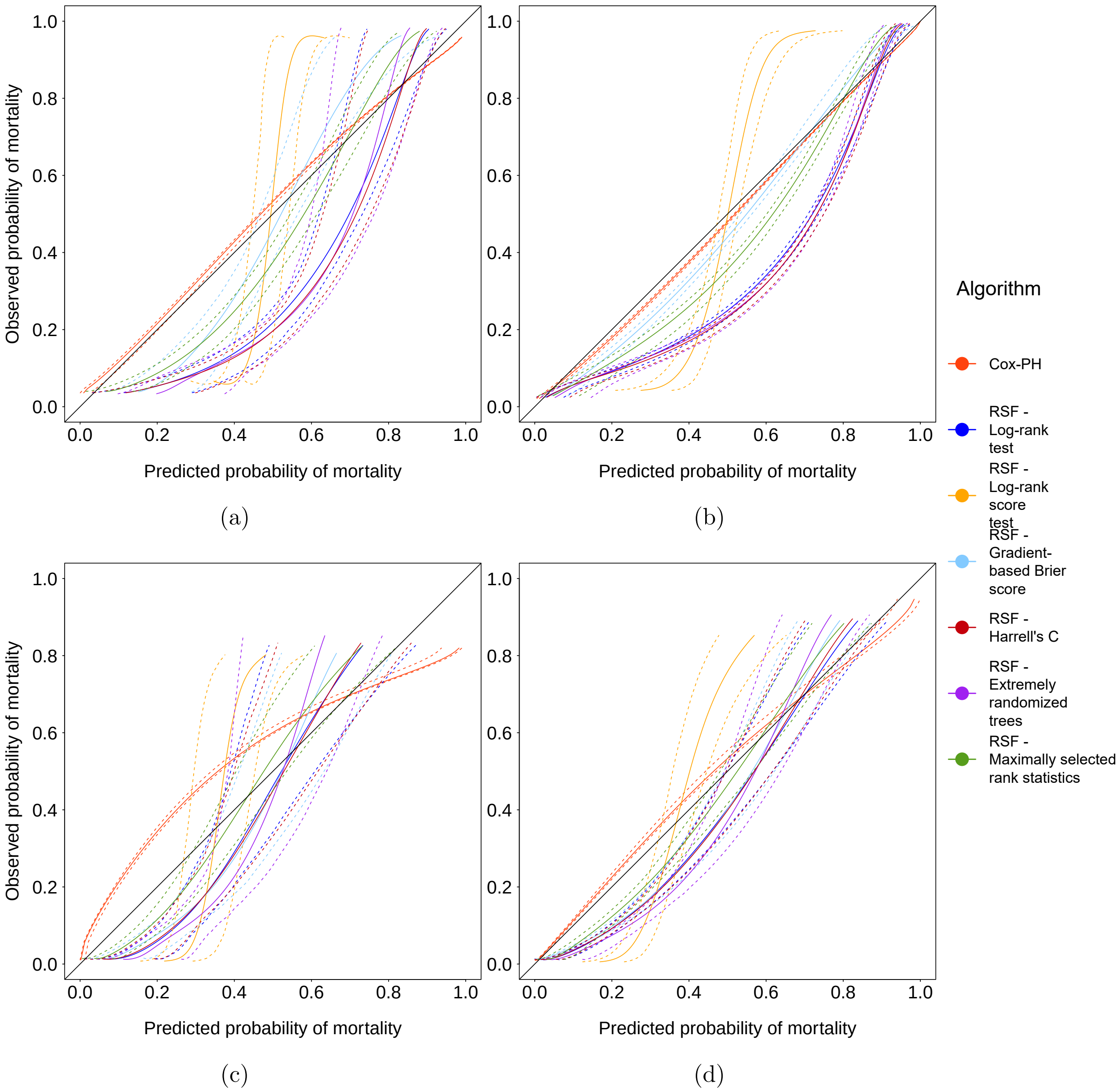

Fig. C.8a: **Calibration curves for a proportional hazards setting (prostate cancer dataset).** Calibration curves at the median (50% quantile) survival time for a proportional hazards setting (Weibull survival time distribution W($\lambda = 2241.74, \gamma = 1$)) and $n_{\text{sim}} = 500$ simulated datasets based on data with three treatment-covariate interactions (prostate cancer dataset). The solid line represents the mean calibration curve, the outer dotted lines represent the 2.5th and 97.5th percentile of the calibration curve. The black diagonal line corresponds to perfect calibration.
(a) 30% censoring, $N = 100$, (b) 30% censoring, $N = 400$,
(c) 60% censoring, $N = 100$, (d) 60% censoring, $N = 400$.
Abbreviations: Cox-PH - Cox proportional hazards model, RSF - Random survival forest.

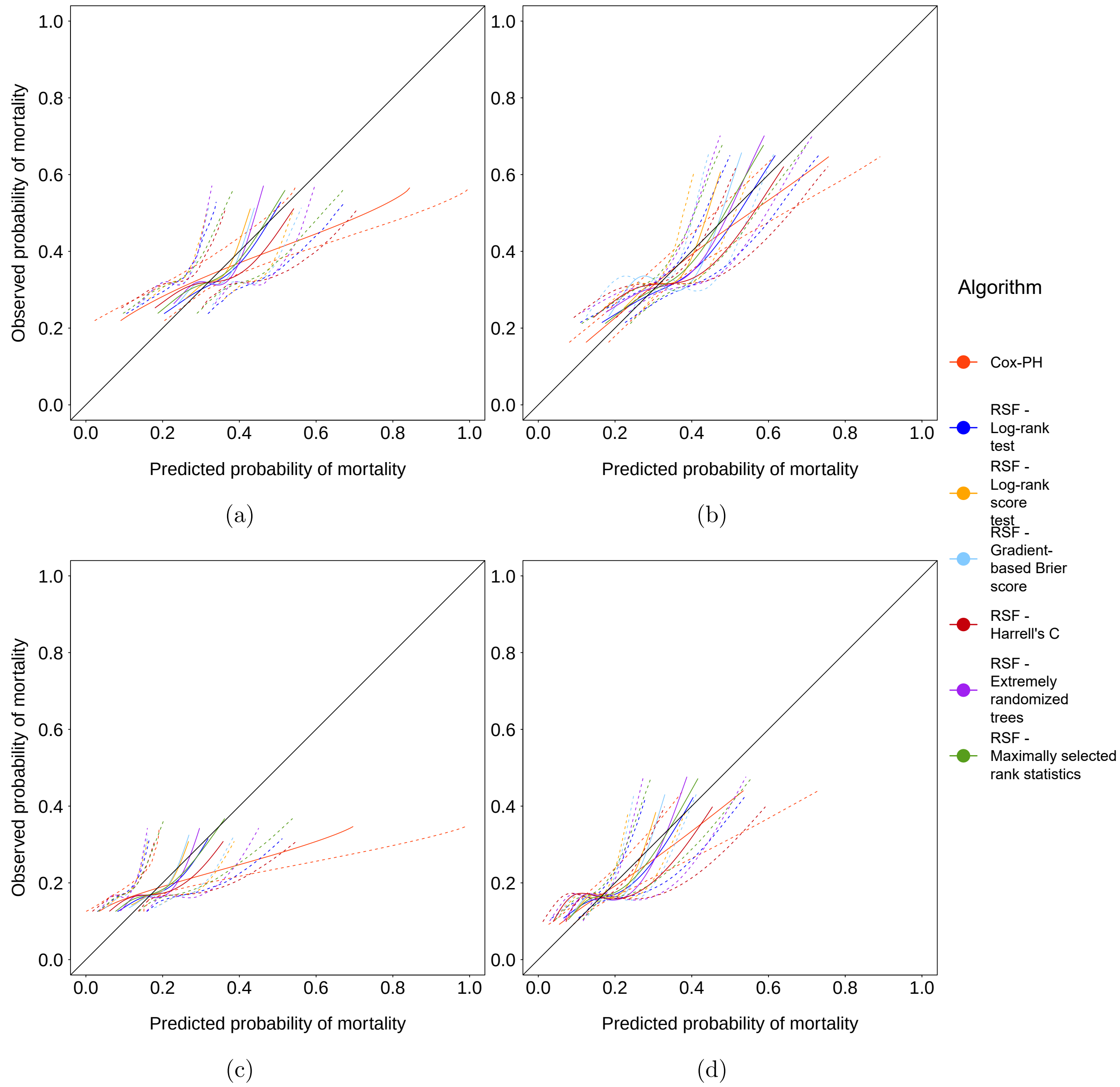

Fig. C.8b: **Calibration curves for a nonproportional hazards setting (prostate cancer dataset).** Calibration curves at the median (50% quantile) survival time for a nonproportional hazard setting (Weibull survival time distribution $W(\lambda = 39.2, \gamma \in \{2, 5\})$) and $n_{\text{sim}} = 500$ simulated datasets based on data with three treatment-covariate interactions (prostate cancer dataset). The solid line represents the mean calibration curve, the outer dotted lines represent the 2.5th and 97.5th percentile of the calibration curve. The black diagonal line corresponds to perfect calibration.
(a) 30% censoring, $N = 100$, (b) 30% censoring, $N = 400$,
(c) 60% censoring, $N = 100$, (d) 60% censoring, $N = 400$.

Abbreviations: Cox-PH - Cox proportional hazards model, RSF - Random survival forest.

List of Figures

B.1 QQ-plot (left) and plot comparing the theoretical and empirical cumulative distribution functions (right) based on maximum likelihood estimates of the respective distributional parameters for each continuous variable in the primary biliary cirrhosis dataset (no treatment-covariate interactions). 11

B.2 QQ-plot (left) and plot comparing the theoretical and empirical cumulative distribution functions (right) based on maximum likelihood estimates of the respective distributional parameters for each continuous variable in the prostate cancer dataset (three treatment-covariate interactions). 15

C.1a Distribution of the C index estimates for the scenario without treatment-covariate interactions (primary biliary cirrhosis dataset), $\beta_{\text{treatment}} = 0$, scale parameter $\lambda = 2241.74$, censoring rate 30% (a), and 60% (b). 16

C.1b Distribution of the C index estimates for the scenario without treatment-covariate interactions (primary biliary cirrhosis dataset), $\beta_{\text{treatment}} = 0.8$, scale parameter $\lambda = 2241.74$, censoring rate 30% (a), and 60% (b). 17

C.1c Distribution of the C index estimates for the scenario without treatment-covariate interactions (primary biliary cirrhosis dataset), $\beta_{\text{treatment}} = -0.4$, scale parameter $\lambda = 2241.74$, censoring rate 30% (a), and 60% (b). 18

C.2a Distribution of the C index estimates for the scenario with three treatment-covariate interactions (prostate cancer dataset), $\beta_{\text{treatment}} = 0$, scale parameter $\lambda = 39.2$, censoring rate 30% (a), and 60% (b). 19

C.2b Distribution of the C index estimates for the scenario with three treatment-covariate interactions (prostate cancer dataset), $\beta_{\text{treatment}} = 0.8$, scale parameter $\lambda = 39.2$, censoring rate 30% (a), and 60% (b). 20

C.2c Distribution of the C index estimates for the scenario with three treatment-covariate interactions (prostate cancer dataset), $\beta_{\text{treatment}} = -0.4$, scale parameter $\lambda = 39.2$, censoring rate 30% (a), and 60% (b). 21

C.3a Distribution of the Integrated Brier score (IBS) estimates for the scenario without treatment-covariate interactions (primary biliary cirrhosis dataset), $\beta_{\text{treatment}} = 0$, scale parameter $\lambda = 2241.74$, censoring rate 30% (a), and 60% (b). 22

C.3b Distribution of the Integrated Brier score (IBS) estimates for the scenario without treatment-covariate interactions (primary biliary cirrhosis dataset), $\beta_{\text{treatment}} = 0.8$, scale parameter $\lambda = 2241.74$, censoring rate 30% (a), and 60% (b). 23

C.3c Distribution of the Integrated Brier score (IBS) estimates for the scenario without treatment-covariate interactions (primary biliary cirrhosis dataset), $\beta_{\text{treatment}} = -0.4$, scale parameter $\lambda = 2241.74$, censoring rate 30% (a), and 60% (b). 24

C.4a Distribution of the Integrated Brier score (IBS) estimates for the scenario with three treatment-covariate interactions (prostate cancer dataset), $\beta_{\text{treatment}} = 0$, scale parameter $\lambda = 39.2$, censoring rate 30% (a), and 60% (b). 25
C.4b Distribution of the Integrated Brier score (IBS) estimates for the scenario with three treatment-covariate interactions (prostate cancer dataset), $\beta_{\text{treatment}} = 0.8$, scale parameter $\lambda = 39.2$, censoring rate 30% (a), and 60% (b). 26
C.4c Distribution of the Integrated Brier score (IBS) estimates for the scenario with three treatment-covariate interactions (prostate cancer dataset), $\beta_{\text{treatment}} = -0.4$, scale parameter $\lambda = 39.2$, censoring rate 30% (a), and 60% (b). 27
C.5a Calibration curves at the median survival time for the data without treatment-covariate interactions (primary biliary cirrhosis dataset) for a proportional hazard setting. 28
C.5b Calibration curves at the median survival time for the data without treatment-covariate interactions (primary biliary cirrhosis dataset) for a nonproportional hazard setting. 29
C.6a Calibration curves at the median survival time for the data with three treatment-covariate interactions (prostate cancer dataset) for a proportional hazard setting. 30
C.6b Calibration curves at the median survival time for the data with three treatment-covariate interactions (prostate cancer dataset) for a nonproportional hazard setting. 31
C.7a Calibration curves at the median survival time for the data without treatment-covariate interactions (primary biliary cirrhosis dataset) for a proportional hazard setting. 32
C.7b Calibration curves at the median survival time for the data without treatment-covariate interactions (primary biliary cirrhosis dataset) for a nonproportional hazard setting. 33
C.8a Calibration curves at the median survival time for the data with three treatment-covariate interactions (prostate cancer dataset) for a proportional hazard setting. 34
C.8b Calibration curves at the median survival time for the data with three treatment-covariate interactions (prostate cancer dataset) for a nonproportional hazard setting. 35

List of Tables

A.1 Overview of currently available splitting rules for the RSF in the `R` packages `randomForestSRC` (Ishwaran et al., 2008) and `ranger` (Wright et al., 2023) compared in the simulation study. 3
B.1a Correlation matrix of the variables in the primary biliary cirrhosis dataset used in the copula model. 4

B.1b Correlation matrix of the variables in the RCT in prostate cancer patients used in the copula model. 5
B.2a Univariate distributions from which covariate values are simulated (primary biliary cirrhosis dataset). 6
B.2b Univariate distributions from which covariate values are simulated (prostate cancer dataset). 6

References

Boehmke, B. and Greenwell, B. (2019). *Hands-On Machine Learning with R*. Chapman & Hall/CRC, The R Series, 1 edition.

Efthimiou, O., Seo, M., Chalkou, K., Debray, T., Egger, M., and Salanti, G. (2024). Developing clinical prediction models: a step-by-step guide. *British medical journal*, 386.

Geurts, P., Ernst, D., and Wehenkel, L. (2006). Extremely randomized trees. *Machine Learning*, *63*:3–42.

Hothorn, T. and Lausen, B. (2003). On the exact distribution of maximally selected rank statistics. *Computational Statistics and Data Analysis*, *43*(2):121–137.

Ishwaran, H., Kogalur, U., Blackstone, E., and Lauer, M. (2008). Random Survival Forests. *The Annals of Applied Statistics*, *2*:841–60.

Ishwaran, H. and Kogalur, U. B. (2023). *randomForestSRC: Fast Unified Random Forests for Survival, Regression, and Classification (RF-SRC)*. CRAN. https://CRAN.R-project.org/package=randomForestSRC

Ishwaran, H. and Lu, M. (2019). *Random Survival Forests*, pages 1–13.

Ripley, B., Venables, B., Bates, D. M., Hornik, K., Gebhardt, A., and Firth, D. (2024). *MASS: Support Functions and Datasets for Venables and Ripley's MASS*. CRAN. https://CRAN.R-project.org/package=MASS

Schmid, M., Wright, M. N., and Ziegler, A. (2016). On the use of Harrell's C for clinical risk prediction via Random Survival Forests. *Expert Systems with Applications*, *63*:450–459.

Wright, M. N., Dankowski, T., and Ziegler, A. (2016). Unbiased split variable selection for Random Survival Forests using maximally selected rank statistics. *Statistics in Medicine*, *36*:1272 – 1284.

Wright, M. N., Wager, S., and Probst, P. (2023). *ranger: A Fast Implementation of Random Forests*. CRAN. https://CRAN.R-project.org/package=ranger
